# Identification and dereplication of endophytic *Colletotrichum* strains by MALDI TOF mass spectrometry and molecular networking

**DOI:** 10.1038/s41598-020-74852-w

**Published:** 2020-11-13

**Authors:** Morgane Barthélemy, Vincent Guérineau, Grégory Genta-Jouve, Mélanie Roy, Jérôme Chave, Régis Guillot, Léonie Pellissier, Jean-Luc Wolfender, Didier Stien, Véronique Eparvier, David Touboul

**Affiliations:** 1 Université Paris-Saclay, CNRS, Institut de Chimie Des Substances Naturelles, UPR 2301, Avenue de la Terrasse, 91198 Gif-sur-Yvette, France; 2 UMR 8038 CiTCoM, Faculté de Pharmacie de Paris, Université Paris Descartes, Avenue de l’observatoire, 75006 Paris, France; 3 Molecules of Communication and Adaptation of Microorganisms (UMR 7245), National Museum of Natural History, CNRS, Paris, France; 4 Laboratoire Evolution Et Diversité Biologique, UPS- CNRS - IRD - UMR 5174, Université Paul Sabatier (Toulouse 3), 118 route de Narbonne, 31062 Toulouse, France; 5 ICMMO, Université Paris-Saclay - UMR CNRS 8182, Rue du Doyen Georges Poitou, 91405 Orsay, France; 6 School of Pharmaceutical Sciences, Institute of Pharmaceutical Sciences of Western Switzerland, University of Geneva, Rue Michel Servet 1, CH-1211 Geneva 4, Switzerland; 7 Sorbonne Université, CNRS, Laboratoire de Biodiversité et Biotechnologies Microbiennes, USR3579, Observatoire Océanologique, Banyuls-sur-Mer, France

**Keywords:** Mass spectrometry, Fungi, Natural products

## Abstract

The chemical diversity of biologically active fungal strains from 42 *Colletotrichum*, isolated from leaves of the tropical palm species *Astrocaryum sciophilum* collected in pristine forests of French Guiana, was investigated. The collection was first classified based on protein fingerprints acquired by matrix-assisted laser desorption/ionization time-of-flight mass spectrometry (MALDI-TOF MS) correlated with cytotoxicity. Liquid chromatography coupled to high-resolution tandem mass spectrometry (LC-HRMS/MS) data from ethyl acetate extracts were acquired and processed to generate a massive molecular network (MN) using the MetGem software. From five *Colletotrichum* strains producing cytotoxic specialized metabolites, we predicted the occurrence of peptide and cytochalasin analogues in four of them by MN, including a similar ion clusters in the MN algorithm provided by MetGem software. Chemoinformatics predictions were fully confirmed after isolation of three pentacyclopeptides (cyclo(Phe-Leu-Leu-Leu-Val), cyclo(Phe-Leu-Leu-Leu-Leu) and cyclo(Phe-Leu-Leu-Leu-Ile)) and two cytochalasins (cytochalasin C and cytochalasin D) exhibiting cytotoxicity at the micromolar concentration. Finally, the chemical study of the last active cytotoxic strain BSNB-0583 led to the isolation of four colletamides bearing an identical decadienamide chain.

## Introduction

In the field of bioactive natural products research, the chemical diversity of microorganisms is increasingly explored^1^. Microorganisms virtually occupy every living habitat on earth, and endophytes have long been thought to be a prime source of original chemical compounds^2^. These symbiotic microorganisms are ubiquitous in plants, living inside the tissues of a host without causing any apparent harm^3^. Tropical tree leaves are considered as hotspot for endophytes^4^. Because of their high diversity, it has been clearly established that they can serve as reservoirs of new bioactive specialized metabolites^5^. Indeed, endophytic strains have been shown to contribute to plant defences by preventing herbivory^6^ and invasion from superficial pathogens^3^. Therefore, a significant proportion of endophytic extracts are thought to be cytotoxic and/or antimicrobial.

Among fungal endophytes, the genus *Colletotrichum* is represented by a large number of species widely distributed in the tropics but also in temperate regions^7^. Some *Colletotrichum* are predominant in living plant tissues as symptomless endophytes^8,9^. The presence of *Colletotrichum tofieldiae* in roots has been shown to enhance plant fitness^10^, but other species are pathogenic. Indeed, *Colletotrichum* spp. are also among the most important groups of plant pathogens^11^. Several studies have sought to advance the systematic classification of *Colletotrichum* and to propose better identification of the species using DNA sequencing methods^12,13^. Among them, Douanla-Meli and Unger used high-throughput sequencing on selected markers for 454 *Colletotrichum* spp. isolates to identify strains at the species level^14^. Nevertheless, there is a growing interest in developing a fast and efficient method to identify and classify strains^15^ to reduce the required cost and time. Over the last decade, matrix-assisted laser desorption/ionization time-of-flight mass spectrometry (MALDI-TOF MS) has been implemented in hospital centres as the method of choice for microbiology diagnosis. This method compares microbial peptide/protein fingerprint against reference databases^16^. To improve classification when using large microorganism collections, machine learning technique, such as the Aristotle Classifier, has been introduced in the literature^17^. Moreover, the identification accuracy is increased by combining the data from MALDI-TOF MS and FTIR spectroscopy and hierarchical clustering analysis^18^. Alternatives to MALDI profiling has been recently introduced by the group of Takats demonstrating that automated laser-assisted rapid evaporative ionisation mass spectrometry (LAREIMS) platform can chemically screen over 450 yeast colonies in under 4 h, while simultaneously generating recoverable glycerol stocks of each colony in real-time^19^. First dedicated to the identification of human pathogenic or opportunistic yeasts and bacteria, some studies recently developed spectral databases for a more systematic identification of filamentous fungi^20,21^. Nevertheless, these databases still focus on clinical isolates. Few studies reported MALDI-TOF MS as a tool for strain identification for environmental microorganisms^22^ and demonstrated the correlation of MALDI classification of microorganism together with production of specialized metabolites^23^.

Forty-two endophytic and cultivable *Colletotrichum* strains from healthy leaves of a tropical palm species, *Astrocaryum sciophilum* Miq. (Pulle), Arecaceae were studied. *A. sciophilum* is a long-lived palm that grows in the understory of tropical forests of the Guiana Shield of Amazonia, and can be locally abundant^24^. A methodology was developed to acquire protein MS profile of *Colletotrichum* strains by MALDI-TOF MS to decipher the variability of our collection. *Colletotrichum* strains with similar phenotypes were recognized thereby reducing the number of strains of interest. In parallel, strains were extracted with ethyl acetate (EtOAc) and each extract was tested on cell viability bioassays for cytotoxic activity. Five unique cytotoxic *Colletotrichum* extracts were further investigated by the molecular network approach^25–27^ and the new t-SNE (t-distributed stochastic neighbor embedding) algorithm provided by MetGem software^28^. This study led to the isolation of known metabolites (cyclopeptides and cytochalasins) and of an unprecedented series of new metabolites designated as colletamides.

## Results and discussion

Forty-two *Colletotrichum* extracts were produced from endophytes isolated on five *Astrocaryum sciophilum* palm specimens. We then quantified the cell viability of the EtOAc extract on MRC-5 cells lines (Table S1). Among the 42 extracts, twelve induced less than 35% of cell viability at 10 µg/mL and were designated as cytotoxic, while cell viabilities were over 65% at 10 µg/mL for the remaining ones. All strains were sequenced using a universal DNA marker of the ITS ribosomal cluster. Most strains were related to *Colletotrichum gloeosporioides* based on the closest ITS sequences according to the BLAST tool on NCBI (Table S1). Second or third closest sequences mostly refer to other *Colletotrichum* species, such as *C. fructicola*, *C. karstii or C. vietnamense* with no clear strain clustering. A phylogenetic tree was built using the ITS sequences of each isolated strain based on a neighbour-joining analysis of an alignment of ITS sequences with 1000 bootstrap replicates (Figure S1). First, it confirmed that an accurate identification at the species level remained impossible based on the ITS region sequencing only due to highly similar sequences on this particular genomic region.

Second, there is no obvious correlation between the ITS-based phylogeny and the biological activity profile. Moreover, strains in the same clade were not necessarily morphologically similar (Figure S2). Given this level of variability, and in order to search for bioactive metabolites in a large culture collection, we decided to generate a classification that is more reflective of strain secondary metabolism rather than taxonomical classification.

**Figure 1:**
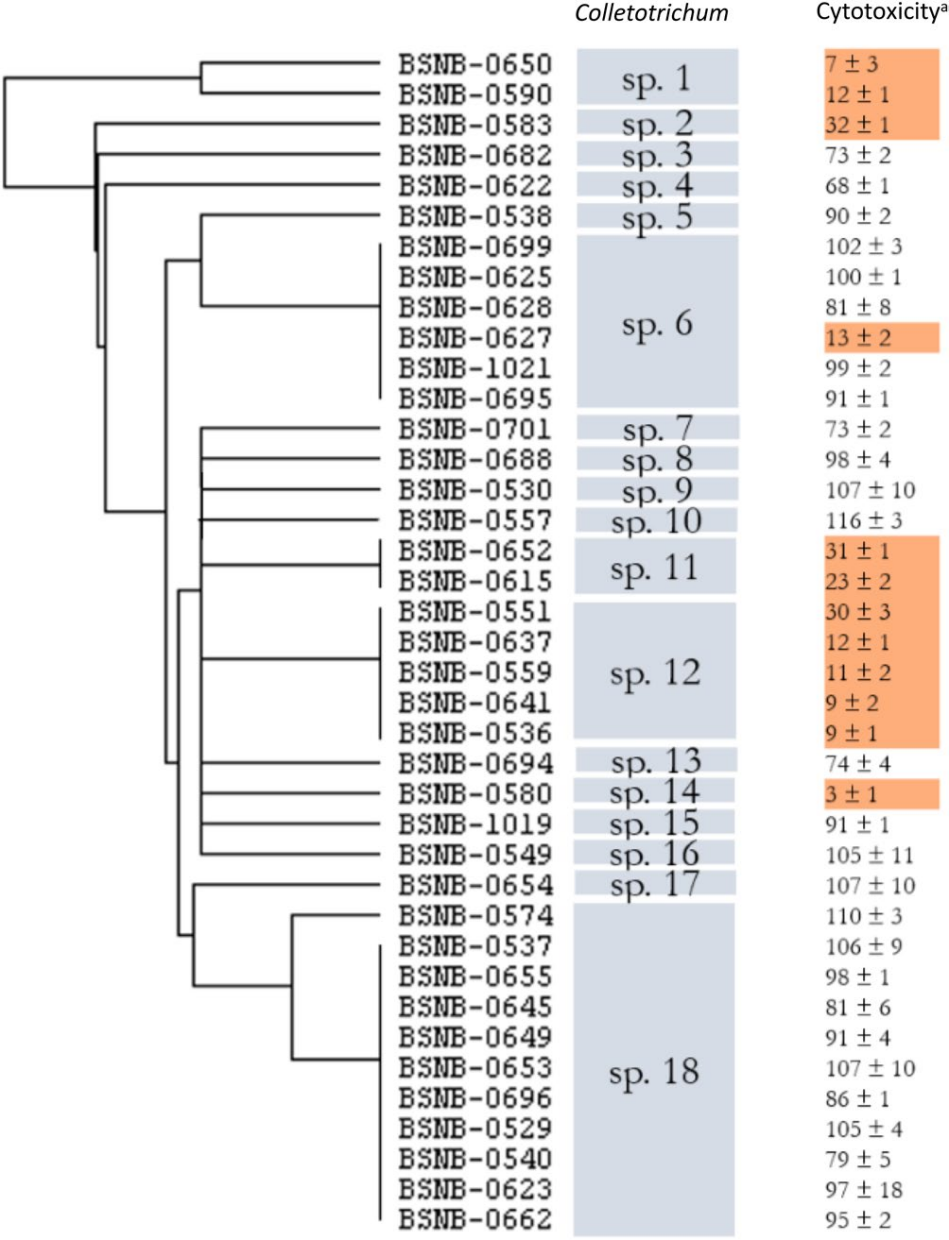
Evaluation of the correlation between mass spectrometry profile classification and cytotoxic activities of the 42 Colletotrichum strains. Hierarchical cluster tree derived from the comparison of the protein fingerprint given by the analysis on MALDI TOF MS (most intense ions analysis). ^a^Cytotoxicity of the EtOAc extract of each strain at 10 µg/ml: viability of MRC-5 cells. Cytotoxic extracts were highly correlated with Colletotrichum sub-species.

For each isolate, a protein extract was analyzed by MALDI-TOF MS. Fingerprints showed that some of our *Colletotrichum* isolates share the same protein profiles whereas fifteen strains are characterized by a unique one (Figure S1 and Figure S3-S22). Considering previous reports on strain identification by MALDI-TOF MS and the specificity of protein fingerprints among species^21,22^, we assumed that each protein fingerprint refers to a Operational Chemically Unit (in reference and comparison with Operational Taxonomic Unit in genetic or OTU) within the genus. This expectation has been confirmed through the classification by MALDI-TOF protein profiling and hierarchical clustering data analyses of the complete collection of environmental microorganisms at CNRS-ICSN, containing more than 1000 unique strains. Thus, from the 20 different protein fingerprints drawn from the 42 *Colletotrichum* isolates (Figure S1), our collection can be realigned into 18 different chemotypes OCU. These OCUs (noted from *Colletotrichum* sp.1 to *Colletotrichum* sp.18) are classified following a hierarchical clustering based on their protein fingerprint (Fig. 1). Inactive BSNB-0703, BSNB-0290 and BSNB-0646 isolates do not appear in this hierarchical clustering because of the low quality of the recorded spectra.

**Figure 2:**
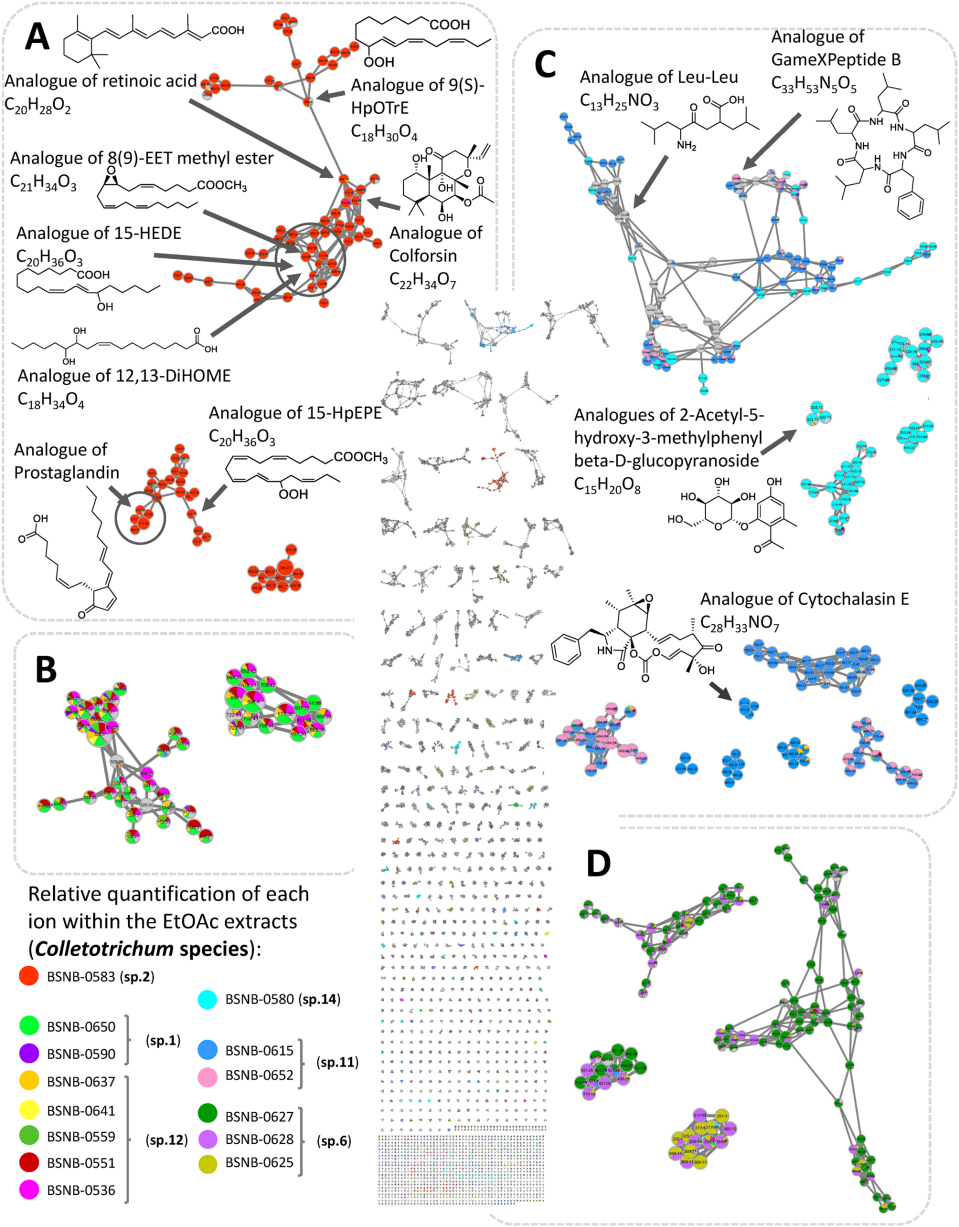
Massive molecular network of the 42 extracts from *Astrocaryum sciophilum* endophytes. MN is reduced to the ions with m/z between 200 and 900 and the selfloop nodes were removed to give a MN of 6316 nodes. Boxes (**A**–**D**) regroup clusters of ions from the MN: Relative quantification of each ion was represented as pie chart diagrams, the proportions of which were based on respective areas of the corresponding extracted ion chromatogram (XIC) area. MetGem software (https://metgem.github.io/).

The chemical diversity of extracts was analyzed to prioritize the isolation of active metabolites. To this end, a massive molecular network (MN) was generated from the liquid chromatography coupled to high-resolution tandem mass spectrometry (LC-HRMS/MS) data acquired from the EtOAc extracts of our 42 *Colletotrichum* isolates (Fig. 2 and S23–S24). To facilitate the visualization of bioactive ion clusters, each cytotoxic extract was associated to a colour on the MN (Fig. 2). For each node (couple of a particular MS/MS spectrum and retention time), the repartition of compounds across the different extracts is depicted as a pie chart diagram based on these colours.

Cytotoxic extracts of three OCU: *Colletotrichum* sp.2, sp.14 and sp.11 displayed noticeably specific ion clusters (Fig. 2A,C). All extracts of *Colletotrichum* sp.1 and sp.12 shared similar ion clusters in the MN, respectively (Fig. 2B). Secondly, although BSNB-0627 was the only bioactive strain from OCU *Colletotrichum* sp.6 sub-collection, its chemical profile did not differ from isolates with the same protein fingerprint such as BSNB-0625 and BSNB-0628 (Fig. 2D). The MN did not explain the different biological activities among the OCU *Colletotrichum* sp.6 strains. BSNB-0627 was then excluded from further chemical investigation. As OCU *Colletotrichum* sp.1 and sp.12 had the same metabolite composition, we finally focused on four different metabolite profiles coming from the five cytotoxic taxa.

**Figure 3:**
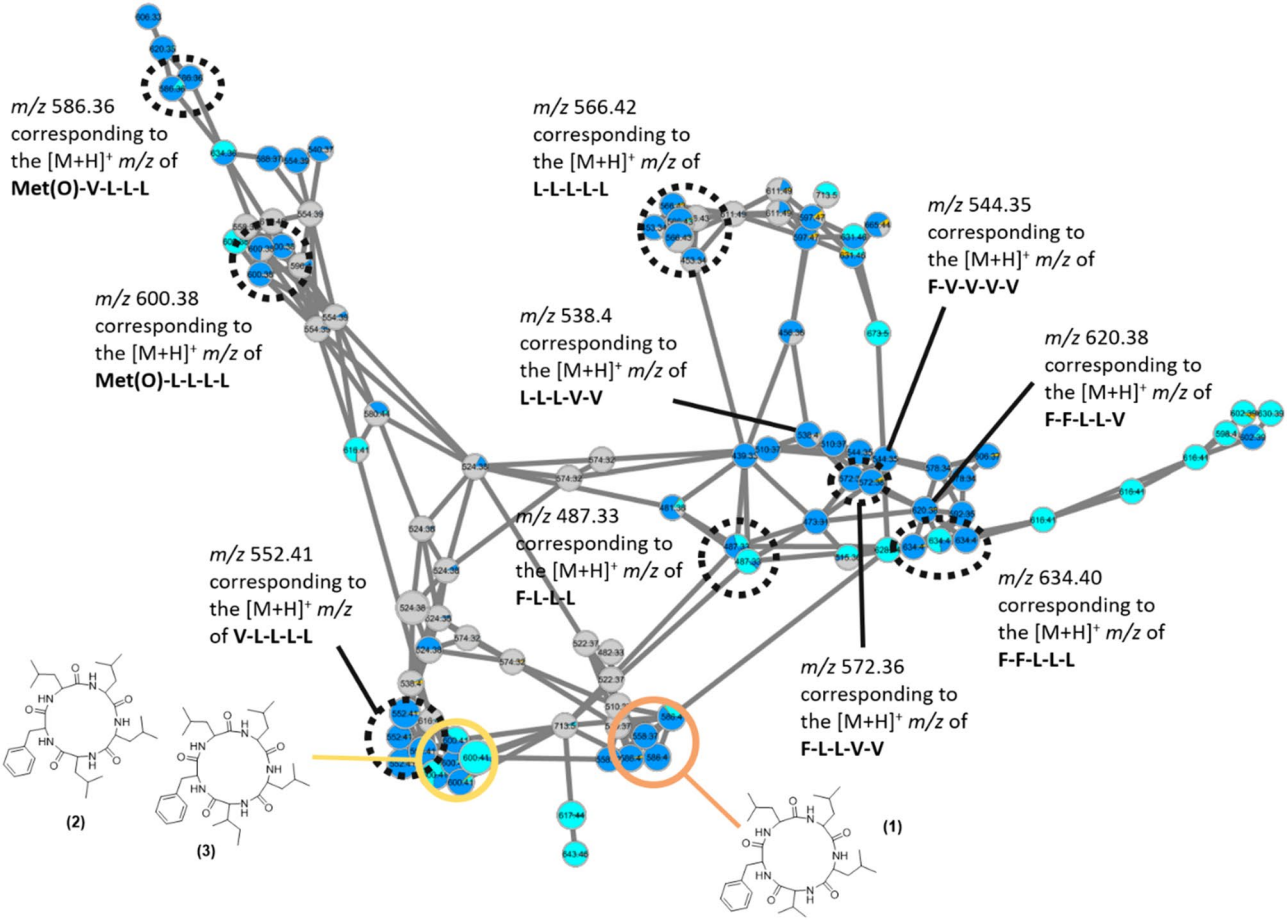
Cyclopeptide composition in amino acids based on the *m/z* mass of protonated molecules and the *m/z *mass of amino acid immonium. Isolated compounds **1**, **2** and **3** in the cluster are also associated to their corresponding nodes. MetGem software (https://metgem.github.io/).

**Figure 4:**
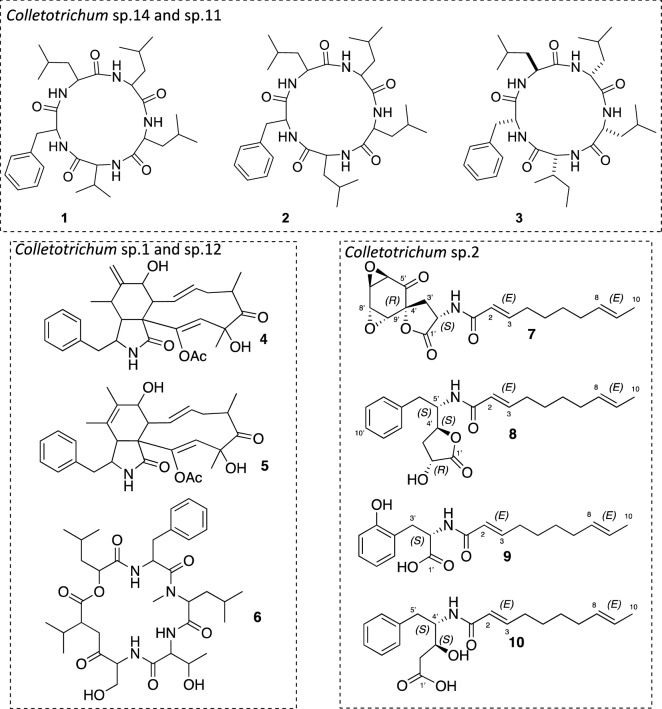
Molecules isolated form different Colletotrichum species of our collection.

The study of OCU *Colletotrichum* sp.14 and sp.11 extracts was undertaken by searching known compounds and analogues in the MS/MS databases available from the MetGem software^26,29^. Thanks to this methodology, cyclopeptide analogues could be annotated (Fig. 2C and 3). After a scaled-up culture of BSNB-0580, three cyclopeptides were isolated from the EtOAc extract and identified by NMR, MS/MS analysis and manual fragmentation assignments. The amino acid composition was deduced from the immonium ions in the low mass range of the MS/MS spectra^30^: ions at *m/z* 86.097 (Leucine/Isoleucine), *m/z* 72.081 (Valine) and *m/z* 120.081 (phenylalanine) were unambiguously attributed. Structures of isolated cyclopeptides cyclo(Phe-Leu-Leu-Leu-Val) **(1)**, cyclo(Phe-Leu-Leu-Leu-Leu) **(2)** and cyclo(Phe-Leu-Leu-Leu-Ile) **(3)** were then refined by NMR (Fig. 4).

The relative configuration of cyclo(Phe-Leu-Leu-Leu-Ile) was determined by X-ray crystallography (Figure S81). By analysing the clusters of nodes related to these cyclopeptides (Fig. 4), we observed that two isomers of compounds **2** and **3** can be produced by the strain BSNB-0580 whereas three isomers of compound **1** were also produced, certainly with differences in the amino acid sequence or in the proportion leucine/isoleucine. Furthermore, two nodes at *m/z* 600.38 and 586.36 matched with peptides bearing oxidized methionine due to the detection of an immonium ion signal at *m/z* 120.048. Thus, we can assume that the two compounds are composed of one oxidized methionine and four leucines (or isoleucines) for the first one, and one oxidized methionine, one valine and three leucines (or isoleucines) for the second one. Moreover, other peptides composed of various number of phenylalanines, leucines/isoleucines and valines were annotated based on the HRMS and the MS/MS fragmentation. Finally, nodes belonging to peptides bearing non-proteinogenic amino acids were annotated based on unusual *m/z* values of immonium ions and differences between b and y-ion products. These cyclopeptides have not been isolated and formally identified because of their low abundance in the strain extract.

**Figure 5:**
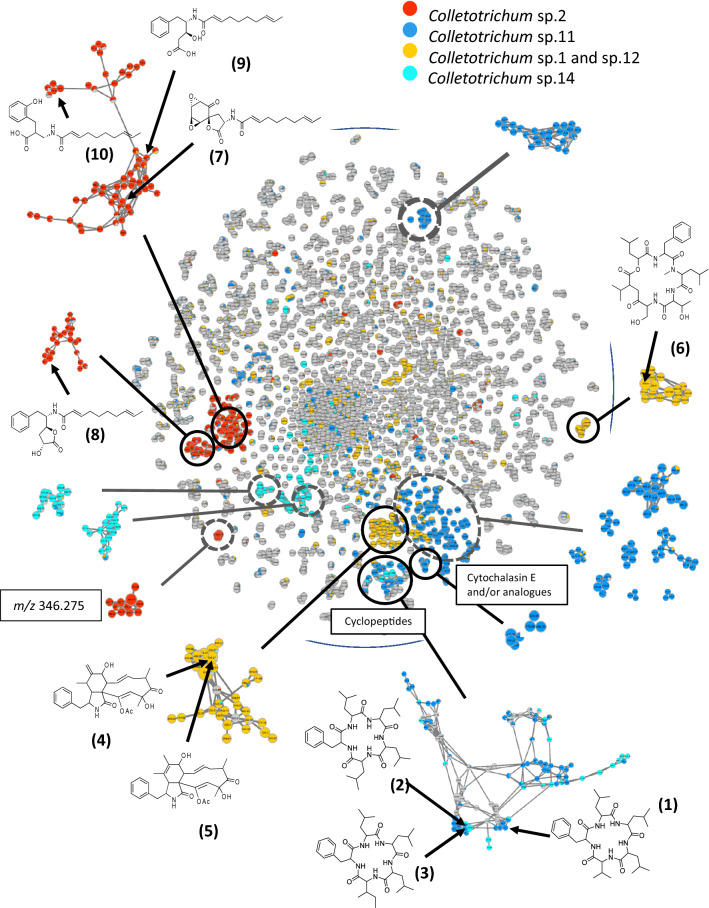
t-SNE representation of the massive Molecular Network of 42 extracts of *Colletotrichum* sp. Relative quantification of each ion was represented as pie chart diagrams, with proportions based on respective peak areas in MS. Some zones are associated with clusters of the MN by comparison of the nodes. These clusters can be visualised around the t-SNE representation. Results of the former dereplication of the MN was annotated with a t-SNE zone corresponding to analogues of peptides and another zone to analogues of Cytochalasin E. Metabolites isolated and identified from the study of three Colletotrichum strains are associated to their corresponding node in the MN. MetGem software (https://metgem.github.io/).

The MN approach was unable to annotate metabolites clusters specifically related to OCU *Colletotrichum* sp.12 and sp.1 (Fig. 2B). Indeed, the MN approach ranks the clusters by size, from the largest to the smallest (self-loop), but does not give access to the inter-cluster distance. To overcome this limitation, data analysis using the t-SNE algorithm of the MetGem software (Fig. 5) regroups ions with related scaffolds that had been separated into different clusters by the MN^28^. The t-SNE method shows that ions from a MN cluster of *Colletotrichum s*p.12 and sp.1 are spatially close to ions annotated as analogues of peptides and cytochalasin E (Fig. 5). After searching standards in the MS/MS databases, a node at *m/z* 496.2326 was putatively assigned to protonated cytochalasin E ([M + H]^+^). Assignment of the two related clusters was performed after cultivation of the strain BSNB-0536 (OCU *Colletotrichum* sp.12) on the large scale and isolation of cytochalasin D (**4**), cytochalasin C (**5**), and the cyclohexadepsipeptide hirsutatin A (**6**). The spectroscopic data for these compounds were identical to those provided in the literature^45,50^. These results demonstrate the interest of the t-SNE prediction module for propagating nodes annotation.

Finally, according to the classical and t-SNE MN, the extract of OCU *Colletotrichum* sp.2 (BSNB-0583) contained specialized metabolites specific to this strain distributed into two related clusters. Targeted isolation of the strain-specific metabolites provided 4 compounds (**7**–**10**) in pure form (Fig. 4). Compound **7** molecular formula was determined as C_19_H_23_NO_6_ based on ESI-HRMS analysis with a protonated molecule at *m/z* 362.1621 [M + H]^+^ (calcd for C_19_H_24_NO_6_^+^, 362.1598, mass error 6.35 ppm). According to NRM data analysis (see supplementary information), compounds **7** was therefore analogous to aranorosin^31^. It has been referenced in a patent before as a potential inhibitor of the 3-hydroxy-3-methyl-glutaryl-coenzyme A reductase^32^. However, the source organism was claimed to be an unidentified fungus (ATCC 20,953), the stereochemistry was not specified and the only analytical data provided was a list of ^13^C NMR chemical shifts. This list suggests that their compound C and our compound **7** may have been identical. We determined the relative configuration in compound **7** combining NOE NMR data DP4 approach^33^ (see supplementary information). A very high probability (99.5%) was obtained for the relative configuration 2′S*,4′S*,5′S*,8′S*-**7** (Figure S48). The *2′S,4′S,5′S,8′S*-**7** absolute configuration of **7**, was deduced by comparison of the predicted and experimental ECD spectra (Figure S54). Compound **7** was named colletamide A.

Using the same structural elucidation process, compounds 8, 9 and 10 were identified as (2*E*,8*E*)-*N*-[(*R*)-1-((2*S*,4*S*)-4-hydroxy-5-oxotetrahydrofuran-2-yl)-2-phenylethyl]deca-2,8-dienamide (named colletamide B), 2-[(2*E*,8*E*)-deca-2,8-dienamido]-3-(2-hydroxyphenyl)propionic acid (named Colletamide C) and 4-[(2*E*,8*E*)-deca-2,8-dienamido)]-3-hydroxy-5-phenylpentanoic acid (named colletamide D), respectively (Figure S55-S79).

The absolute configuration of **9** and **10** were assumed to be *2′R, 4′S, 5′S* and *3′ S, 4′S*, respectively, by comparison with **7** and **8** and taking into account that all the isolated compounds were most probably biosynthesized from a L-phenylalanine precursor.

Finally, all isolated compounds were tested for their potential cytotoxicity on MRC-5 cells lines. The cytotoxic activities of cyclopeptides **1** to **3** were confirmed, with IC_50_ between 17.7 and 1.40 µM (Table S6). Cytotoxic activity of cytochalasin C and D was 3.01 and 1.97 µM, respectively.

Colletamide A-D did not exhibit any cytotoxicity (IC_50_ > 100 µM). This novel series of decadienamides was therefore not responsible for the activity of OCU *Colletotrichum* sp.2 extract, in agreement with the loss of activity of the second EtOAc extract of BSNB-0583 (OCU *Colletotrichum* sp.2). Indeed, second extract (BSNB-0583) exhibited 63 ± 4% of viability on MRC-5 cells (10 µg/mL), therefore showing a reduced cytotoxicity compared to the initial extract (32 ± 1%). Variation in metabolite production is a common phenomenon already described in the literature^1^. Comparison of the LC–MS profiles between the cytotoxic and the non-cytotoxic extract shows a difference in intensity for the ion at *m/z* 346.2738. This ion belongs to a cluster of nodes specific to BSNB-0583 strain in the MN and this could be responsible for the altered biological activity of the extract. Curiously, the metabolite at *m/z* 346.2738 has not been recovered from any fractions after the purification process suggesting that it could be sensitive to purification conditions. Nevertheless, the chemical formula C_22_H_35_NO_2_^+^ could be attributed to its protonated species [M + H]^+^ (calculated *m/z* 346.2746, mass error − 2.3 ppm). Moreover, MS/MS spectrum of the precursor ion at *m/z* 346.2738 shows a fragment ion at *m/z* 107.0497 (C_7_H_7_O) and complementary fragment ion at *m/z* 240.2327. This typical pattern has been previously described for molecules bearing a para-substituted phenol moiety such as vitroprocine derivatives, for example^34^.

Whereas ergosterol, colletotrichin, flavonol, cyclohexene and azaphilone derivatives are known secondary metabolites of *Colletotrichum*, the isolated cyclopeptides **1**, **2** and **3** have not been reported before in such strains of this genus^35–39^. Colutellin A, an antimycotic peptide has previously been isolated from *Colletotrichum dematium*^40^. The presence of such peptides can explain the cytotoxic activity of the strain BSNB-0580. Indeed, cyclopeptides isolated from microorganisms and showing toxic activities have been extensively described in the literature^41^.

The cytotoxic activity of cytochalasins and their mechanisms of action have been intensely studied. They are known to inhibit the polymerization of actin and disrupt cellular morphology and cell division^42^. More specifically, cytochalasin D inhibits protein synthesis in HeLA cells^43^, and cytochalasin E is a potent antiangiogenic agent due to the presence of an epoxide^44^. As far as we know, it is the first time that cytochalasins have been isolated from *Colletotrichum* species. Previous studies report their presence in the genera *Xylaria*^45,46^, *Diaporthe*/*Phomopsis*^47^, *Aspergillus*^48^, *Phoma*^49^. At last, hirsutatin A has been first isolated from an entomopathogen fungus *Hirsutella nivea* and showed no cytotoxic activity against Vero cell lines^50^.

Compound **7** had been already isolated from the fungal strains *Pseudoarachniotus roseus*, including a patent for antihypercholesterol and antimicrobial activities^51^, and from *Chaetomium cupreum*^52^. The arenosin, an another analogues has also previously been isolated *P. roseus*. The chaetocuprum and the 10,11-*epoxy*chaetocuprum have been obtained from the endophyte fungus *C. cupreum*^53^. Finally, other lipoamino acids with fatty acids derivatives of phenylalanine (similar to compound **9**) have already been isolated from bacteria of the *Pantoea* genus^54^. All of these compounds are known to have antibacterial activity but no cytotoxicity has been reported in the literature.

## Conclusion

The newly generated collection of 42 endophytic *Colletotrichum* strains has been chemically investigated by up-to-date analytical tools, i.e. MALDI-TOF profiling and molecular networking dereplication, to identify secondary cytotoxic metabolites. We found that *Colletotrichum* strains produce specific specialized metabolites depending on the Operational Chemically Unit OCU highlighted by the MALDI-TOF MS clustering. This has finally led to the isolation and complete de novo characterisation of 10 *Colletotrichum* specialised metabolites.

Cytochalasins and peptides have been isolated in three cytotoxic extracts (BSNB-0615, -0652 and -0580), but chemical diversity remains unknown for the 8 remaining cytotoxic extracts. At this point, this lack of information can be due to incomplete MS/MS databases or to the fact that extracts are actually composed of currently unknown specialized metabolites.

According to t-SNE representation of the MS/MS data allowing to evaluate inter-cluster distance, we demonstrate that OCU *Colletotrichum* sp. 4 is producing metabolites related to those in *Colletotrichum* sp.11, especially cytochalasin analogues. Indeed, the chemical study of one strain of OCU *Colletotrichum* sp.4 (BSNB-0536) has led to the isolation of cytochalasin metabolites.

Finally, OCU *Colletotrichum* sp.2 (BSNB-0583) was found to be the strain with the highest probability to isolate new and bioactive metabolites. Chemical study of the extract lead to the isolation of a new series of metabolites containing a decadienamide chain. These metabolites are named colletamide A-D and seems to have, has their known analogues, antimicrobial activities. They are the major components of the extract but minor ones also exist in this series, according to the MN.

The methodology presented in this study targets promising bioactive strains: we distinguished different Operational Chemically Unit of *Colletotrichum* by MALDI-TOF MS fingerprinting of genetically close isolates and observed their chemical diversity by Molecular Networking. Application of this approach for investigating many strains exhibiting various biological activities could accelerate the discovery of novel bioactive metabolites.

## Experimental section

### General experimental procedures

NMR spectra were recorded in CD_3_OD or (CD_3_)_2_SO on a Bruker 500 MHz spectrometer or a Bruker 600 MHz spectrometer equipped with a 2 mm invers detection probe. Chemical shifts (*δ*) are reported in ppm based on TMS signal. Coupling constants (J) are in hertz. High-resolution ESI-TOF–MS measurements were performed using a Waters Acquity UPLC system with column bypass coupled to a Waters Micromass LCT Premier time-of-flight mass spectrometer equipped with an electrospray interface (ESI). Flash chromatography was performed on a Grace Reveleris system equipped with a 120 g C_18_ column or a 40 g C_18_ column^55^. Flow rate was 80 mL/min or 40 mL/min respectively and detection was performed with dual UV at 210 and 270 nm and ELSD. Analytical and preparative HPLCs were conducted on a Gilson system equipped with a 322 pumping device, a GX-271 fraction collector, a 171 diode array detector and a prepELSII detector electrospray nebulizer. Wavelenghts for UV absorbance were 210, 220, 254, 270 and 320 nm. Columns used for analytical and preparative experiments included a Phenomenex Luna C_18_ 5 µm 4.6 × 250 mm and a Phenomenex Luna C_18_ 5 µm 21.2 × 250 mm. Flow rate was 1 mL/min and 21 mL/min respectively for analytical and preparative experiments. All solvents were HPLC grade and HPLC-grade water was obtained with a Milli-Q water purification system (Synergy, Merck). For UHPLC-HRMS analysis, chromatographic separation was performed on an Acquity UHPLC system interfaced to a Q-Exactive Plus mass spectrometer (Thermo Scientific, Bremen, Germany) with a Waters BEH C18 50 × 2.1 mm × 1.7 μm analytical column, using a heated electrospray ionization (HESI-II) source. Detection was made by an Acquity UPLC photodiode array detector from 200 to 500 nm. The optimized HESI-II parameters were as follows: source voltage, 3.5 kV (pos); sheath gas flow rate (N2), 55 units; auxiliary gas flow rate, 15 units; spare gas flow rate, 3.0; capillary temperature, 275.00 °C (pos), S-Lens RF Level, 45. The data-dependent MS/MS events were performed on the four most intense ions detected in full scan MS (Top3 experiment). The MS/MS isolation window width was 1 Da, and the normalized collision energy (NCE) was set to 35 units. In data-dependent MS/MS experiments, full scans were acquired at a resolution of 35 000 FWHM (at m/z 200) and MS/MS scans at 17 500 FWHM both with a maximum injection time of 50 ms. After being acquired in a MS/MS scan, parent ions were placed in a dynamic exclusion list for 2.0 s.

MALDI-TOF MS analyses were performed using an UltrafleXtreme mass spectrometer (Bruker Daltonics, Bremen). Acquisitions were performed in linear positive ion mode. The laser intensity was set just above the ion generation threshold to obtain peaks with the highest possible signal-to-noise (S/N) ratio without significant peak broadening. The mass spectrometer was externally calibrated using a mixture of proteins (Insulin, Cytochrome C, Myoglobin and Ubiquitin I). All data were processed using the program FlexAnalysis (Bruker Daltonics, Bremen).

### Isolation and purification of endophytic strains

*Astrocaryum sciophilum* palm trees were sampled in French Guiana at Piste de Saint-Elie, Sinnamary, in July 2014. The general procedures adopted for isolation of the microorganisms followed the methodology described by Casella et al*.*^56^. After collection, the plant material was washed with sterile water and surface sterilised by sequential immersion in 70% aqueous ethanol (3 min), followed by 5% aqueous sodium hypochlorite (5 min) and finally by 70% aqueous ethanol (1 min). Leaves were cut into small pieces (1–0.5 cm^2^) which were placed on Potato Dextrose Agar medium (PDA, Fluka Analytical, Germany) in Petri dishes at 28 °C (4–5 parts per Petri dishes). Each individual hyphal tip of emerging fungi was removed and placed on a sterile PDA culture medium in 10 cm Petri dishes. The leaf fragments were cultured for a maximum of one month. All isolated endophytic strains have been deposited at the ICSN/CNRS Strain library France. Strains are maintained in triplicate in 2 ml Eppendorf tubes containing 1 ml of a solution of glycerol and water (1:1) at − 80 °C.

### Phylogenetic analyses

Fungal strains were identified using nucleotide sequencing of the rDNA ITS region (ITS1-5,8S-ITS2). ITS sequences were blasted on NCBI GenBank (accessed 2018-03-12) to search for closest strains (See supplementary Table S2). 16 sequences from closest representatives species available in the GenBank database were retrieved for comparison (*Colletotrichum gloeosporioides* (MH864569), *Colletotrichum* sp. strain LGMF1580 (MG976389), *C. gloeosporioides* (MH865232), *C. gloeosporioides* (MH866036), *C. fragariae* (MH864138), *C. vietnamense* (MH863700), *Colletotrichum* sp. strain AHGB10 (MH267867), *C. gloeosporioides* (KT004429), *C. gloeosporioides* (KR995714), *Colletotrichum* sp. strain AHGB15 (MH267881), *C. horii* (KR995727), *C. horii* (LC186042), *C. karstii* (MG602059), *C. fructicola* (MH865616), *C. siamense* (MG807423), *C. queenslandicum* (JX010184)). *Monilochaetes infuscans* (JQ005780) was used for the output group. Sequences were aligned in MAFFT version 7 (https://mafft.cbrc.jp/alignment/server/)^57^. The resulting alignment was saved into Phylip4 format. A phylogenetic tree was constructed by maximum likelihood (ML) inference on the CIPRES server (https://www.phylo.org)^58^. The ML analysis was performed using RaxML-HPC2 on XSEDE (8.2.10). Branch support was evaluated by a bootstrapping method with 1000 replicates. The final phylogenetic tree was visualized using FigTree version 1.4.4. The sequence data have been submitted to GenBank with an accession number for each strain (See Supplementary Table S1).

### MALDI-TOF analysis

Protein extraction were performed in triplicate according to the acetonitrile/formic acid extraction procedure of Bruker MALDI Biotyper^20^. After a culture of 72 h at 28 °C on PDA (Potato Dextrose Agar) medium, 10 mg of each isolate was suspended in 300 µL of water and 900 µL of 100% ethanol then centrifuged at 13,000 rpm. The supernatant is discarded and the pellet was resuspended in 40 µL of formic acid and vortexed. An equal volume of 100% acetonitrile was then added and the solution mixed. After centrifugation at 13,000 rpm, the supernatant was stocked at − 20 °C for maximum one week and used for MALDI measurement. For MALDI analysis, 1 µL of protein extract is spotted on the sample target, directly followed by 1 µL of α-cyano-4-hydroxycinnamic acid (CHCA) matrix (50 mg/mL in CH_3_CN/H_2_O (50/50), acidified with 0.1% trifluoroacetic acid).

### Hierarchical cluster tree

A hierarchical cluster tree was constructed using the Mass-up software^59^. Each MALDI TOF MS spectra was preprocessed using the following parameters: intensity was square-root transformed, smoothing was done using moving average and baseline correction using TopHat. Peak detection was set to Mass SpecWavelet with a minimum peak intensity chosen in order to detect only the major peak. Peak matching on peak lists was done with a tolerance of 700 ppm for intra-sample matching. Clustering analysis was generated using Jaccard distance function and conversion values of presence.

### Cultures and extraction of secondary metabolites

Each strain was cultivated at 28 °C in 10 Petri dishes (10 cm diameter) of PDA (Potato Dextrose Agar) media. Then, culture media was extracted with ethyl acetate (EtOAc) at room temperature during 24 h. The organic phase was removed via filtration, washed three times with H_2_O, dried with anhydrous solid Na_2_SO_4_ and evaporated using a rotary evaporator under reduced pressure to yield a crude mixture.

### UPLC-MS/MS and data analysis

Each EtOAc extract of *Colletotrichum* strain was profile on a UPLC-MS/MS to acquire mass data for the implementation of molecular network^55^. Samples were pre-treated by Solid phase extraction using Discovery® SPE 96 well plate (bed wt 100 mg/well) and plate prep vacuum Manifold. Each extract was then dissolved in 500 µL of 5% milliQ water in HPLC methanol and loaded on the cartridge bed. Elutes were injected at a concentration of 4 mg/mL for Orbitrap analysis. For UHPLC-HRMS analysis, chromatographic separation was performed on an Acquity UHPLC system interfaced to a Q- Exactive Plus mass spectrometer (Thermo Scientific, Bremen, Germany), using a heated electrospray ionization (HESI-II) source. Thermo Scientific Xcalibur 2.1 software was used for instrument control and data analysis.

One µL aliquot of each sample was injected and eluted at 0.6 mL/min. The linear gradient between CH_3_CN and H_2_O (0.1% formic acid modifier) was made from 5 to 100% of acetonitrile over 7 min following by an isocratic gradient of 100% CH_3_CN for 1 min. In positive ion mode, the di-isooctyl phthalate C_24_H_38_O_4_ [M + H]^+^ ion (*m/z* 391.28429) was used as an internal lock mass. The mass analyzer was calibrated using a mixture of caffeine, methionine−arginine−phenylalanine−alanine−acetate (MRFA), sodium dodecyl sulfate, sodium taurocholate, and Ultramark 1621 in an acetonitrile/methanol/water solution containing 1% formic acid by direct injection.

The optimized HESI-II parameters were as follows: source voltage, 3.5 kV (pos); sheath gas flow rate (N2), 55 units; auxiliary gas flow rate, 15 units; spare gas flow rate, 3.0; capillary temperature, 275.00 °C (pos), S-Lens RF Level, 45. The mass analyzer was calibrated using a mixture of caffeine, methionine−arginine−phenylalanine−alanine−acetate (MRFA), sodium dodecyl sulfate, sodium taurocholate, and Ultramark 1621 in an acetonitrile/ methanol/water solution containing 1% formic acid by direct injection. The data-dependent MS/MS events were performed on the four most intense ions detected in full scan MS (Top3 experiment). The MS/MS isolation window width was 1 Da, and the normalized collision energy (NCE) was set to 35 units. In data-dependent MS/MS experiments, full scans were acquired at a resolution of 35 000 FWHM (at m/z 200) and MS/MS scans at 17 500 FWHM both with a maximum injection time of 50 ms. After being acquired in a MS/MS scan, parent ions were placed in a dynamic exclusion list for 2.0 s.

### MZmine 2 data-preprocessing parameters

The complete procedure has been Raw files were converted into mzXML files using MSConvert software. Then mzXML files were processed using MZmine 2.37^60,61^. Mass detection was realized with centroid mass detector with the noise level set to 1.0E5 for MS level set to all. The ADAP chromatogram builder^62^ was achieved using a minimum group size of scans of 5, minimum group intensity threshold of 1.0E5, minimum highest intensity of 1.0E5 and *m/z* tolerance of 0.002 or 5 ppm. Wavelets (ADAP) algorithm was used for chromatogram deconvolution with the following settings: S/N threshold of 10, intensity window SN, minimum feature height of 1000, coefficient area threshold of 100, peak duration range between 0.01 and 0.5 and the RT wavelet range between 0.001 and 0.05. The *m/z* and RT range for MS/MS scan pairing was set to 0.001 Da and 0.05 min respectively. Chromatograms were deisotoped using the isotopic peaks grouper algorithm with a *m/z* tolerance of 0.003 (5 ppm), a RT tolerance of 0.1 (absolute), a maximum charge of 2 and the representative isotope used was the most intense. Peak alignment was performed using the join aligner method: *m/z* tolerance of 0.001 or 5.0 ppm, weight for *m/z* of 0.001, RT tolerance of 0.3 min, weight for RT of 0.1. Adduct search (Na^+^, K^+^, NH_4_^+^, CH_4_CN^+^) was conducted on the peak list with a RT tolerance set to 1.0 min and the maximum relative peak height at 50%. Adducts found were then removed from the peak list. Peak list was gap-filled with the peak finder module: intensity tolerance of 90%, *m/z* tolerance of 0.001 or 5.0 ppm and RT tolerance of 0.1 min. Peak list was exported for GNPS to create a mgf file (https://doi.org/10.5281/zenodo.3862573). Row ID, row *m/z*, row retention time and peak area was exported in an associated CSV file. After a first analysis of the generated MN, this peak list was reduced to the ions with *m/z* between 200 and 900 to diminish the data size.

### Molecular network analysis

After the preprocessing of the LC–MS/MS data with MZmine 2.37, the output mgf file was then processed with MetGem software^28^ to give a network containing 15,261 nodes. Molecular network was generated using the following parameters: *m/z* tolerance set to 0.02, Minimum Matched Peaks set to 6, topK set to 10, Minimal Cosine score Value of 0.7 and Max. Connected Component Size of 100. Associated CSV file was then loaded. For the mapping process, relative quantification of each ion was represented as pie chart-diagrams, whose proportions were based on respective areas of the corresponding extracted ion chromatograph area (EIC). The spectra in the network were then searched for analogues against the spectral libraries available. The library spectra were filtered in the same manner as the input data. All matches kept between network spectra and library spectra were required to have a score above 0.7 and at least 6 matched peaks. *m/z* Tolerance for the search of analogues was set to 100. For t-SNE output, cosine score threshold (0.7) was similar to molecular network view. The number of iterations, perplexity, learning-rate and early exaggeration parameters were set to 1000, 6, 200 and 12, respectively. These values were previously optimized on another complex set of data^28^.

For a more advanced retreatment of the network, MN was also exported on Cytoscape 3.7.0 software^63^.

### Large-scale cultivation and isolation

Strains were cultivated during 15 days at 28 °C in 14 cm Petri dishes of PDA (Potato Dextrose Agar) media. Each strain was extracted three times consecutively with ethyl acetate (EtOAc) at room temperature. The combined organic solution was washed as previously.

#### Strain BSNB-0580

The large-scale cultivation of *Colletotrichum* sp. BSNB-0580 was conducted on 226 Petri dishes (diameter 14 cm) to yield 4.7 g of a brown crude extract. 4.5 g of this crude extract was fractionated by reverse flash chromatography on C_18_ column with a 5-min-step gradient of water mixed with an increasing proportion of acetonitrile (v/v, 80:20–50:50–35:65–20:80–0:100, 0.1% formic acid modifier). 6 fractions were generated based on the UV and ELSD detection and after spotting on TLC plates (in various mixture of acetonitrile and H_2_O): F1 (158.7 mg, 3.5%), F2 (1.29 g, 28.7%), F3 (120.1 mg, 2.7%), F4 (531.6 mg, 11.8%), F5 (71.3 mg, 1.6%), F6 (144.5 mg, 3.2%). A step gradient of acetonitrile–methylene chloride (v/v, 50:50–0:100) was conducted to generate 4 additional fractions based on the spot on TLC plates: F7 (271.9 mg, 6.0%), F8 (105.2 mg, 2.3%), F9 (381.9 mg, 8.5%) and F10 (114.7 mg, 2.5%).

F4 was selected for purification based on its cytotoxic activity and its HPLC profile. F4 was purified by preparative HPLC (isocratic elution 65% CH_3_CN/H_2_O 0.1% formic acid modifier for a duration of 15 min per run) to obtain 3 cyclopeptides in sufficient amount: cyclo-(Phe-Leu-Leu-Leu-Val) **(1)** (3.1 mg, t_R_ = 9.4 min), cyclo-(Phe-Leu-Leu-Leu-Leu) **(2)** (2.9 mg, t_R_ = 11.0 min), and cyclo-(Phe-Leu-Leu-Leu-Ile) **(3)** (265.5 mg, t_R_ = 12.0 min). The chromatogram was monitored by UV absorbance.

#### Strain BSNB-0536

The large-scale cultivation of *Colletotrichum* sp. BSNB-0536 was conducted on 220 Petri dishes (diameter 14 cm) to yield 5.4 g of a brown crude extract. 5.2 g of this crude extract was fractionated by reverse flash chromatography on C_18_ column with a 5-min-step gradient of water mixed with an increasing proportion of acetonitrile (v/v, 95:5–80:20–70:30–50:50–30:70–20:80–10:90–0:100, 0.1% formic acid modifier). 6 fractions were generated based on the UV and ELSD detection: F1 (32.8 mg, 0.6%), F2 (4.0 g, 76.9%), F3 (329.7 mg, 6.3%), F4 (49.9 mg, 1.0%), F5 (128.0 mg, 2.5%), F6 (28.0 mg, 0.5%). A step gradient of acetonitrile–methylene chloride (v/v, 50:50–0:100) was conducted to generate 2 additional fractions: F7 (271.1 mg, 5.2%) and F8 (152.6 mg, 2.9%).

BSNB-0536-F2 was then again fractionated by reverse flash chromatography on C_18_ column with a 5-min-step gradient of water mixed with an increasing proportion of acetonitrile (v/v, 80:20–65:35–50:50–35:65–20:80–10:90–0:100, 0.1% formic acid modifier). 13 fractions were generated based on the UV and ELSD detection: F2-A (99.3 mg, 5.0%), F2-B (297.5 mg, 14.9%), F2-C (47.5 mg, 2.4%), F2-D (92.5 mg, 4.6%), F2-E (133.6 mg, 6.7%), F2-F (185.4 mg, 9.3%), F2-G (160.4 mg, 8.0%), F2-H (319.8 mg, 16.0%), F2-I (194.9 mg, 9.7%), F2-J (216.6 mg, 10.8%), F2-K (312.1 mg, 15.6%), F2-L (224.2 mg, 11.2%) et F2-M (221.0 mg, 11.0%). A step gradient of acetonitrile–methylene chloride (v/v, 50:50–0:100) was conducted to generate 2 additional fractions: F2-N (74.7 mg, 3.7%) and F2-O (2.7 mg, 0.1%).

138.2 mg of fraction BSNB-0536-F2-J was further purified by reverse flash chromatography on a 40 g C_18_ column with a linear gradient between water and acetonitrile (both solvent contained 0.1% formic acid): 60:40 for 5 min, 0:100 in 20 min, 0:100 for 5 min. Three molecules were generated based on the UV and ELSD detection: Cytochalasin D **(4)** (40.4 mg, 29.2%, t_R_ = 7.0 min), Cytochalasin C **(5)** (2.9 mg, 2.1%, t_R_ = 10.2 min) and Hirsutatin A **(6)** (21.5 mg, 15.6%, t_R_ = 12.8 min).

#### Strain BSNB-0583

The large-scale cultivation of *Colletotrichum* sp. BSNB-0583 was conducted on 232 Petri dishes (diameter 14 cm) to yield 1.5 g of a yellow crude extract. 1.4 g of this crude extract was fractionated by reverse flash chromatography on C_18_ column with a 5-min-step gradient of water mixed with an increasing proportion of acetonitrile (v/v, 95:5–80:20–65:35–50:50–35:65–20:80–10:90–0:100, 0.1% formic acid modifier). 12 fractions were generated based on the UV and ELSD detection: F1 (16.7 mg, 1.2%), F2 (31.7 mg, 2.3%), F3 (74.7 mg, 5.3%), F4 (96.5 mg, 6.9%), F5 (29.8 mg, 2.1%), F6 (42.6 mg, 3.0%), F7 (35.9 mg, 2.6%), F8 (27.3 mg, 2.0%), F9 (64.0 mg, 4.6%), F10 (16.7 mg, 1.2%), F11 (161.0 mg, 11.5%) and F12 (7.6 mg, 0.5%). A step gradient of acetonitrile–methylene chloride (v/v, 50:50–0:100) was conducted to generate 2 additional fractions: F13 (430.4 mg, 30.7%) and F14 (172.1 mg, 12.3%).

BSNB-0583-F6 and BSNB-0583-F5 were purified by preparative HPLC (isocratic elution 55% CH3CN/H2O 0.1% formic acid modifier and isocratic elution 50% CH_3_CN/H_2_O 0.1% formic acid for a duration of 25 min per run). Chromatograms were monitored by UV absorbance. From F6, two molecules were isolated: Colletamide A **(7)** (1.7 mg, t_R_ = 12.0 min) and Colletamide B **(8)** (3.5 mg, t_R_ = 13.8 min). From F5, two molecules were isolated: Colletamide C **(9)** (1.5 mg, t_R_ = 12.8 min) and Colletamide D **(10)** (0.6 mg, t_R_ = 14.5 min).

To isolate the metabolite that give the ion of *m/z* 346.275, a second large-scale cultivation of BSNB-0583 was performed on 169 Petri dishes. 1.9 g of crude extract was obtained and a liquid–liquid partition (MeOH/Hexane) on 0.8 g was executed. 0.5 g recovered from the MeOH phase was futher fractionated by reverse flash chromatography on C_18_ column (12 g, flow rate 30 mL/min). For the elution, a linear gradient was performed between water and acetonitrile modified with 031% formic acid: 25/75 for 3 min, 25/75 to 0/100 in 10 min and 0/100 for 6 min. Thanks to the ELSD detection, nine zones were collected and analysed with mass spectrometry. The fraction 6 (1.7 mg), contained the targeted mass of *m/z* 346.275.

Cyclo-(Phe-Leu-Leu-Leu-Val) **(1)**: White powder ; ^1^H-NMR (500 MHz, (CD_3_)_2_SO) *δ* Phe 8.33 (1H, s, NH), 7.22 (2H, m, H-5), 7.17 (2H, m, H-6), 7.16 (1H, m, H-7), 4.53 (1H, dd, *J* = 15.2, 7.6, H-2), 3.00 (1H, dd, *J* = 13.2, 7.6, H-3), 2.69 (1H, dd, *J* = 13.2, 7.3, H-3); Leu1 8.48 (1H, d, *J* = 7.9, NH), 4.26 (1H, dd, *J* = 15.2, 7.4, H-2), 1.41 (1H, m, H-3), 1.28 (1H, m, H-3), 1.23 (1H, m, H-4), 0.79 (3H, m, H-5), 0.76 (3H, m, H-6); Leu2 8.60 (1H, s, NH), 4.20 (1H, m, H-2), 1.54 (1H, m, H-4), 1.49 (2H, m, H-3), 0.87 (3H, t, *J* = 6.5, H-5), 0.79 (3H, m, H-6); Leu3 8.40 (1H, s, NH), 4.33 (1H, dd, *J* = 16.3, 8.0, H-2), 1.49 (2H, m, H-3), 1.45 (1H, m, H-4), 0.87 (3H, t, *J* = 6.5, H-5), 0.85 (3H, d, *J* = 6.2, H-6); Val 8.88 (1H, s, NH), 3.58 (1H, m, H-2), 2.13 (1H, m, H-3), 0.79 (3H, m, H-4), 0.67 (3H, d, J = 6.7, H-5). ^13^C-NMR ((CD_3_)_2_SO, 600 MHz) *δ* Phe 170.4 (C-1), 137.8 (C-4), 129.1 (C-5), 128.0 (C-6), 126.1 (C-7), 53.4 (C-2), 37.5 (C-3); Leu1 170.9 (C-1), 51.0 (C-2), 39.4 (C-3), 24.1 (C-4), 22.6 (C-5), 22.4 (C-6); Leu2 171.5 (C-1), 52.7 (C-2), 40.6 (C-3), 24.4 (C-4), 22.9 (C-5), 21.2 (C-6); Leu3 171.1 (C-1), 52.6 (C-2), 41.1 (C-3), 24.6 (C-4), 22.4 (C-5), 22.3 (C-6); Val 171.0 (C-1), 62.5 (C-2), 29.4 (C-3), 19.2 (C-4, C-5). ESI-HRMS 586.3962 ([M + H]^+^, C_32_H_52_N_5_O_5_^+^; calc. 586.3963, err. − 0.17 ppm), 1171.7892 ([2 M + H]^+^, C_64_H_103_N_10_O_10_^+^; calc. 1171.7892, err. − 0.00 ppm).

Cyclo-(Phe-Leu-Leu-Leu-Leu) **(2)**: White powder ; ^1^H-NMR (500 MHz, (CD_3_)_2_SO) *δ* Phe 8.34 (1H, s, NH), 7.22 (2H, m, H-5), 7.17 (2H, m, H-6), 7.16 (1H, m, H-7), 4.48 (1H, dd, *J* = 15.5, 8.0, H-2), 3.02 (1H, dd, *J* = 13.2, 7.6, H-3), 2.70 (1H, dd, *J* = 7.0, 13.2, H-3); Leu1 8.34 (1H, d, *J* = 8.2, NH), 4.25 (1H, m, H-2), 1.41 (1H, m, H-3), 1.29 (1H, m, H-3), 1.23 (1H, m, H-4), 0.78 (3H, m, H-5), 0.76 (3H, d, *J* = 6.5, H-6); Leu2 8.46 (1H, m, NH), 4.21 (1H, m, H-2), 1.52 (2H, m, H-3), 1.44 (1H, m, H-4), 0.87 (3H, m, H-5), 0.84 (3H, m, H-6); Leu3 8.50 (1H, s, NH), 4.30 (1H, m, H-2), 1.46 (2H, m, H-3), 1.41 (1H, m, H-4), 0.87 (3H, m, H-5), 0.79 (3H, m, H-6) ; Leu4 8.94 (1H, s, NH), 4.07 (1H, m, H-2), 1.47 (1H, m, H-3), 1.44 (1H, m, H-4), 1.36 (1H, m, H-3), 0.83 (3H, m, H-5), 0.77 (3H, m, H-6). ^13^C-NMR ((CD_3_)_2_SO, 600 MHz) *δ* Phe 170.4 (C-1), 137.9 (C-4), 129.3 (CH, C-5), 128.0 (CH, C-6), 126.1 (C-7), 53.5 (C-2), 37.3 (C-3); Leu1 171.6 (C-1), 50.8 (C-2), 39.4 (C-3), 24.1 (C-4), 22.8 (C-5), 22.3 (C-6); Leu2 170.9 (C-1), 52.8 (C-2), 40.7 (C-3), 24.6 (C-4), 22.8 (C-5), 22.2 (C-6); Leu3 171.1 (C-1), 52.6 (C-2), 40.6 (C-3), 24.5 (C-4), 23.0 (C-5), 21.3 (C-6); Leu4 172.0 (C-1), 53.9 (C-2), 40.5 (C-3), 24.6 (C-4), 22.3 (C-6), 21.8 (C-5). ESI-HRMS 600.4115 ([M + H]^+^, C_33_H_54_N_5_O_5_^+^; calc. 600.4119, err. − 0.66 ppm), 1199.8195 ([2 M + H]^+^, C_66_H_107_N_10_O_10_^+^; calc. 1199.8166, err. 2.41 ppm).

Cyclo-(Phe-Leu-Leu-Leu-Ile) **(3)**: White powder ; ^1^H-NMR (500 MHz, (CD_3_)_2_SO) *δ* Phe 7.99 (1H, d, *J* = 8.9, NH), 7.24 (2H, m, H-5), 7.18 (2H, m, H-6), 7.17 (1H, m, H-7), 4.65 (1H, dd, *J* = 15.3, 8.5, H-2), 2.91 (1H, dd, *J* = 13.6, 6.4, H-3), 2.72 (1H, dd, *J* = 13.5, 8.6, H-3); Leu1 8.56 (1H, d, *J* = 8.0, NH), 4.17 (1H, dd, *J* = 14.4, 7.2, H-2), 1.42 (1H, m, H-3), 1.34 (1H, m, H-3), 1.31 (1H, m, H-4), 0.91 (3H, d, J = 5.8, H-5), 0.84 (3H, d, *J* = 5.9, H-6); Leu2 7.32 (1H, d, *J* = 5.5, NH), 4.10 (1H, m, H-2), 1.60 (1H, m, H-4), 1.52 (2H, m, H-3), 0.87 (3H, d, *J* = 6.3, H-5), 0.78 (3H, m, H-6); Leu3 8.63 (1H, d, *J* = 7.0, NH), 4.32 (1H, dd, *J* = 15.5, 7.4, H-2), 1.52 (1H, m, H-3), 1.46 (2H, m, H-3, H-4), 0.87 (3H, d, J = 6.3, H-5), 0.78 (3H, m, H-6); Ile 8.42 (1H, d, J = 7.0, NH), 3.30 (1H, m, H-2), 2.23 (1H, m, H-4), 1.39 (1H, m, H-4), 1.01 (1H, sept, *J* = 7.3, H-3), 0.77 (3H, m, H-6), 0.62 (3H, d, *J* = 6.7, H-5) ; ^13^C-NMR ((CD_3_)_2_SO, 500 MHz) *δ* Phe 171.6 (C-1), 137.3 (C-4), 129.1 (C-5), 127.9 (C-6), 126.2 (C-7), 52.8 (C-2), 38.4 (C-3); Leu1 171.5 (C-1), 51.8 (C-2), 38.7 (C-3), 24.0 (C-4), 22.2 (C-5), 22.4 (C-6); Leu2 171.5 (C-1), 52.2 (C-2), 40.0 (C-3), 24.3 (C-4), 23.0 (C-5), 20.7 (C-6); Leu3 171.0 (C-1), 51.9 (C-2), 40.3 (C-3), 24.6 (C-4), 22.6 (C-5), 22.2 (C-6); Ile 171.1 (C-1), 62.7 (C-2), 33.2 (C-3), 25.1 (C-4), 15.1 (C-6), 9.8 (C-5); ESI-HRMS 600.4110 ([M + H]^+^, C_33_H_54_N_5_O_5_^+^; calc. 600.4119, err. − 1.49 ppm), 1199.8179 ([2 M + H]^+^, C_66_H_107_N_10_O_10_^+^; calc. 1199.8166, err. − 1.28 ppm).

Cytochalasin D **(4)**: White powder.[α]_D_^20^ –40 (c 0.33 g/100 mL, CHCl_3_). ^1^H-NMR (500 MHz, CD_3_OD) *δ* 7.30 (2H, m, H-3′), 7.23 (1H, m, H-4′), 7.18 (2H, m, H-2′), 5.98 (1H, dd, *J* = 15.9, 2.6, H-20), 5.55 (1H, dd, *J* = 15.9, 10.0, H-13), 5.42 (1H, t, *J* = 2.5, H-21), 5.27 (1H, m, H-14), 5.24 (1H, dd, *J* = 15.9, 2.4, H-19), 5.17 (1H, s, H-12), 4.98 (1H, s, H-12), 3.75 (1H, d, *J* = 10.2, H-7), 3.28 (1H, m, H-3), 2.86 (1H, m, H-10), 2.83 (1H, m, H-8), 2.81 (1H, m, H-16), 2.71 (1H, dd, *J* = 13.3, 7.8, H-10), 2.62 (1H, m, H-5), 2.37 (1H, m, H-15), 2.29 (3H, s, H-25), 2.17 (1H, dd, *J* = 4.0, 2.6, H-4), 2.00 (1H, m, H-15), 1.48 (3H, s, H-23), 1.14 (3H, d, *J* = 6.8, H-22), 0.56 (3H, d, *J* = 6.8, H-11). ^13^C-NMR (500 MHz, CD_3_OD) *δ* 211.7 (C17), 176.5 (C1), 171.8 (C24), 150.9 (C6), 138.4 (C1′), 134.4 (C14), 133.4 (C20), 131.8 (C13), 130.9 (C2′, C6′), 129.6 (C3′, C5′), 128.9 (C19), 127.9 (C4′), 113.7 (C12), 79.3 (C9), 78.2 (C21), 72.3 (C7), 54.9 (C3), 49.7 (C4), 47.8 (C8), 44.9 (C10), 43.4 (C16), 39.4 (C15), 33.4 (C5), 24.6 (C23), 20.6 (C25), 19.7 (C22), 13.5 (C11). ESI-HRMS: 508.2717 ([M + H]^+^, C_30_H_38_NO_6_^+^; calc. 508.2694, err. − 4.52 ppm), 530.2538 ([M + Na]^+^, C_30_H_37_NO_6_Na^+^; calc. 530.2513, err. 4.71 ppm).

Cytochalasin C **(5)**: White powder.[α]_D_^20^ -16 (c 0.33 g/100 mL, CHCl_3_). ^1^H-NMR (500 MHz, CD_3_OD) *δ* 7.31 (2H, m, H-3′, H-5′), 7.24 (1H, m, H-4′), 7.23 (2H, m, H-2′, H-6′), 5.95 (1H, dd, *J* = 15.7, 2.3, H-20), 5.87 (1H, t, *J* = 2.2, H-21), 5.72 (1H, dd, *J* = 10.2, 15.7, H-13), 5.28 (1H, dd, *J* = 15.8, 2.3, H-19), 5.26 (1H, m, H-14), 3.72 (1H, d, *J* = 9.3, H-7), 3.31 (1H, m, H-3), 3.03 (1H, dd, *J* = 13.3, 5.2, H-10), 2.87 (1H, dd, *J* = 13.3, 9.9, H-10), 2.83 (1H, m, H-16), 2.47 (2H, m, H-4, H-9), 2.40 (1H, q, *J* = 11, H-15), 2.30 (3H, s, H-25), 2.01 (1H, m, H-15), 1.49 (3H, s, H-11), 1.60 (3H, s, H-23), 1.14 (3H, d, *J* = 6.8, H-22), 0.4 (3H, s, H-11). ^13^C-NMR (600 MHz, CD_3_OD) *δ* 211.6 (C17), 176.9 (C1), 172.0 (C24), 139.0 (C1′), 134.2 (C14), 134.2 (C6), 133.1 (C20), 132.1 (C13), 130.7 (C2′, C6′), 129.8 (C3′, C5′), 129.3 (C19), 127.9 (C4′), 127.9 (C5), 79.4 (C18), 76.7 (C21), 70.1 (C7), 60.2 (C3), 54.8 (C9), 50.5 (C8), 50.3 (C4), 44.9 (C10), 43.4 (C16), 39.5 (C15), 24.6 (C23), 20.7 (C25), 19.8 (C22), 17.1 (C12), 14.5 (C11). ESI-HRMS: 508.2681 ([M + H]^+^, C_30_H_38_NO_6_^+^; calc. 508.2694, err. − 2.58 ppm), 530.2525 ([M + Na]^+^, C_30_H_37_NO_6_Na^+^; calc. 530.2513, err. 2.26 ppm).

Hirsutatin A **(6)**: Yellow solid.[α]_D_^20^—63 (c 0.2 g/100 mL, CH_3_OH). ^1^H-NMR (500 MHz, CD_3_OD) *δ* Phe 7.27 (5H, m, H-5, H-6, H-7), 5.20 (1H, dd, *J* = 8.8, 4.9, H-2), 3.05 (1H, m, H-3), 2.96 (1H, dd, *J* = 13.2, 4.9, H-3); Leu 3.61 (1H, t, *J* = 7.8, H-2), 3.07 (3H, s, N-Me), 1.73 (2H, m, H-3), 1.31 (1H, hept, *J* = 6.6, H-4), 0.93 (3H, d, J = 6.7, H-6), 0.87 (3H, d, *J* = 6.6 ,H-5); Thr 4.46 (1H, ddd, *J* = 12.9, 6.4, 1.7, H-3), 4.19 (1H, d, *J* = 1.7, H-2), 1.23 (3H, d, J = 6.4, H-4); Ser 4.83 (1H, m, H-2), 4.25 (1H, dd, *J* = 11.4, 3.3, H-3), 3.98 (1H, dd, *J* = 11.4, 2.5, H-3); 2-hydroxyisovaleric acid 4.62 (1H, d, *J* = 5.7, H-2), 2.20 (1H, oct, *J* = 6.7, H-3), 1.06 (3H, d, *J* = 6.7, H-5), 1.04 (3H, d, *J* = 6.7, H-4); 2-hydroxyisocaproic acid 5.26 (1H, dd, *J* = 9.6, 4.0, H-2), 1.73 (1H, m, H-3), 1.41 (1H, ddd, *J* = 14.0, 9.1, 4.0, H-3), 1.61 (1H, m, H-4), 0.93 (3H, d, *J* = 6.7, H-5), 0.90 (3H, d, *J* = 6.6, H-6). ^13^C-NMR (500 MHz, CD_3_OD) *δ* Phe 172.7 (C-1), 137.5 (C-4), 130.8 (C-5), 129.6 (C-6,), 128.1 (C-7), 52.3 (C-2), 39.8 (C-3); Leu 172.7 (C-1), 67.4 (C-2), 40.5 (N-Me), 38.1 (C-3), 26.0 (C-4), 23.5 (C-5), 22.3 (C-6); Thr 173.5 (C-1), 67.1 (C-3), 61.0 (C-2), 21.1 (C-4); Ser 171.7 (C-1), 63.8 (C-3), 55.3 (C-2); 2-hydroxyisovaleric acid 169.7 (C-1), 80.3 (C-2), 31.4 (C-3), 18.8 (C-4), 18.2 (C-5); 2-hydroxyisocaproic acid 171.9 (C-1), 74.7 (C-2), 42.6 (C-3), 25.5 (C-4), 23.4 (C-5), 21.8 (C-6). ESI-HRMS: 677.3723 ([M + H]^+^, C_34_H_53_N_4_O_10_^+^; calc. 677.3756, err. − 4.87 ppm), 1353.7501 ([2 M + H]^+^, C_68_H_105_N_8_O_20_^+^; calc. 1353.7440, err. 4.51 ppm).

Colletamide A **(7)**, (2*E*,8*E*)-*N*-[(1*S*,2′*R*,2*R*,4′*R*,4*R*,7*S*)-dihydro-5′,6-dioxospiro-(3,8-dioxatricyclo(5.1.0.0^2,4^)octane-5,2′-(3′*H*)-furan-4′-yl]-deca-2,8-dienamide: yellow solid; [α]_D_^20^ -3 (c 0.1 g/100 mL, CH_3_OH). ^1^H-NMR (500 MHz, CD_3_OD) *δ* 6.82 (1H, dt, *J* = 15.2, 7.1, H-3), 5.93 (1H, d, *J* = 15.3, H-2), 5.42 (2H, m, H-8, H-9), 4.34 (1H, t, *J* = 10.1, H-2′), 4.15 (1H, dd, *J* = 3.6, 1.8, H-7′), 4.00 (1H, dd, *J* = 3.9, 2.0, H-8′), 3.66 (1H, d *J* = 4.3, H-9′), 3.62 (1H, d, *J* = 4.3, H-6′), 2.83 (1H, dd, *J* = 13.7, 9.5, H-3′b), 2.53 (1H, dd, *J* = 13.7, 11.0, H-3′a), 2.22 (2H, m, H-4), 2.00 (2H, m, H-7), 1.63 (3H, d, *J* = 4.3, H-10), 1.47 (2H, m, H-5), 1.39 (2H, m, H-6). ^13^C-NMR (600 MHz, CD_3_OD) *δ* 198.9 (C-5′), 175.0 (C-1′), 168.5 (C-1), 147.5 (C-3), 132.2 (C-8), 126.0 (C-9), 123.6 (C-2), 84.6 (C-4′), 59.0 (C-7′), 57.2 (C-9′), 56.5 (C-6′), 53.8 (C-8′), 49.2 (C-2′), 34.2 (C-3′), 33.3 (C-7), 32.9 (C-4), 30.2 (C-6), 28.8 (C-5), 18.1 (C-10). ESI-HRMS: 362.1621 ([M + H]^+^, C_19_H_24_NO_6_^+^; calc. 362.1598, err. 6.35 ppm), 425.1692 ([M + ACN + Na]^+^, C_21_H_26_N_2_O_6_Na^+^; calc. 425.1683, err. 2.12 ppm), 745.2950 ([2 M + Na]^+^, C_38_H_46_N_2_O_12_Na^+^; calc. 745.2953, err. − 0.40 ppm).

Colletamide B **(8)**, (2*E*,8*E*)-*N*-[(*R*)-1-((2*S*,4*S*)-4-hydroxy-5-oxotetrahydrofuran-2-yl)-2-phenylethyl]deca-2,8-dienamide: yellow solid; [α]_D_^20^ -24 (c 0.1 g/100 mL, CH_3_OH). ^1^H-NMR (500 MHz, CD_3_OD) *δ* 7.26 (2H, m, H-9′), 7.25 (2H, m, H-8′, H-9′), 7.18 (1H, m, H-10′), 6.72 (1H, dt, *J* = 15.1, 7.0, H-3), 5.88 (1H, d, *J* = 15.1, H-2), 5.41 (2H, m, H-8, H-9), 4.71 (1H, ddd, *J* = 8.3, 4.3, 2.9, H-4′), 4.40 (1H, ddd, *J* = 9.3, 7.3, 2.8, H-5′), 4.32 (1H, dd, *J* = 7.1, 8.2, H-2′), 2.99 (1H, dd, *J* = 14.0, 6.1, H-6′), 2.85 (1H, dd, *J* = 14.0, 9.3, H-6′), 2.41 (1H, ddd, *J* = 13.7, 8.6, 4.3, H-3′), 2.17 (3H, m, H-4, H-3′), 1.98 (2H, m, H-7), 1.63 (3H, d, *J* = 4.1, H-10), 1.43 (2H, m, H-5), 1.35 (2H, m, H-6). ^13^C-NMR (600 MHz, CD_3_OD) *δ* 178.4 (C-1′), 169.0 (C-1), 146.8 (C-3), 139.0 (C-7′), 132.3 (C-8), 130.2 (C-8′), 129.5 (C-9′), 127.6 (C-10′), 126.0 (C-9), 124.0 (C-2), 80.7 (C-4′), 68.0 (C-2′), 54.7 (C-5′), 38.7 (C-6′), 34.6 (C-3′), 33.3 (C-7), 32.9 (C-4), 30.1 (C-6), 28.8 (C-5), 18.1 (C-10). ESI-HRMS: 372.2182 ([M + H]^+^, C_22_H_30_NO_4_^+^; calc. 372.2169, err. 3.49 ppm), 394.1998 ([M + Na]^+^, C_22_H_29_NO_4_Na^+^; calc. 394.1989, err. 2.28 ppm), 743.4272 ([2 M + H]^+^, C_44_H_59_N_2_O_8_^+^; calc. 743.4266, err. 0.81 ppm).

Colletamide C **(9)**, (*S*)-2-[(2*E*,8*E*)-deca-2,8-dienamido]-3-(2-hydroxyphenyl)propionic acid: yellow solid; [α]_D_^20^ -8 (c 0.1 g/100 mL, CH_3_OH). ^1^H-NMR (500 MHz, CD_3_OD) *δ* 7.08 (1H, dd, *J* = 7.5, 1.2, H-9′), 7.03 (1H, td, *J* = 7.6, 1.2, H-7′), 6.75 (1H, d, *J* = 7.6, H-6′), 6.74 (1H, t, *J* = 7.5, H-8′), 6.69 (1H, dt, *J* = 15.2, 7.0, H-3), 5.89 (1H, d, *J* = 15.3, H-2), 5.41 (2H, m, H-8, H-9), 4.67 (1H, dd, *J* = 8.7, 5.2, H-2′), 3.21 (1H, dd, *J* = 13.9, 5.0, H-3′), 2.98 (1H, dd, *J* = 13.9, 8.9, H-3′), 2.17 (2H, ddd, *J* = 1.2, 7.3, 14.0, H-4), 1.99 (2H, m, H-7), 1.63 (3H, d, *J* = 3.7, H-10), 1.44 (2H, m, H-5), 1.37 (2H, m, H-6). ^13^C-NMR (600 MHz, CD_3_OD) *δ* 176.9 (C-1′), 168.4 (C-1), 156.7 (C-5′), 145.7 (C-3), 132.3 (C-8), 132.2 (C-9′), 128.9 (C-7′), 125.9 (C-9), 125.4 (C-4′), 124.7 (C-2), 120.5 (C-8′), 116.2 (C-6′), 55.6 (C-2′), 33.6 (C-3′), 33.4 (C-7), 32.9 (C-4), 30.2 (C-6), 28.9 (C-5), 18.1 (C-10). ESI-HRMS: 332.1859 ([M + H]^+^, C_19_H_26_NO_4_^+^; calc. 332.1856, err. 0.90 ppm), 395.1948 ([M + ACN + Na]^+^, C_21_H_28_N_2_O_4_Na^+^; calc. 395.1941, err. 1.77 ppm), 663.3648 ([2 M + H]^+^, C_38_H_51_N_2_O_8_^+^; calc. 663.3640, err. 1.21 ppm).

Colletamide D **(10)**, (3*S*,4*R*)-4-[(2*E*,8*E*)-deca-2,8-dienamido)-3-hydroxy-5-phenylpentanoic acid: yellow solid; [α]_D_^20^ -5 (c 0.1 g/100 mL, CH_3_OH). ^1^H-NMR (500 MHz, CD_3_OD) *δ* 7.25 (2H, m, H-7′, H-11′), 7.23 (2H, m, H-8′, H-10′), 7.14 (1H, m, H-9′), 6.68 (1H, dt, *J* = 15.3, 7.0, H-3), 5.94 (1H, d, *J* = 15.4, H-2), 5.42 (2H, m, H-8, H-9), 4.14 (1H, td, *J* = 7.2, 2.0, H-4′), 4.02 (1H, td, *J* = 6.0, 1.8, H-3′), 2.97 (1H, dd, *J* = 6.4, 13.4, H-5′), 2.81 (1H, dd, *J* = 8.4, 13.4, H-5′), 2.30 (2H, m, H-2′), 2.16 (2H, m, H-4), 1.99 (2H, m, H-7), 1.63 (3H, d, *J* = 4.0, H-10), 1.44 (2H, m, H-5), 1.35 (2H, m, H-6). ^13^C-NMR (600 MHz, CD_3_OD) *δ* 179.7 (C-1′), 168.6 (C-1), 145.7 (C-3), 140.2 (C-6′), 132.2 (C-8), 130.3 (C-7′), 129.3 (C-8′), 127.2 (C-9′), 126.0 (C-9), 124.7 (C-2), 70.1 (C-3′), 56.3 (C-4′), 41.6 (C-2′), 38.3 (C-5′), 33.4 (C-7), 32.9 (C-4), 30.2 (C-6), 28.9 (C-5), 18.1 (C-10). ESI-HRMS: 360.2170 ([M + H]^+^, C_21_H_30_NO_4_^+^; calc. 360.2169, err. 0.28 ppm), 382.1995 ([M + Na]^+^, C_21_H_29_NO_4_Na^+^; calc. 382.1989, err. 1.57 ppm), 719.4293 ([2M + H]^+^, C_42_H_59_N_2_O_8_^+^; calc. 719.4266, err. 3.75 ppm).

### X-ray analysis

X-ray diffraction data for cyclo-(Phe-Leu-Leu-Leu-Ile) was collected by using a VENTURE PHOTON100 CMOS Bruker diffractometer with Micro-focus IuS source Cu Kα radiation. Crystal was mounted on a CryoLoop (Hampton Research) with Paratone-N (Hampton Research) as cryoprotectant and then flashfrozen in a nitrogen-gas stream at 100 K^64^. The temperature of the crystal was maintained at the selected value by means of an N-Helix cooling device to within an accuracy of ± 1 K. The data were corrected for Lorentz polarization, and absorption effects. The structures were solved by direct methods using SHELXS-97^65^ and refined against *F*^2^ by full-matrix least-squares techniques using SHELXL-2018^66^ with anisotropic displacement parameters for all non-hydrogen atoms. Hydrogen atoms were introduced into the calculations as a riding model with isotropic thermal parameters. All calculations were performed by using the Crystal Structure crystallographic software package WINGX^67^.

The crystal data collection and refinement parameters are given in Table S7.

CCDC 1,944,171 contains the supplementary crystallographic data for this paper. These data can be obtained free of charge from the Cambridge Crystallographic Data Centre via https://www.ccdc.cam.ac.uk/Community/Requestastructure.

### Computational details

All calculations have been performed using Gaussian 16W. Prior to DFT calculations, a conformational analysis has been performed using the GMMX plugin. Each conformer has been optimized using DFT at the B3LYP/6-31g(d) level. Frequency calculation have been performed at the same level of theory. Rotational strengths have been calculated using the B3LYP/6-311+g(d,p) for 20 excited states. ECD spectra were plotted using the Gaussview 6. GIAO NMR properties were predicted using the MPW1PW91/6-311+g(2d,p). Theoretical and experimental NMR chemical shift were compared using common metrics such as linear correlation (R^2^), mean average error (MAE) and DP4 probability [ \* MERGEFORMAT 33].

### Cytotoxicity evaluation

For each strain, a crude extract was tested to determine its cytotoxicity at 10 µg/ml using normal human lung fibroblast cells MRC-5 (ATCC CCL-171). The assay was conducted according to the procedure described by Tempête et al*.*^50^. Docetaxel (Sigma-Aldrich, France) was used as positive controls. Docetaxel show 0.0005% mortality at 1 μg/mL.

## Supplementary information

Supplementary Information.
