# Supplementary material for: Identification and dereplication of endophytic Colletotrichum strains by MALDI TOF mass spectrometry and molecular networking
Source: Sci Rep. 2020 Nov 13;10:19788. doi: 10.1038/s41598-020-74852-w (PMC7666161; doi:10.1038/s41598-020-74852-w)
Supplement: Supplementary file 1 — Supplementary Information. [file 41598_2020_74852_MOESM1_ESM.docx]

**Supplementary data**

**IDENTIFICATION AND DEREPLICATION OF ENDOPHYTIC *COLLETOTRICHUM* STRAINS BY MALDI TOF MASS SPECTROMETRY AND MOLECULAR NETWORKING**

*Morgane Barthélemy,^1^ Vincent Guérineau^1^, Grégory Genta-Jouve^2,3^, Mélanie Roy^4^, Jérôme Chave^4^, Régis Guillot^5^, Léonie Pellissier^6^, Jean-Luc Wolfender^6^, Didier Stien^7^, Véronique Eparvier^*,1^, David Touboul^*,1^*

* VE and DT are co-corresponding authors. *Correspondence to : [david.touboul@cnrs.fr](mailto:david.touboul@cnrs.fr) / [veronique.eparvier@cnrs.fr](mailto:veronique.eparvier@cnrs.fr)

*1* Université Paris-Saclay, CNRS, Institut de Chimie des Substances Naturelles, UPR 2301, 91198, Gif-sur-Yvette, France.

*2 UMR 8038 CiTCoM, Faculté de Pharmacie de Paris, Université Paris Descartes, Avenue de l’observatoire, 75006 Paris, France*

*3 Molecules of Communication and Adaptation of Microorganisms (UMR 7245), National Museum of Natural History, CNRS, Paris, France*

*4 Laboratoire Evolution et Diversité Biologique, UPS- CNRS - IRD - UMR 5174, Université Paul Sabatier (Toulouse 3), 118 route de Narbonne 31062, Toulouse, France.*

*5 ICMMO, Université Paris-Saclay – UMR CNRS 8182, rue du doyen Georges Poitou, 91405 Orsay, France*

*6* *School of Pharmaceutical Sciences, Institute of Pharmaceutical Sciences of Western Switzerland, University of Geneva, Rue Michel Servet 1, CH-1211, Geneva, Switzerland.*

*7 Sorbonne Université, CNRS, Laboratoire de Biodiversité et Biotechnologies Microbiennes, Observatoire Océanologique, Banyuls/Mer*

Table S1: Identification of the 42 *Colletotrichum* strains and cytotoxic activity of their EtOAc extract

| **ID Codes** | **Closest species in NCBI (accession number)** | **Query cover (%)** | **Identity (%)** | **NCBI accession number** | **Cytotoxicity on MRC-5** |
| --- | --- | --- | --- | --- | --- |
| BSNB-0529 | *Colletotrichum gloeosporioides* (KR995714) | 100 | 99 | MK300813 | 105 ± 4 |
| BSNB-0530 |  |  |  | MK300820 | 107 ± 10 |
| BSNB-0536 |  |  |  | MK300818 | 9 ± 1 |
| BSNB-0537 |  |  |  | MK300815 | 106 ± 9 |
| BSNB-0540 |  |  |  | MK300823 | 79 ± 5 |
| BSNB-0549 |  |  |  | MK300811 | 105 ± 11 |
| BSNB-0580 |  |  |  | MK300812 | 3 ± 1 |
| BSNB-0590 |  |  |  | MK300816 | 12 ± 1 |
| BSNB-0622 |  |  |  | MK300822 | 68 ± 2 |
| BSNB-0646 |  |  |  | MK300814 | 102 ± 7 |
| BSNB-0649 |  |  |  | MK300817 | 91 ± 4 |
| BSNB-0653 |  |  |  | MK300821 | 107 ± 10 |
| BSNB-0655 |  |  |  | MK300810 | 98 ± 1 |
| BSNB-0696 |  |  |  | MK300819 | 86 ± 1 |
| BSNB-0290 | *Colletotrichum gloeosporioides* (MH865232) | 100 | 100 | MK300790 | 110 ± 4 |
| BSNB-0538 |  |  |  | MK300793 | 90 ± 2 |
| BSNB-0628 |  |  |  | MK300792 | 81 ± 8 |
| BSNB-0637 |  |  |  | MK300797 | 12 ± 1 |
| BSNB-0652 |  |  |  | MK300791 | 31 ± 1 |
| BSNB-0662 |  |  |  | MK300787 | 95 ± 2 |
| BSNB-0688 |  |  |  | MK300795 | 98 ± 4 |
| BSNB-0694 |  |  |  | MK300794 | 74 ± 4 |
| BSNB-0699 |  |  |  | MK300788 | 102 ± 3 |
| BSNB-0703 |  |  |  | MK300786 | 75 ± 1 |
| BSNB-1019 |  |  |  | MK300789 | 91 ± 1 |
| BSNB-1021 |  |  |  | MK300796 | 99 ± 2 |
| BSNB-0551 | *Colletotrichum gloeosporioides* (MH864569) | 100 | 99 | MK300784 | 30 ± 3 |
| BSNB-0559 |  |  |  | MK300782 | 11 ± 2 |
| BSNB-0574 |  |  |  | MK300785 | 110 ± 3 |
| BSNB-0625 |  |  |  | MK300783 | 100 ± 1 |
| BSNB-0641 | *Colletotrichum gloeosporioides* (KT004429) | 100 | 99 | MK300805 | 9 ± 2 |
| BSNB-0650 |  |  |  | MK300808 | 7 ± 3 |
| BSNB-0654 |  |  |  | MK300807 | 107 ± 10 |
| BSNB-0701 |  |  |  | MK300806 | 73 ± 2 |
| BSNB-0623 | *Colletotrichum* sp. strain LGMF1580 (MG976389) | 100 | 99 | MK300800 | 97 ± 18 |
| BSNB-0627 |  |  |  | MK300801 | 13 ± 2 |
| BSNB-0682 |  |  |  | MK300802 | 73 ± 2 |
| BSNB-0645 | *Colletotrichum gloeosporioides* (MH866036) | 100 | 99 | MK300799 | 81 ± 6 |
| BSNB-0695 |  |  |  | MK300798 | 91 ± 1 |
| BSNB-0557 | *Colletotrichum vietnamense* (MH863700) | 99 | 100 | MK300804 | 116 ± 3 |
| BSNB-0583 | *Colletotrichum* sp. strain AHGB10 (MH267867) | 99 | 99 | MK300803 | 32 ± 1 |
| BSNB-0615 | *Colletotrichum* sp. strain AHGB15 (MH267881) | 99 | 99 | MK300809 | 23 ± 2 |

**Figure S1:** a) Comparison of the eighteen different protein fingerprints from the 42 Colletotrichum strains isolated from Astrocaryum sciophilum palms. Origin 6.0 (<https://www.originlab.com/>). b) Phylogenetic tree derived from a neighbour-joining analysis of an alignment of ITS sequences (1000 bootstrap replicates). *Monilochaetes infuscans* (JQ005780) is used as the output group. Closest *Colletotrichum* sequences as determined by BLAST and included in the tree. ^a^ MRC-5 cell viability of the EtOAc extract of each strain at 10 µg/ml (% cell viability compared to negative control group, positive control: Docetaxel). No correlation was finally enlightened.

a)

b)


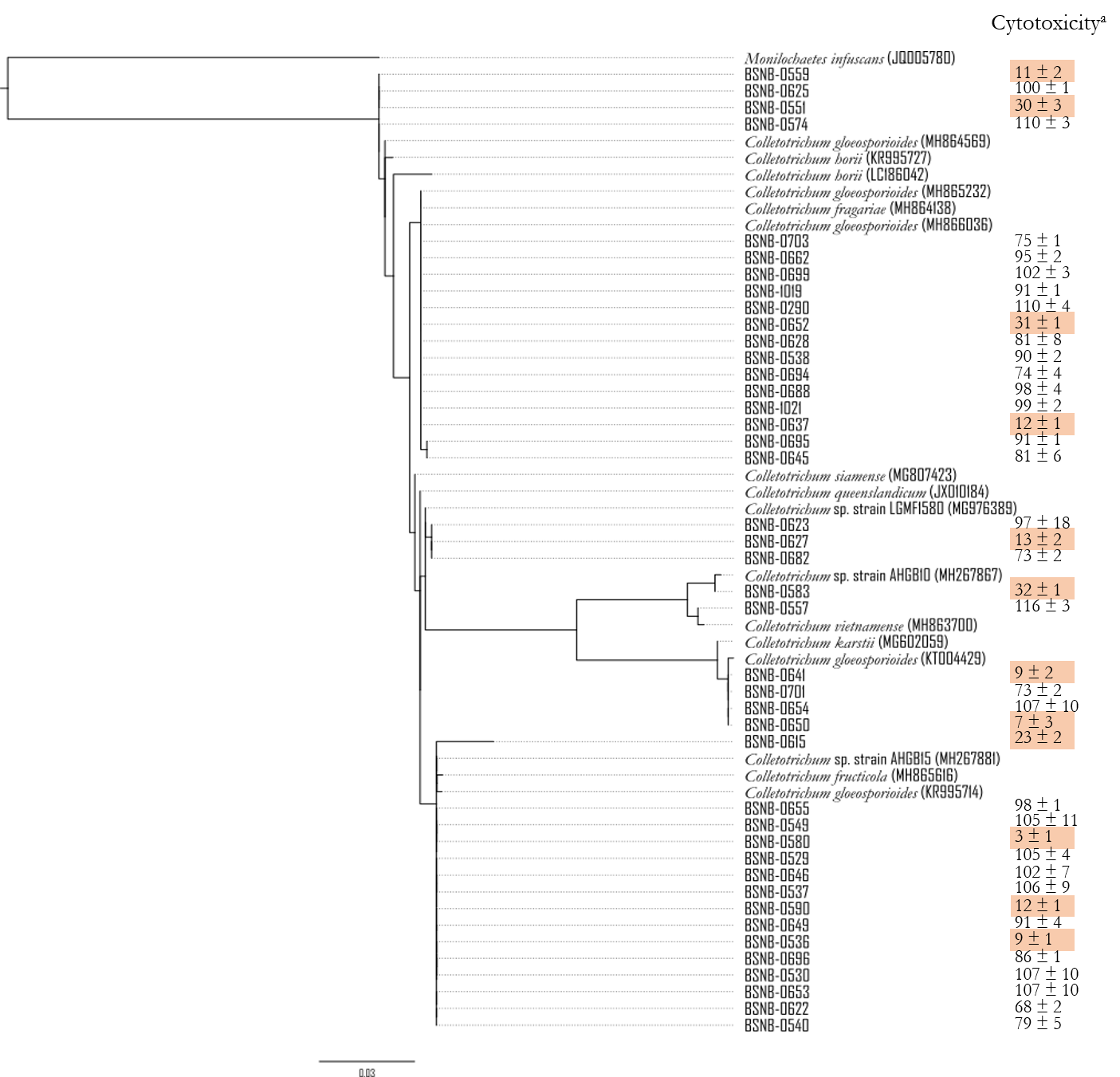


**Figure S2:** Phylogenetic tree derived from a neighbour-joining analysis of an alignment of ITS sequences of the 42 *Colleotrichum* strains (1000 bootstrap replicates). *Monilochaetes infuscans* (JQ005780) is used as the output group. Closest Colletotrichum sequences were determined by BLAST and included in the tree

**
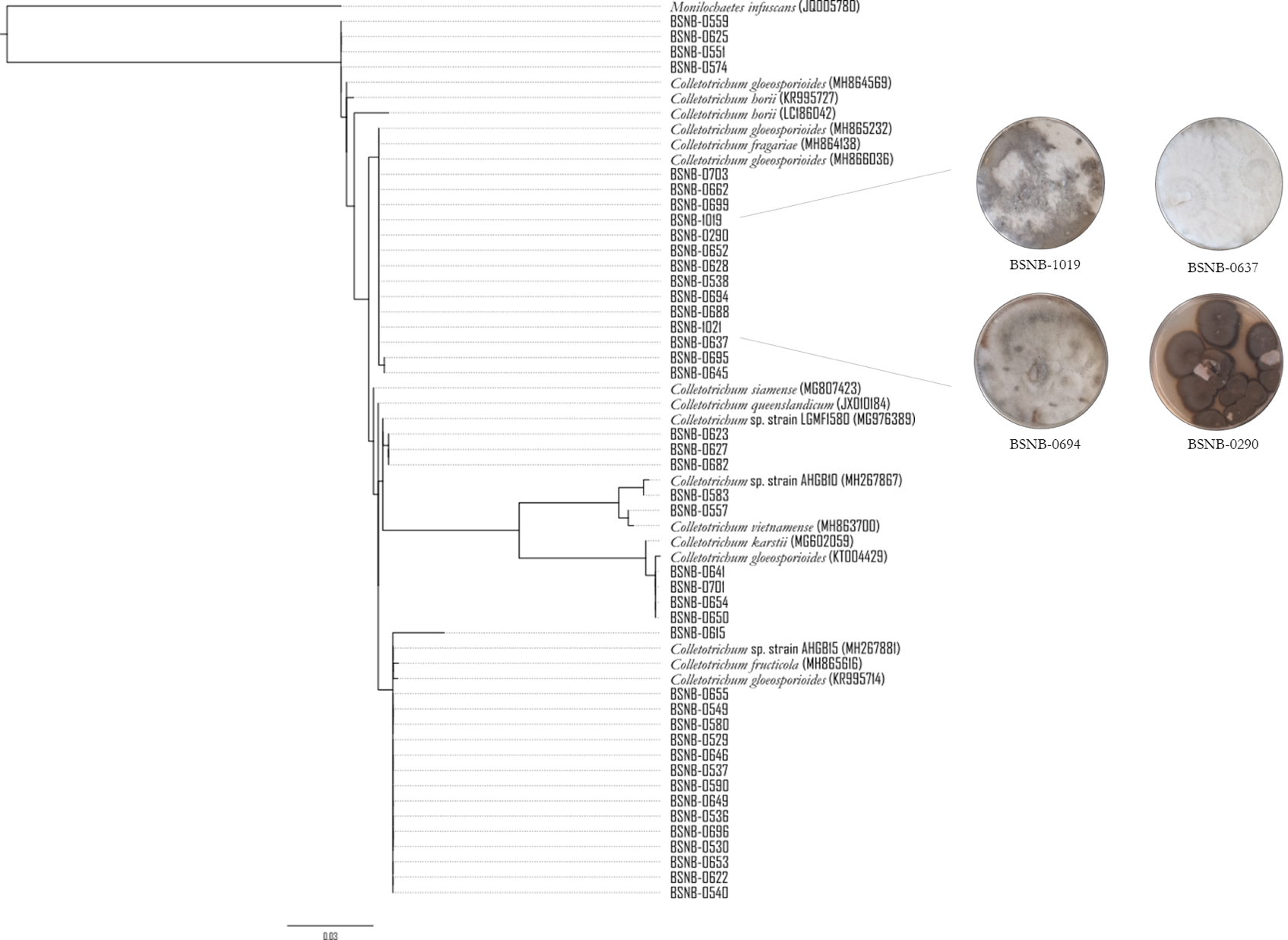
**

**Figure S3:** Proteins fingerprint of strains BSNB-0529, BSNB-0537, BSNB-0540, BSNB-0574, BSNB-0623, BSNB-0645, BSNB-0649, BSNB-0653, BSNB-0655, BSNB-0662 and BSNB-0696


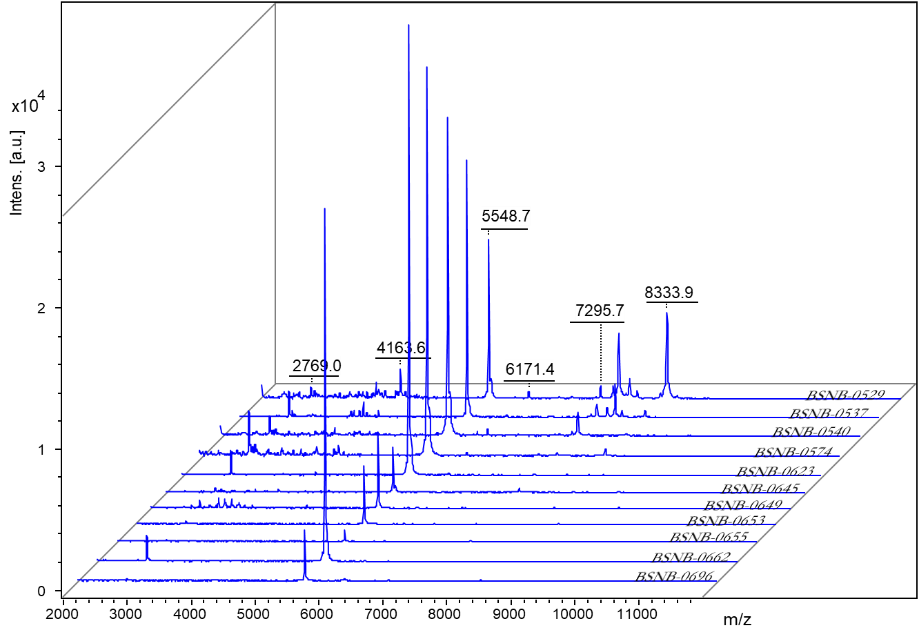


**Figure S4:** Proteins fingerprint of strains BSNB-0530

**
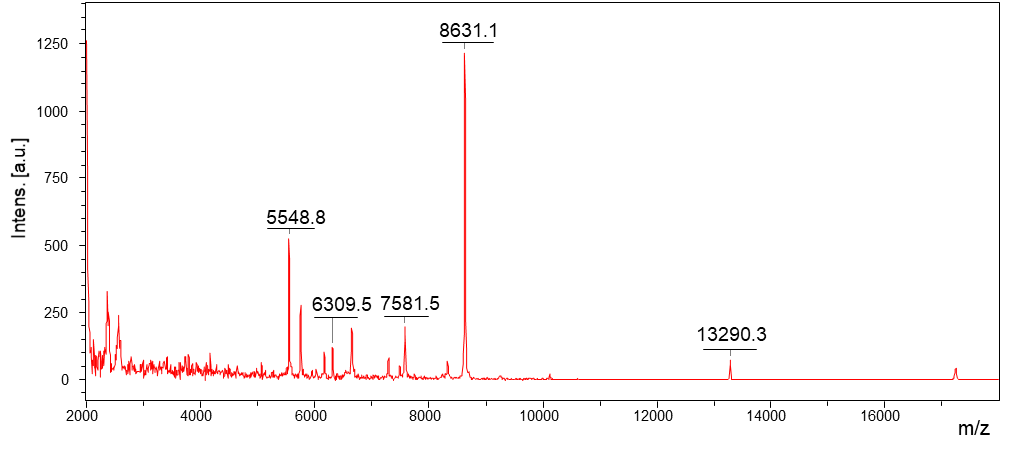
**

**Figure S5:** Proteins fingerprint of strains BSNB-0536, BSNB-0551, BSNB-0559, BSNB-0637 and BSNB-0641

**
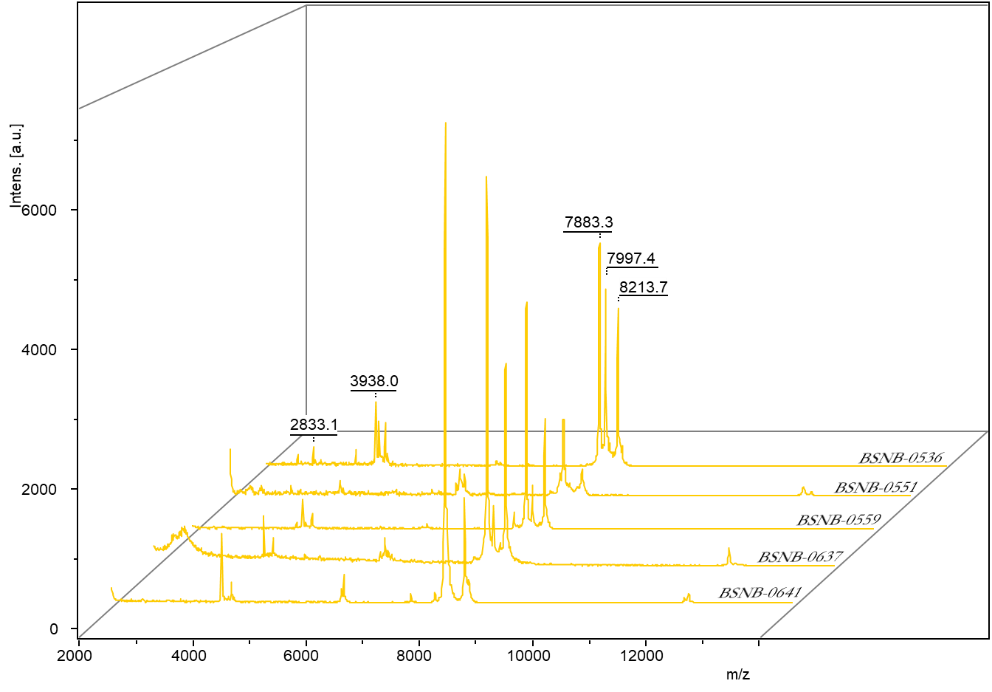
**

**Figure S6:** Proteins fingerprint of strains BSNB-0590 and BSNB-0650


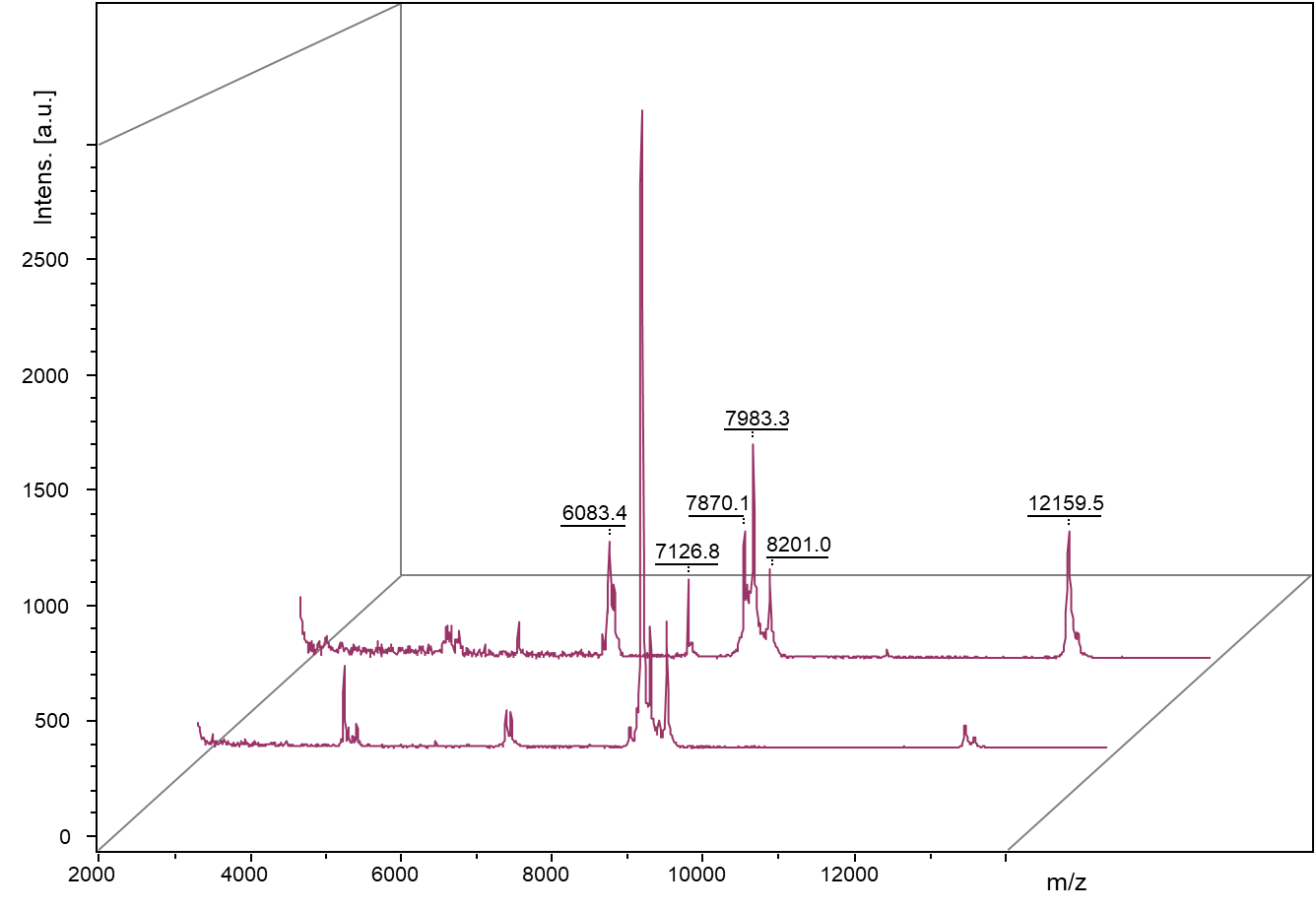


**Figure S7:** Proteins fingerprint of strains BSNB-0615 and BSNB-0652

**
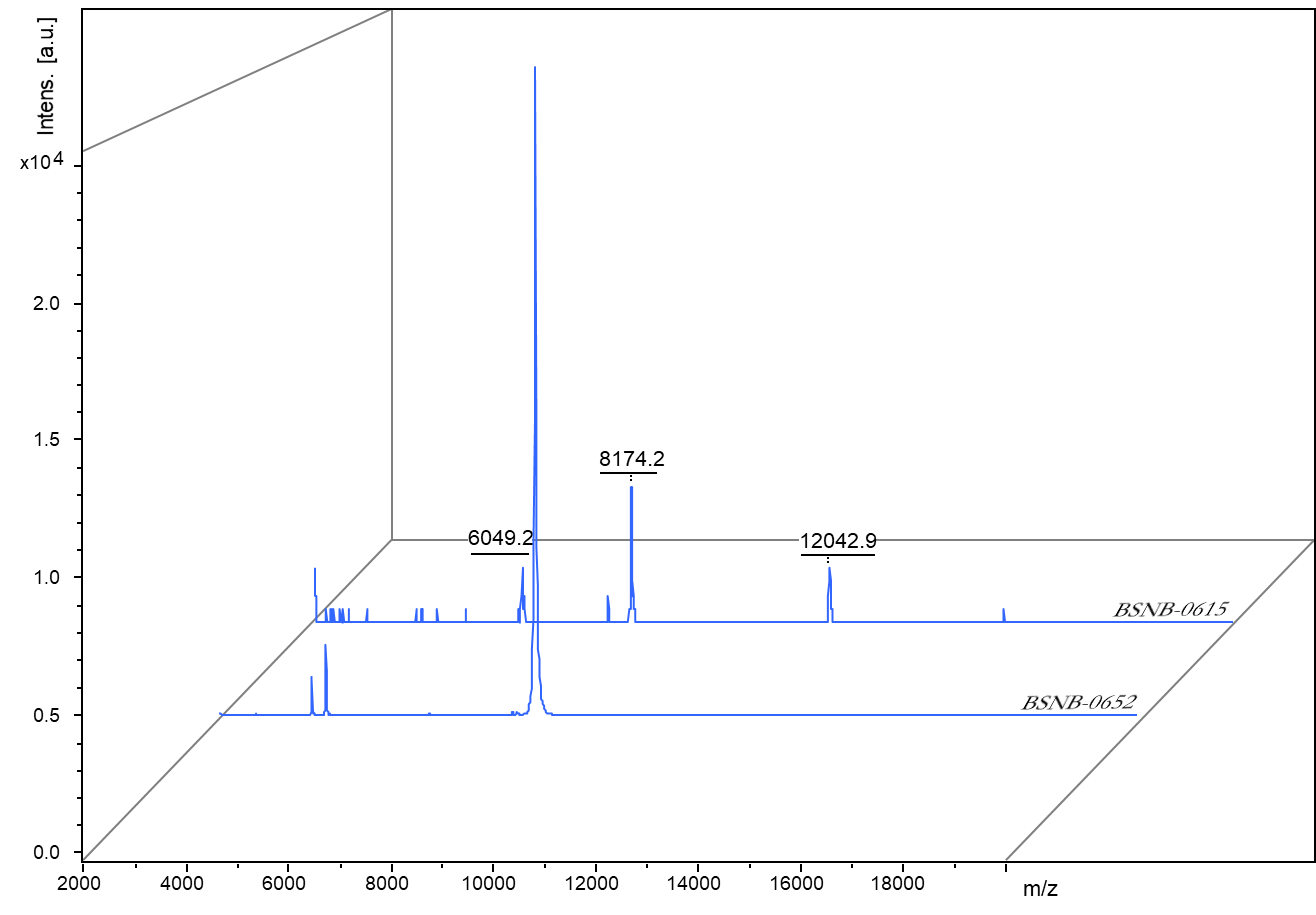
**

**Figure S8:** Proteins fingerprint of strains BSNB-0625, BSNB-0627, BSNB-0628, BSNB-0695, BSNB-0699, BSNB-0703 and BSNB-1021


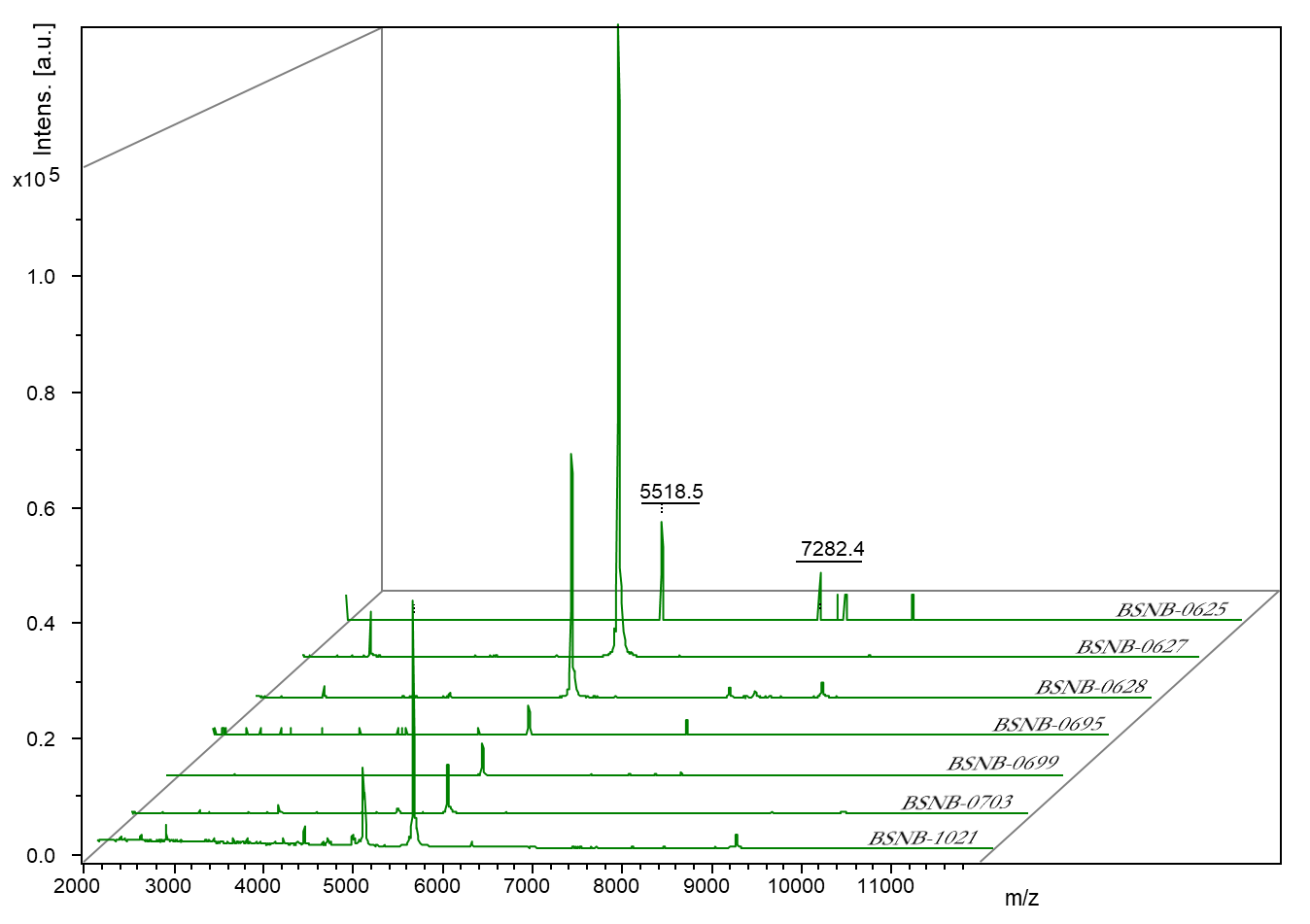


**Figure S9:** Proteins fingerprint of strains BSNB-0538


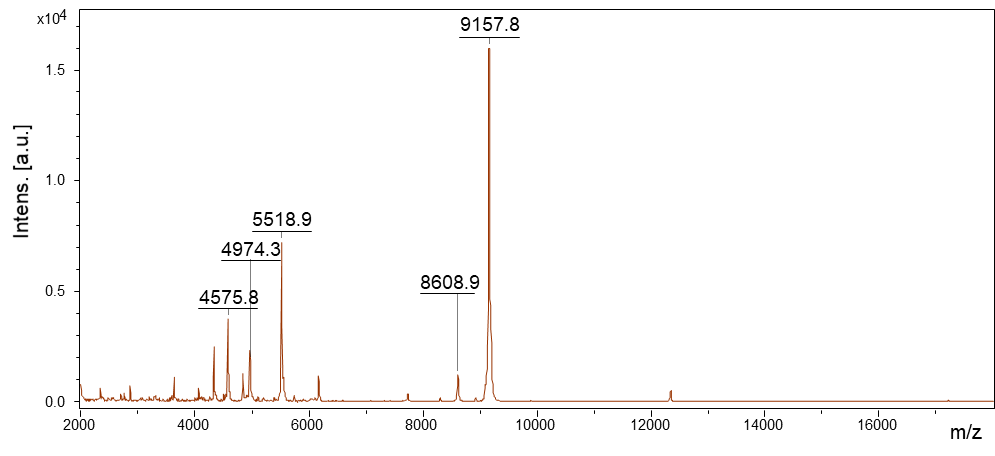


**Figure S9:** Proteins fingerprint of strains BSNB-0290

**
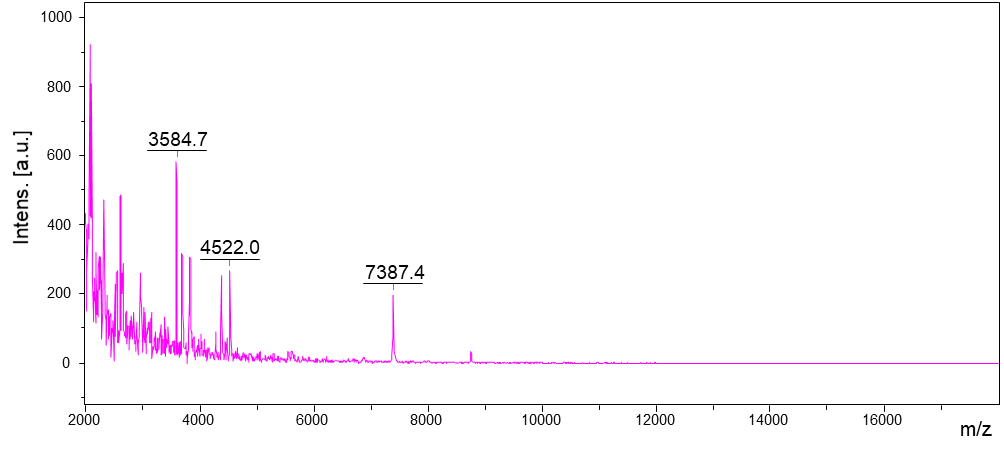
**

**Figure S11:** Proteins fingerprint of strains BSNB-0549

**
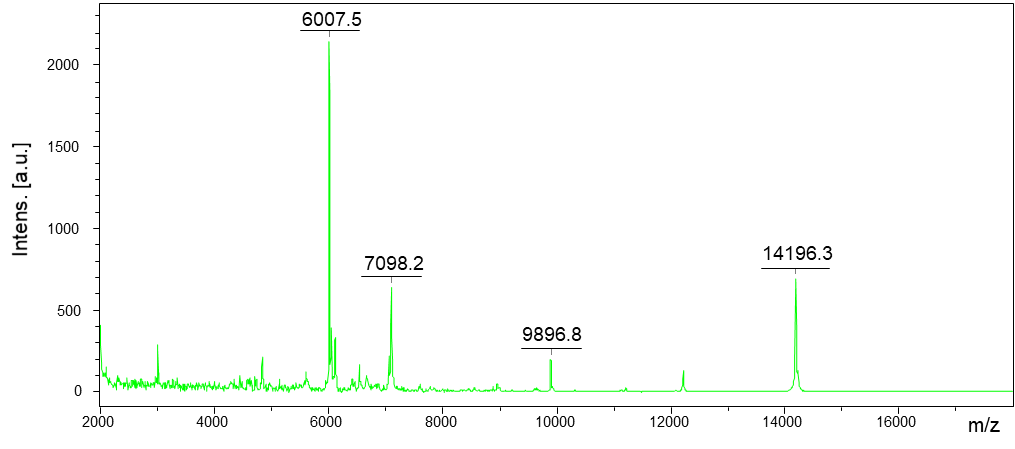
**

**Figure S12:** Proteins fingerprint of strains BSNB-0557

**
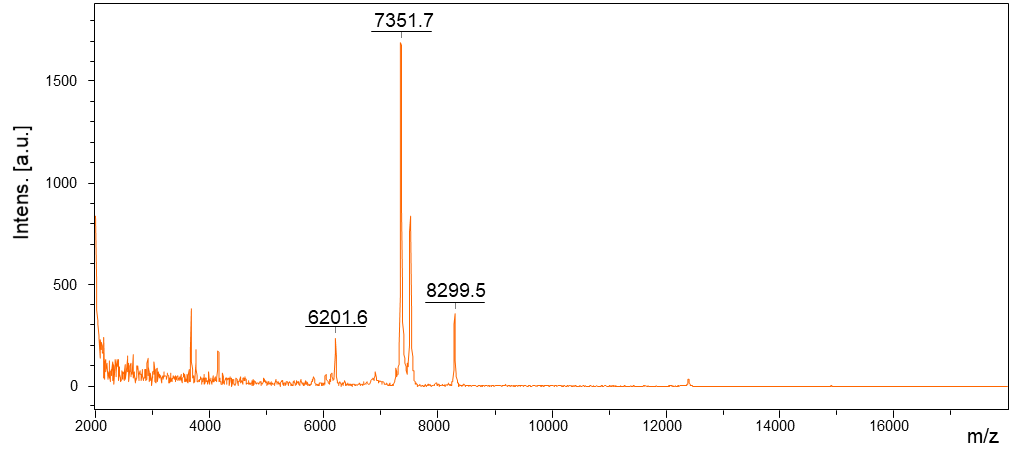
**

**Figure S13:** Proteins fingerprint of strains BSNB-0580

**
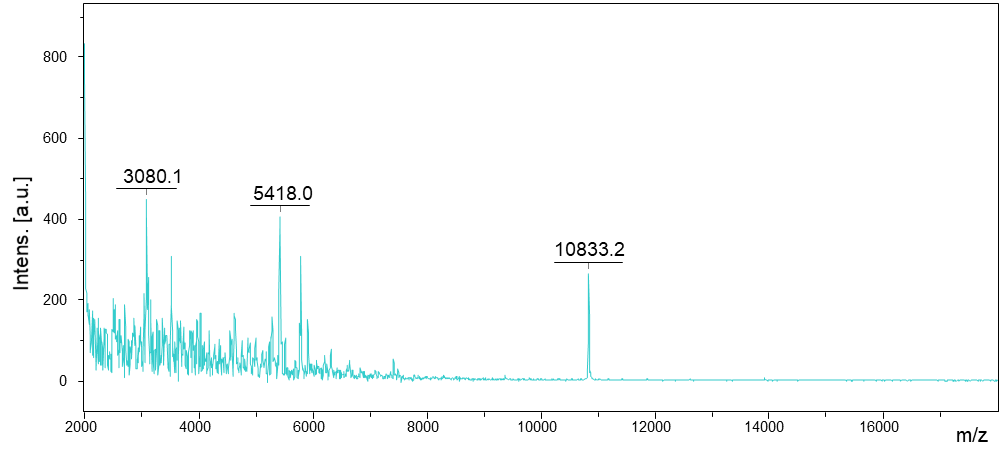
**

**Figure S14:** Proteins fingerprint of strains BSNB-0583

**
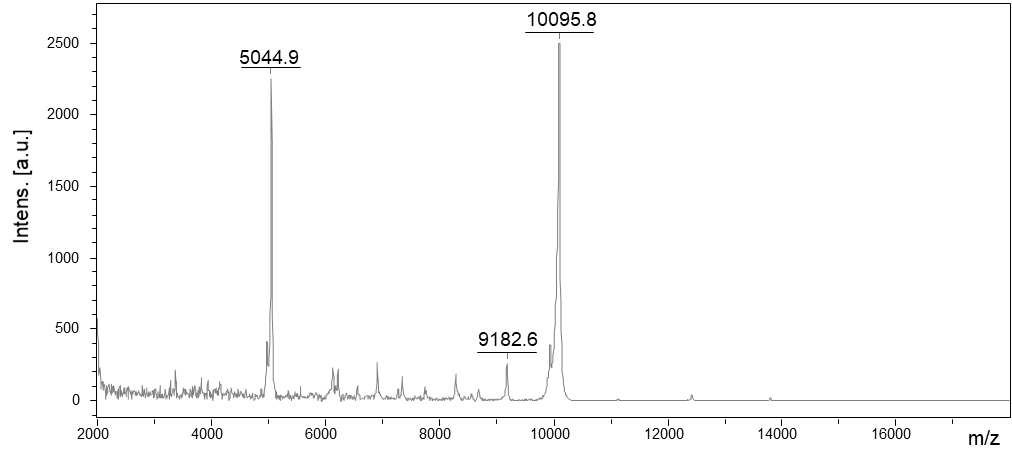
**

**Figure S15:** Proteins fingerprint of strains BSNB-0622

**
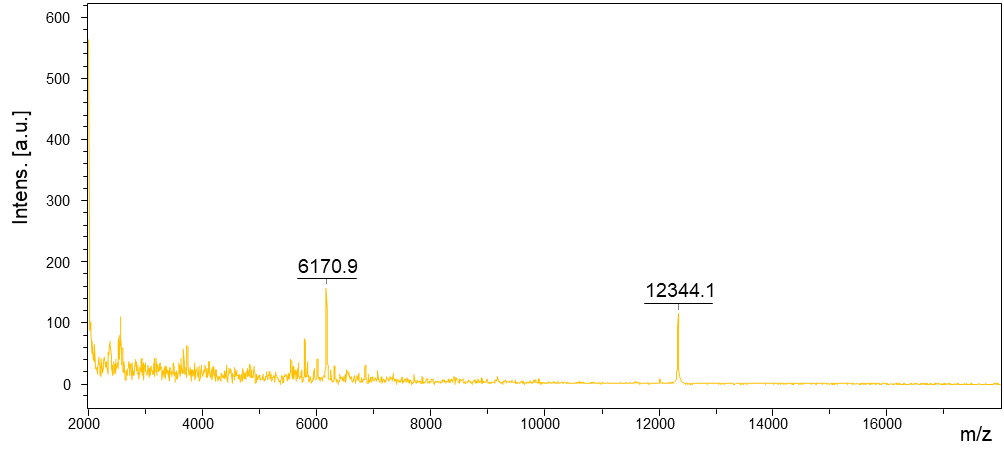
**

**Figure S16:** Proteins fingerprint of strains BSNB-0646

**
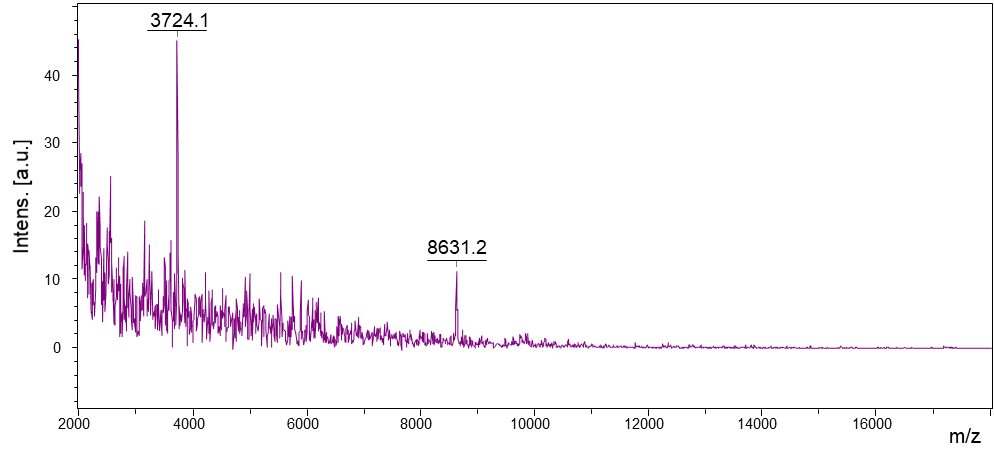
**

**Figure S17:** Proteins fingerprint of strains BSNB-0654

**
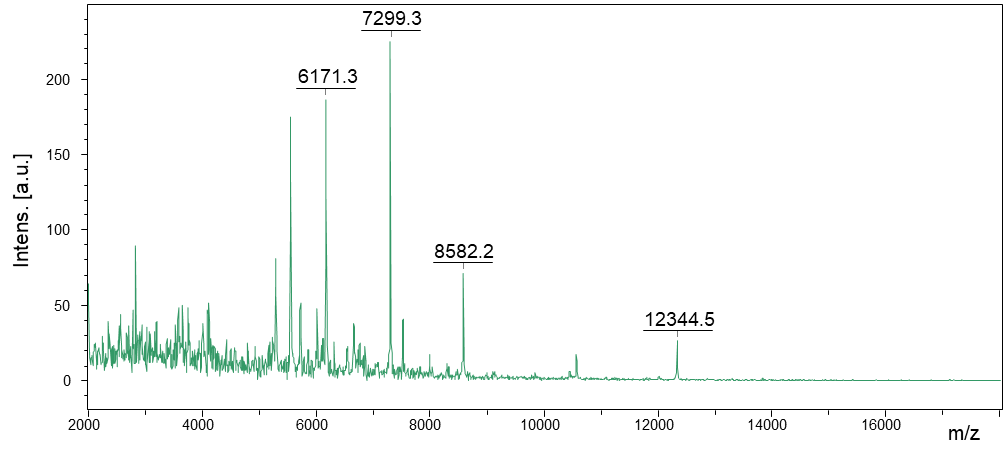
**

**Figure S18:** Proteins fingerprint of strains BSNB-0682

**
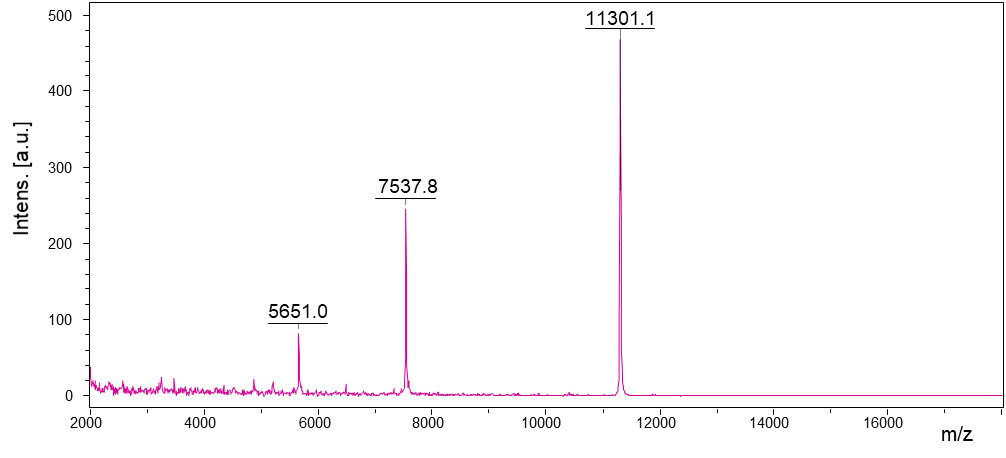
**

**Figure S19:** Proteins fingerprint of strains BSNB-0688

**
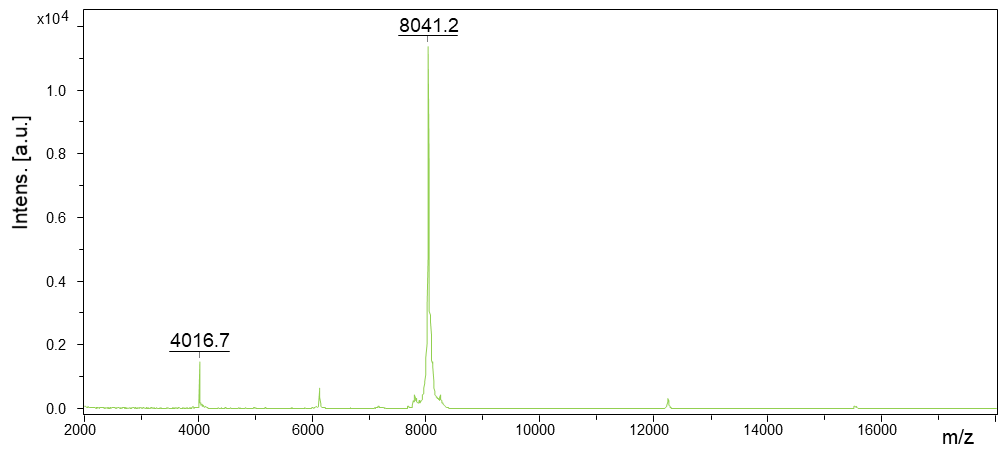
**

**Figure S20:** Proteins fingerprint of strains BSNB-0694

**
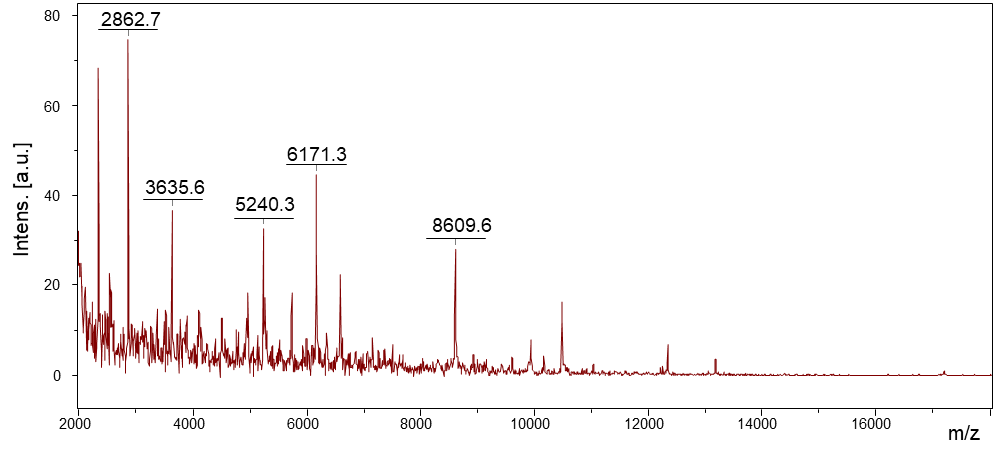
**

**Figure S21:** Proteins fingerprint of strains BSNB-1019

**
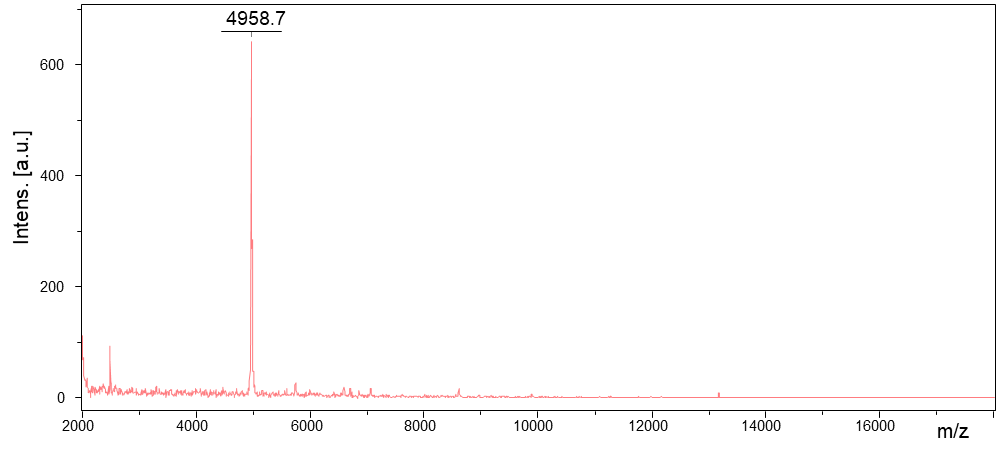
**

**Figure S22:** Proteins fingerprint of strains BSNB-0701


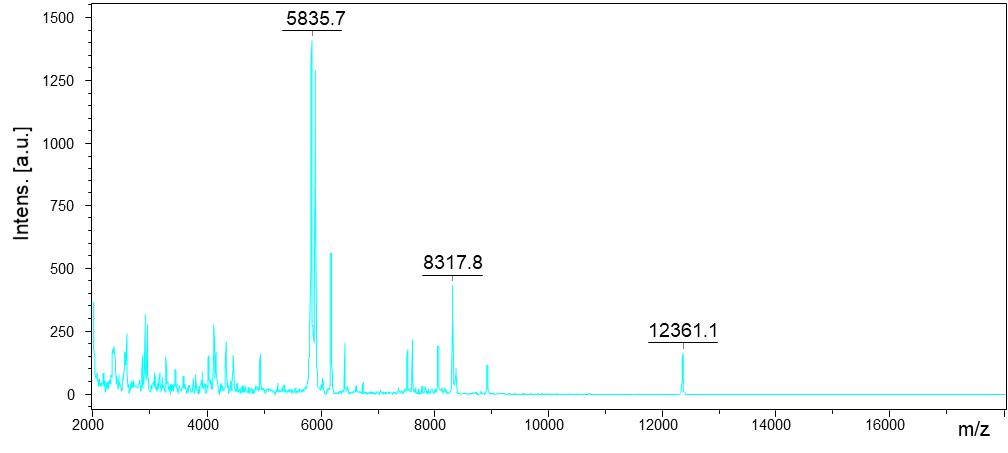


**Figure S23: (A)** Hierarchical clustering of 40 *Colletotrichum* strains, depending on the major protein extracted from the proteins fingerprint. In green the ions present in the protein extract, in red ions not detected. **(B)** Phylogenetic tree derived from a neighbour-joining analysis of an alignment of proteins fingerprints

**A**
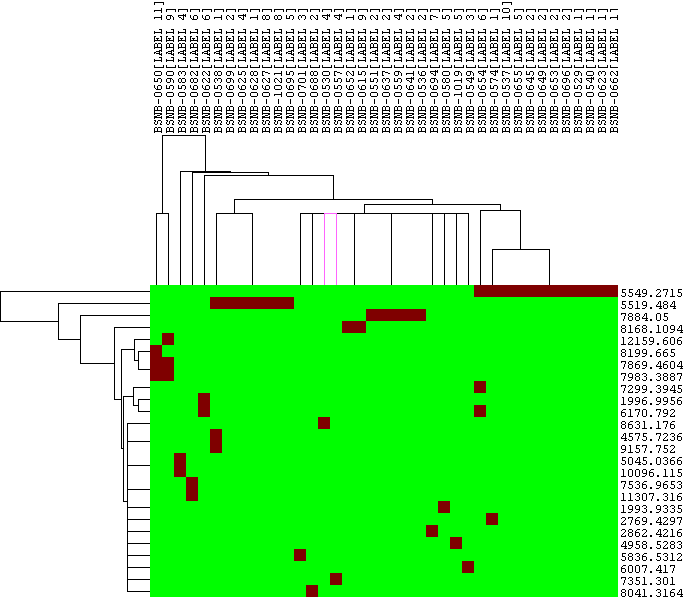


**B**


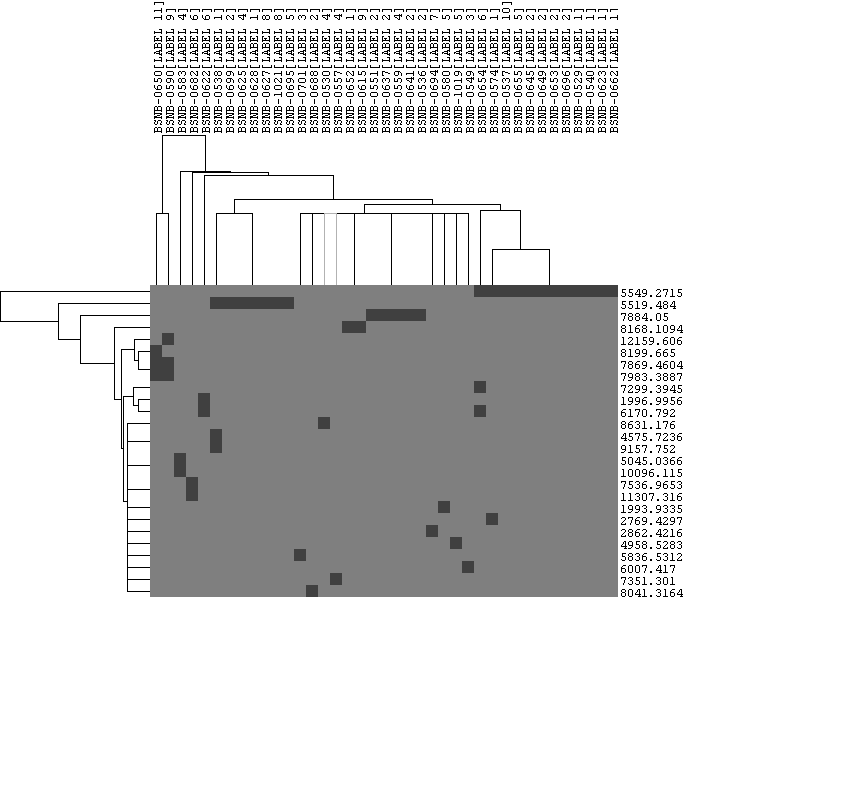

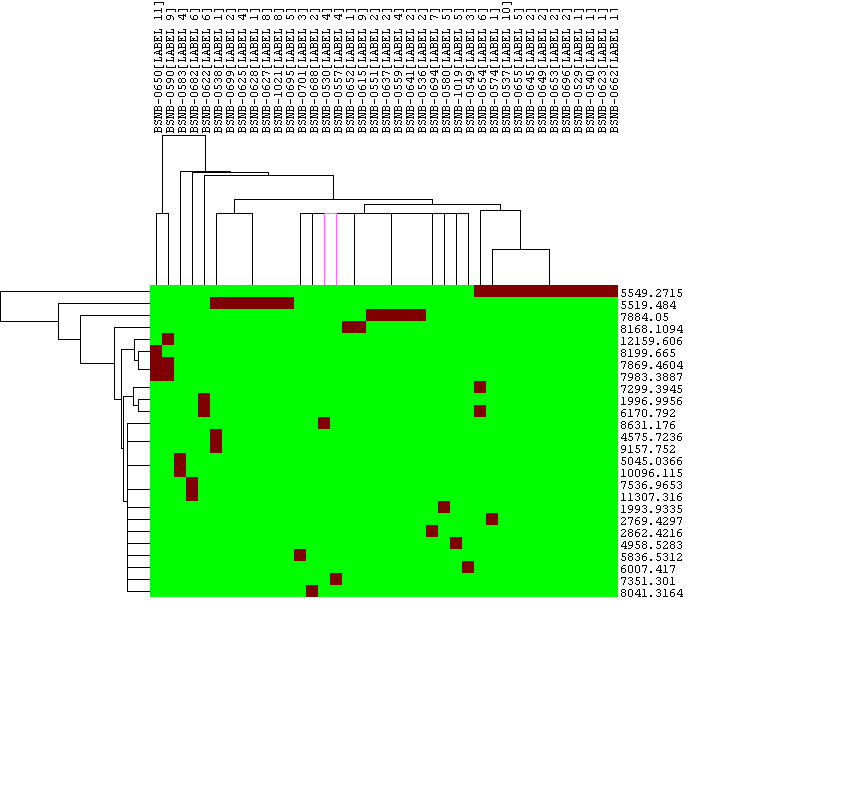


**Figure S24:** Molecular network of the 42 EtOAc extracts from *Colletotrichum* strains. Relative quantification of each ion within the extracts are represented as a XIC area-dependent pie-chart drawing. Mapping of the nodes are done depending the clusterisation in species via the MALDI-TOF MS fingerprinting


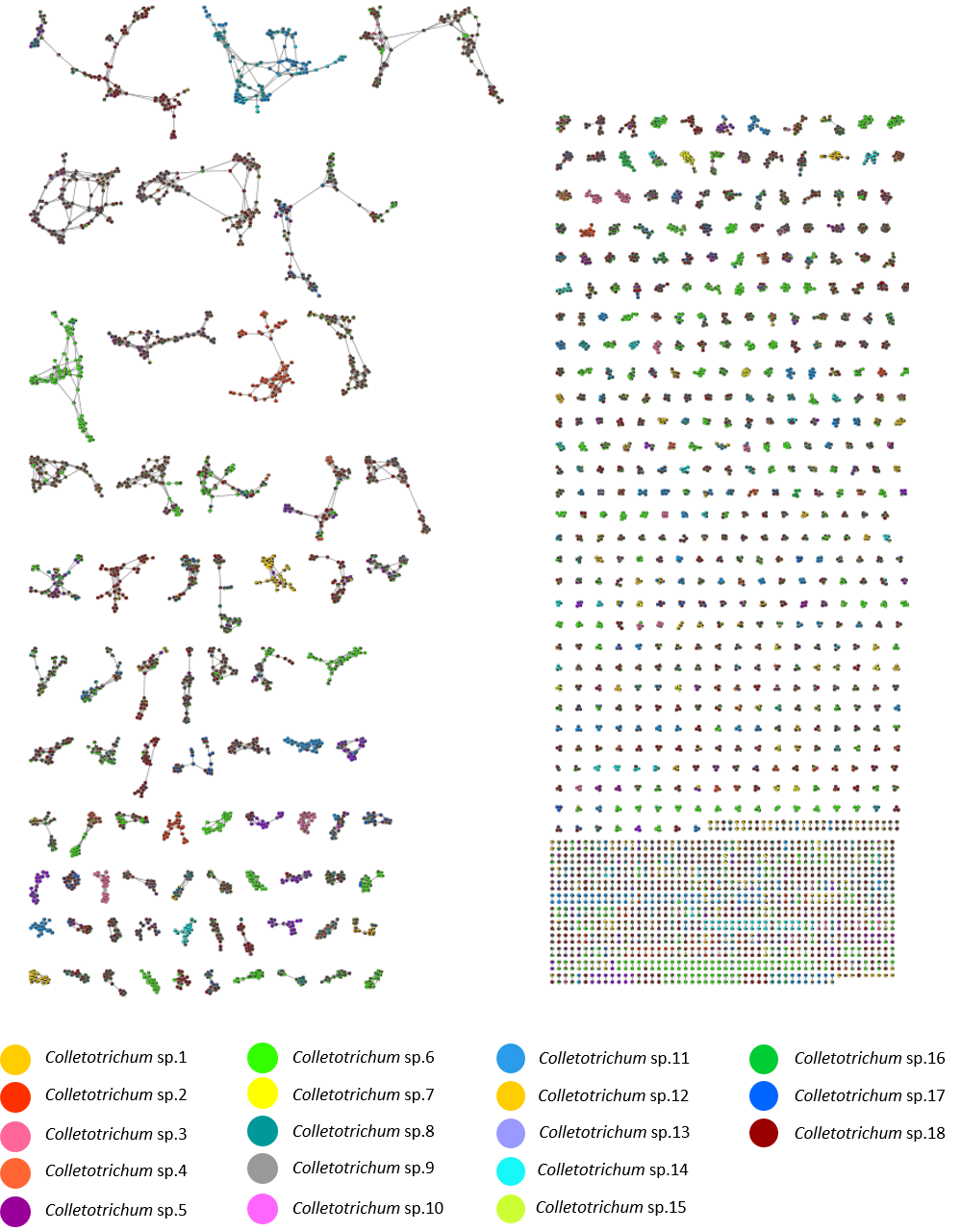


**Figure S25:** Molecular network of the 42 EtOAc extracts from *Colletotrichum* strains. Relative quantification of each ion within the extracts are represented as a XIC area-dependent pie-chart drawing. Mapping of the nodes are done depending the cytotoxicity of the species. Nodes in grey are from extracts with a viability on MRC-5 cells > 30%.


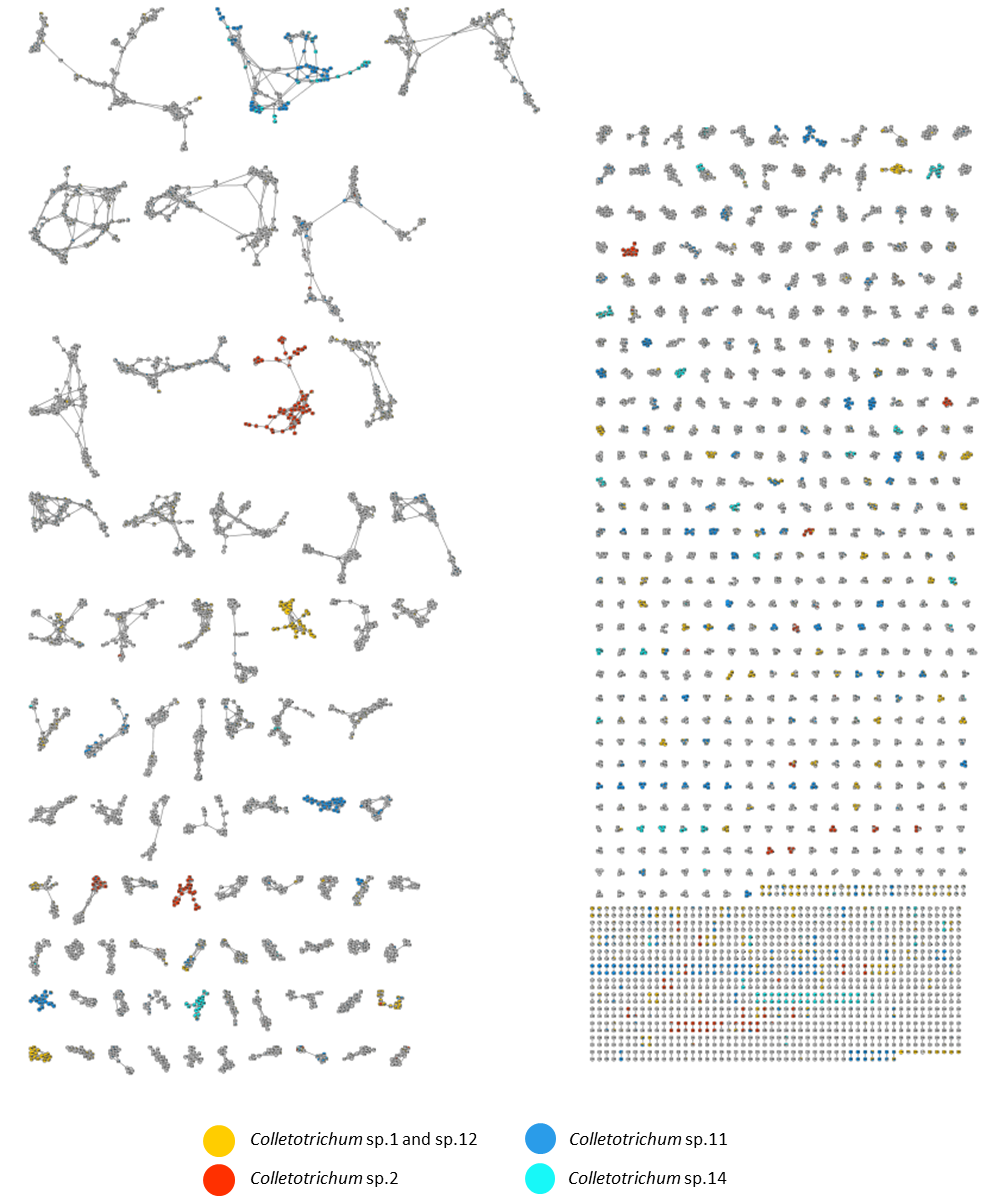


**Figure S26:** t-SNE representation of the MN of the 42 EtOAc extracts from *Colletotrichum* strains. Relative quantification of each ion within the extracts are represented as a XIC area-dependent pie-chart drawing. Mapping of the nodes are done depending the clusterisation in species via the MALDI-TOF MS fingerprinting


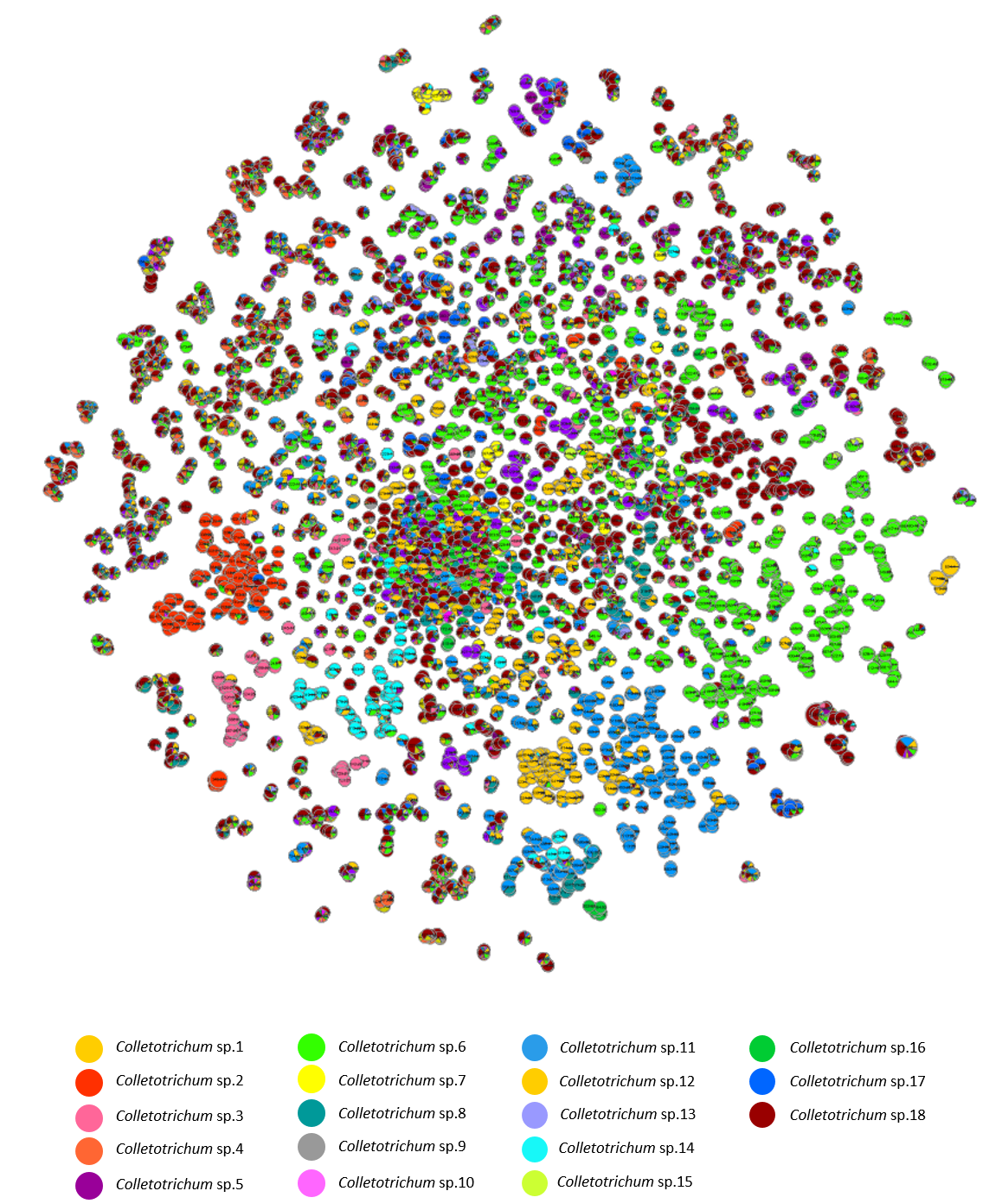


**Figure S27:** t-SNE representation of the Molecular network of the 42 EtOAc extracts from *Colletotrichum* strains. Relative quantification of each ion within the extracts are represented as a XIC area-dependent pie-chart drawing. Mapping of the nodes are done depending the cytotoxicity of the species. Nodes in grey are from extracts with a viability on MRC-5 cells > 30%.


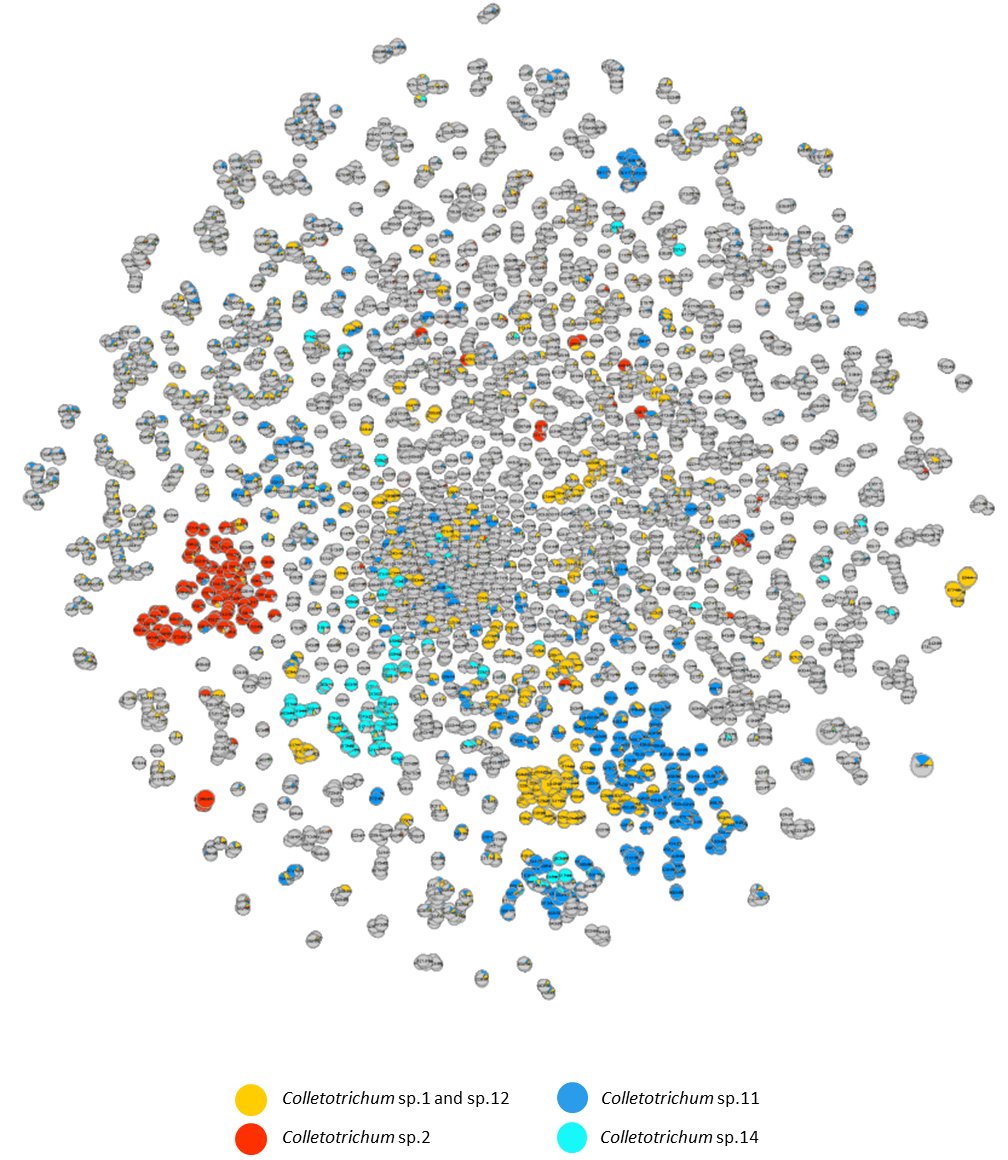


**Figure S28:** ^1^H-NMR (500 MHz in DMSO-*d_6_*) of Cyclo-(Phe-Leu-Leu-Leu-Val) (**1**)


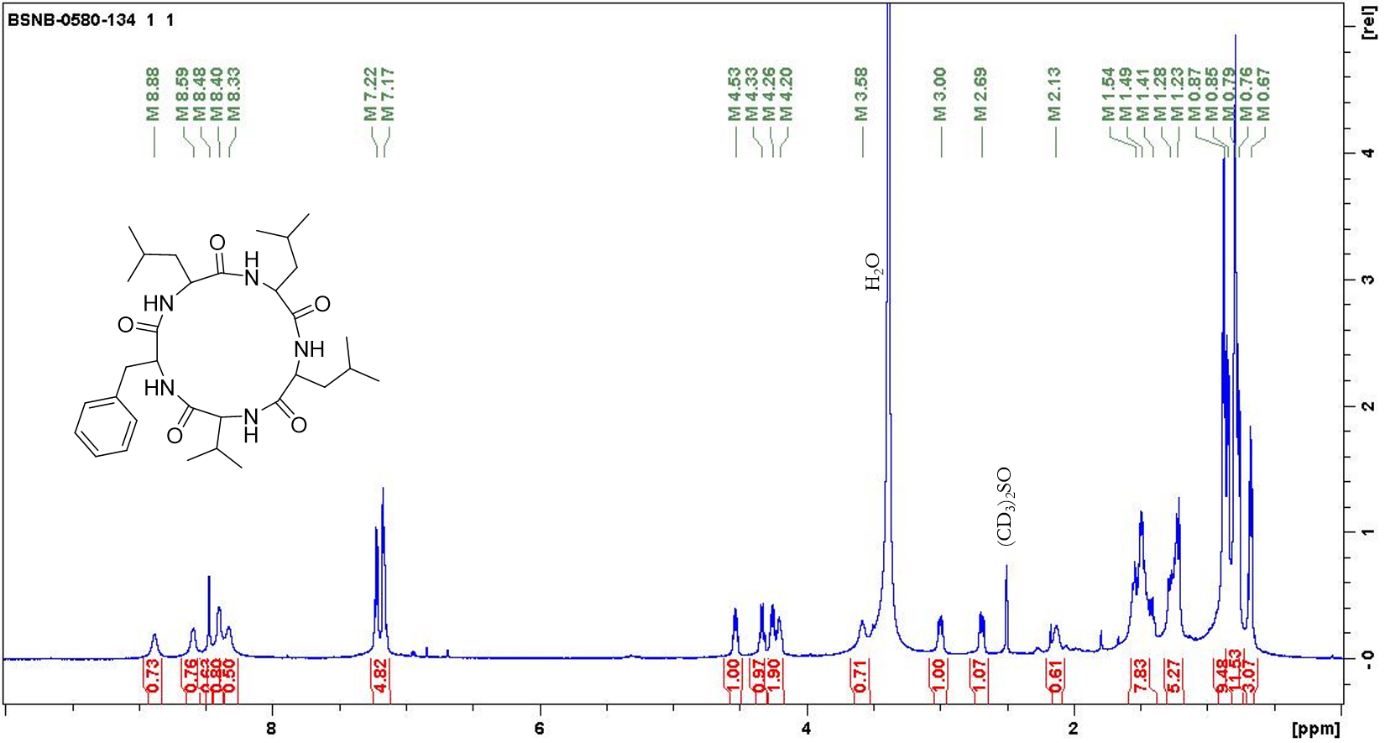


**Figure S29:** MS spectrum of Cyclo-(Phe-Leu-Leu-Leu-Val) (**1**)


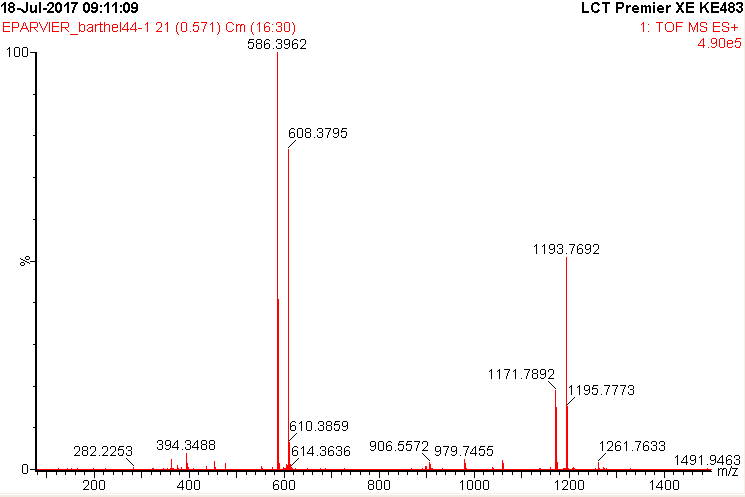


**Figure S30:** ^1^H-NMR (500 MHz in DMSO-*d_6_*) of Cyclo-(Phe-Leu-Leu-Leu-Leu) (**2**)


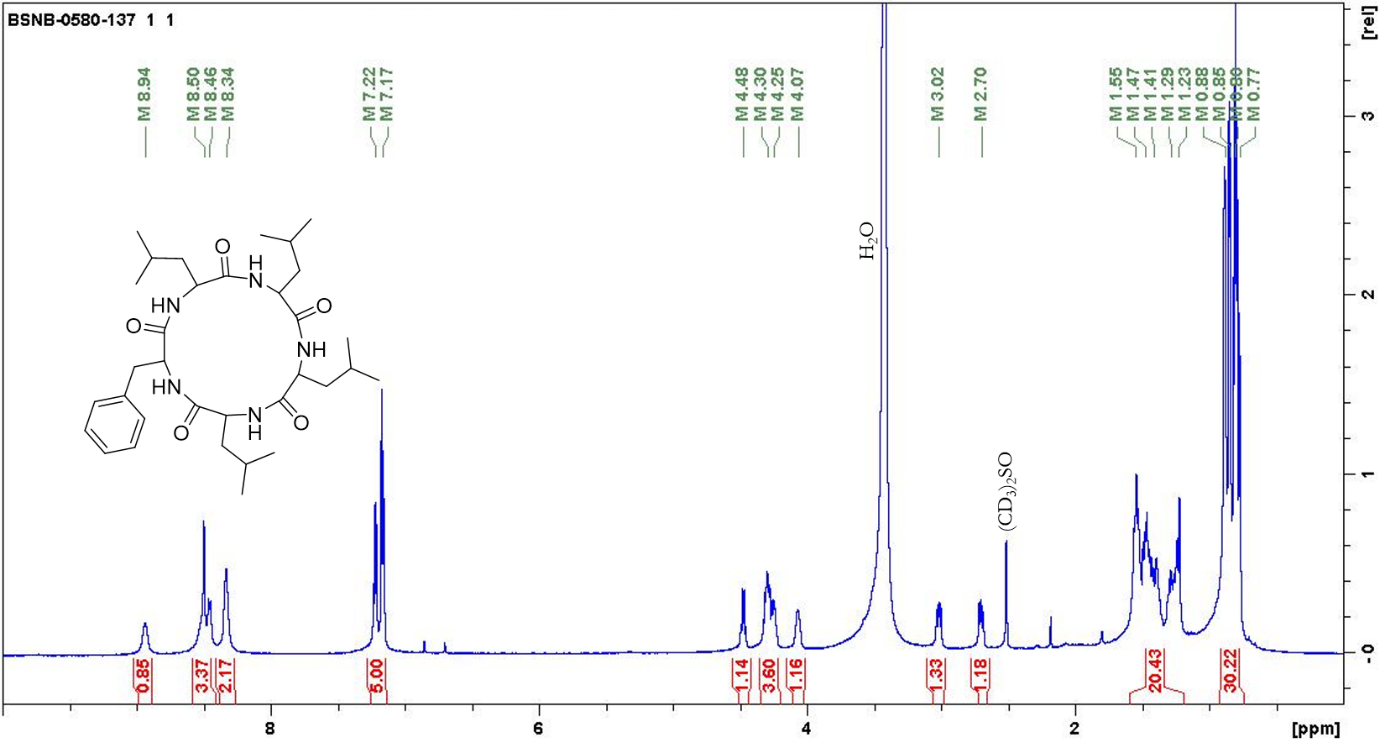


**Figure S31:** MS spectrum of Cyclo-(Phe-Leu-Leu-Leu-Leu) (**2**)


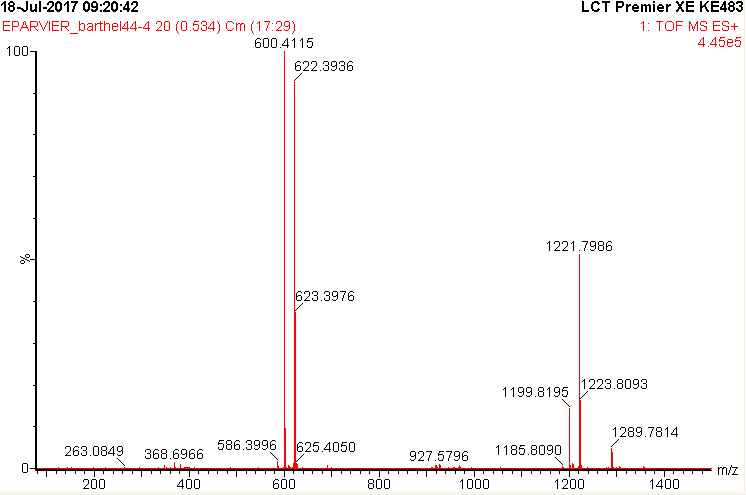


**Figure S32:** ^1^H-NMR (500 MHz in DMSO-*d_6_*) of Cyclo-(Phe-Leu-Leu-Leu-Ile) (**3**)


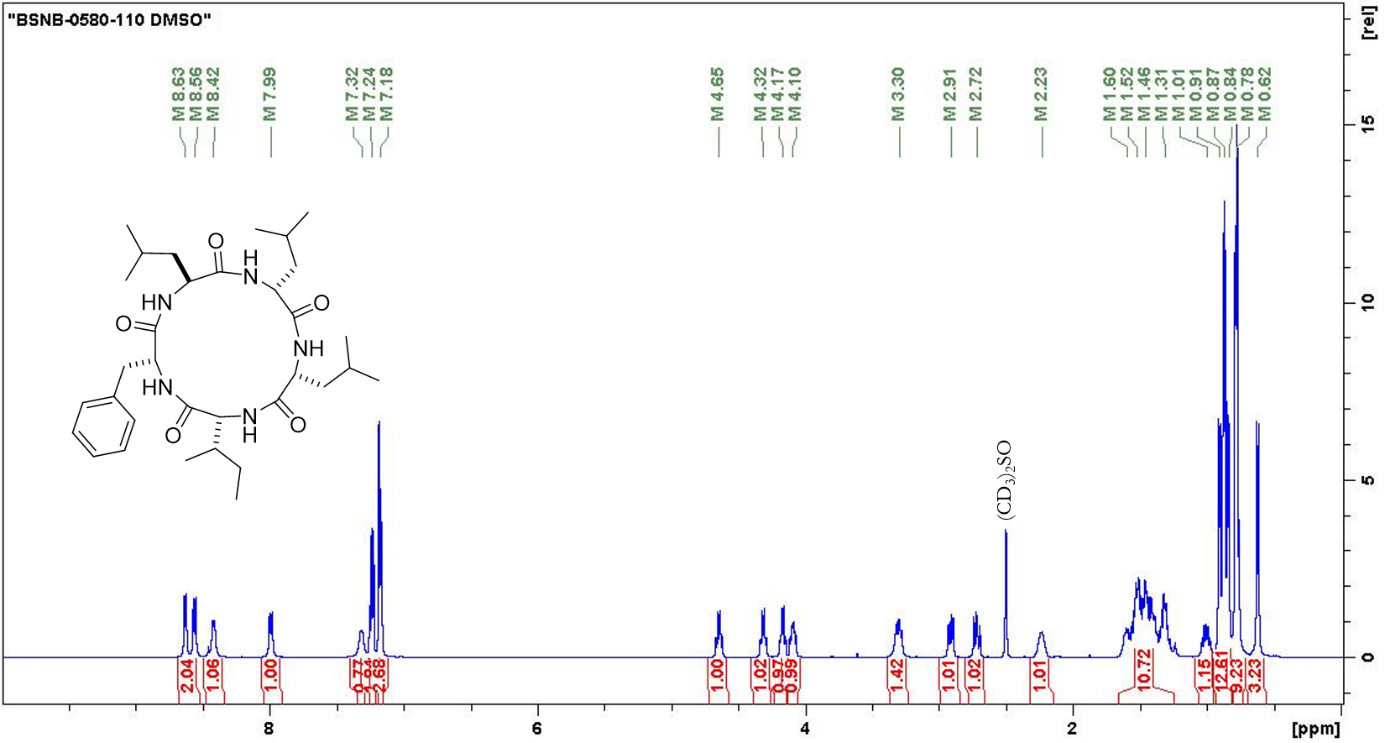


**Figure S33:** MS spectrum of Cyclo-(Phe-Leu-Leu-Leu-Ile) (**3**)

**
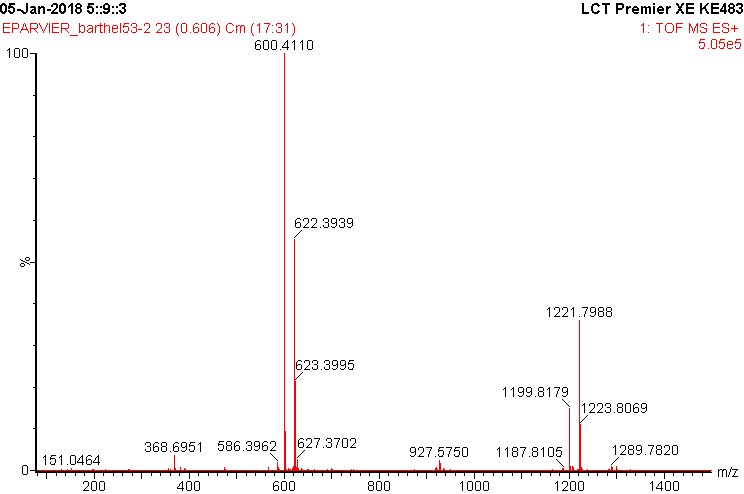
**

**Figure 34:** MS/MS fragmentation of Cyclo-(Phe-Leu-Leu-Leu-Ile) (**3**)

**Figure 35:** MS/MS fragmentation of Cyclo-(Phe-Leu-Leu-Leu-Val) (**1**)

**Figure 36:** MS/MS fragmentation of cyclopeptides with oxidized methionine amino acid


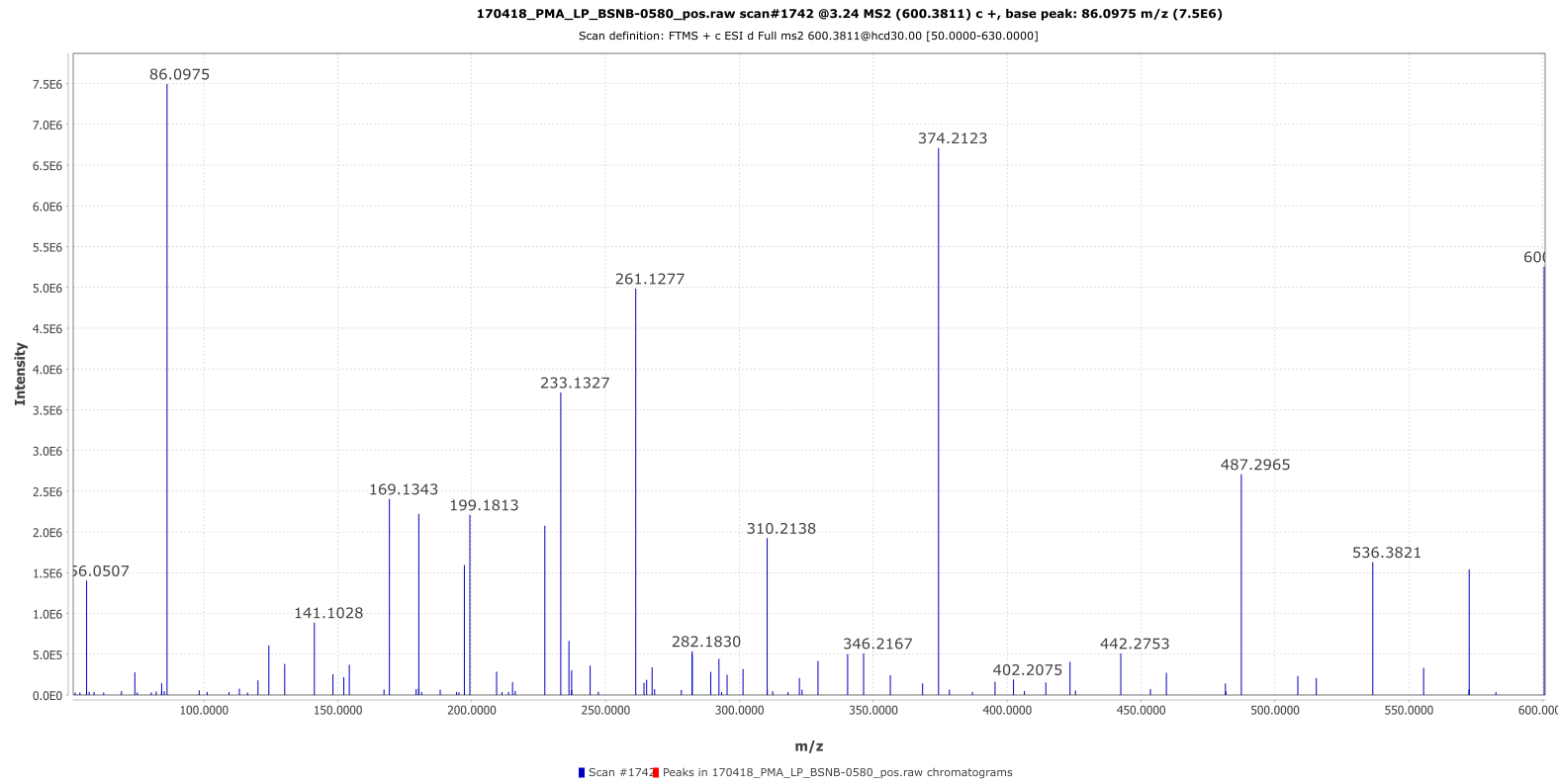


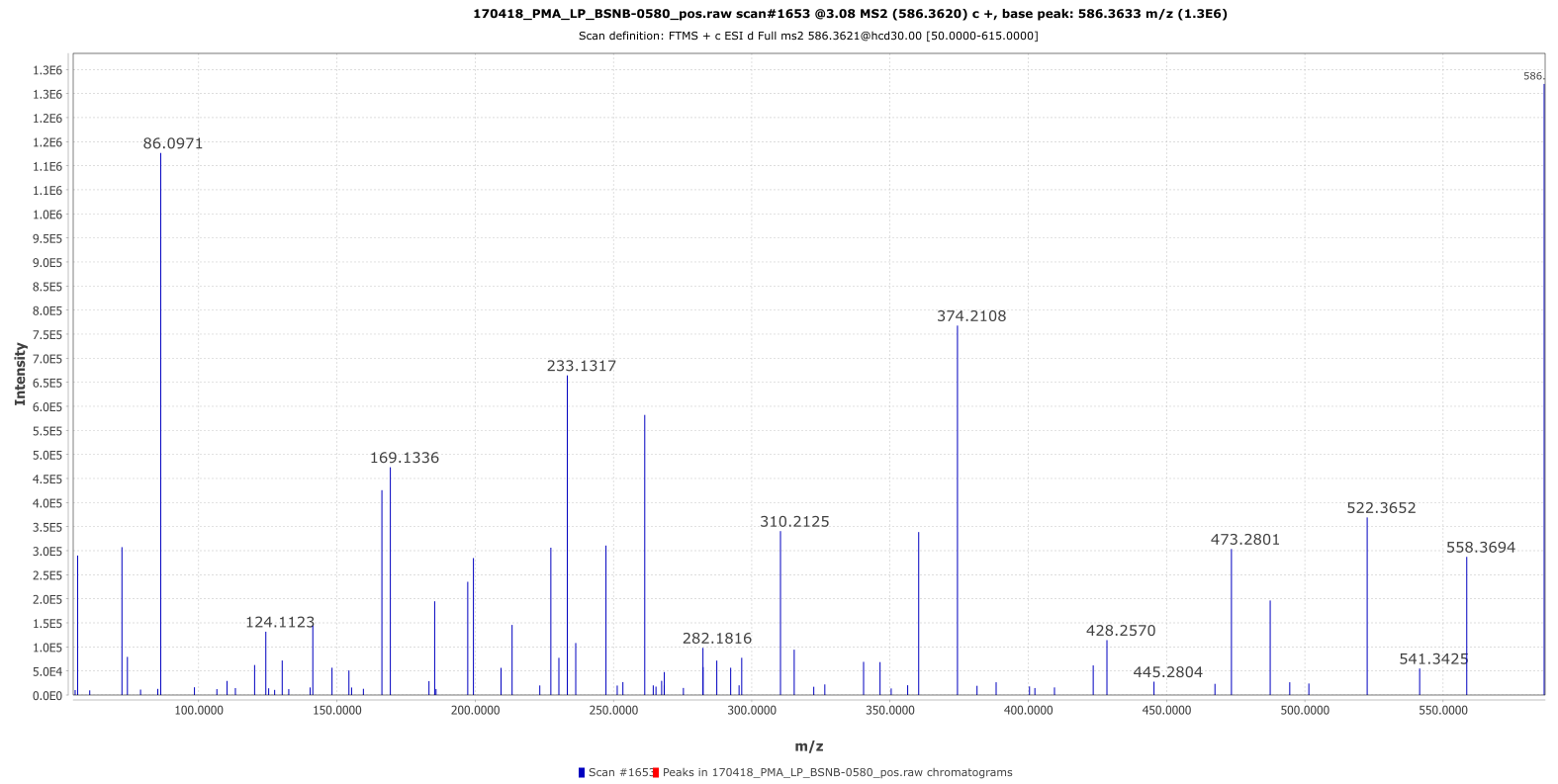


**Figure 37:** MS/MS fragmentation of putative cyclo-(Leu-Leu-Leu-Leu-Leu)

**Figure 38:** MS/MS fragmentation of putative cyclo-(Leu-Leu-Leu-Val-Val)

**Figure 39:** MS/MS fragmentation of putative cyclo-(Phe-Phe-Leu-Leu-Val)

**Figure S40:** ^1^H-NMR (500 MHz, CD_3_OD) of Cytochalasin D (**4**) analogue

**Figure S41:** MS spectrum of Cytochalasin D (**4**) analogue


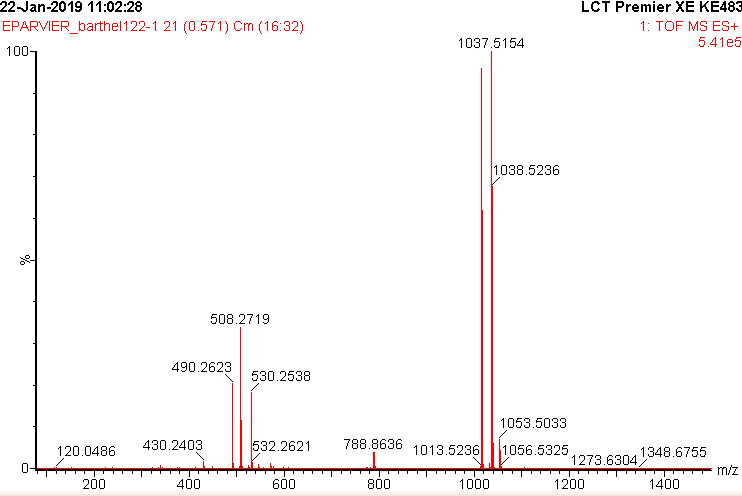


**Figure S42:** ^1^H-NMR (6MHz, CD_3_OD) of Cytochalasin C analogue (**5**)

**Figure S43:** MS spectrum of Cytochalasin C analogue (**5**)


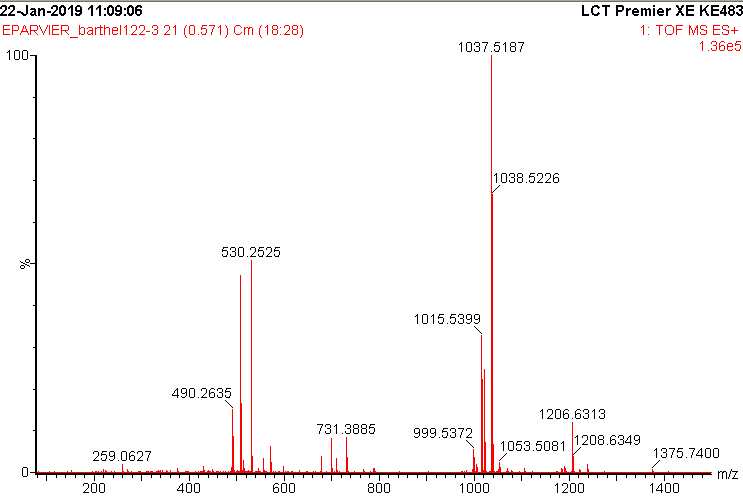


**Figure S44:** ^1^H-NMR (500 MHz, CD_3_OD) and MS spectrum of Hirsutatin A analogue (**6**)

**Figure S45:** MS spectrum of Hirsutatin A analogue (**6**)


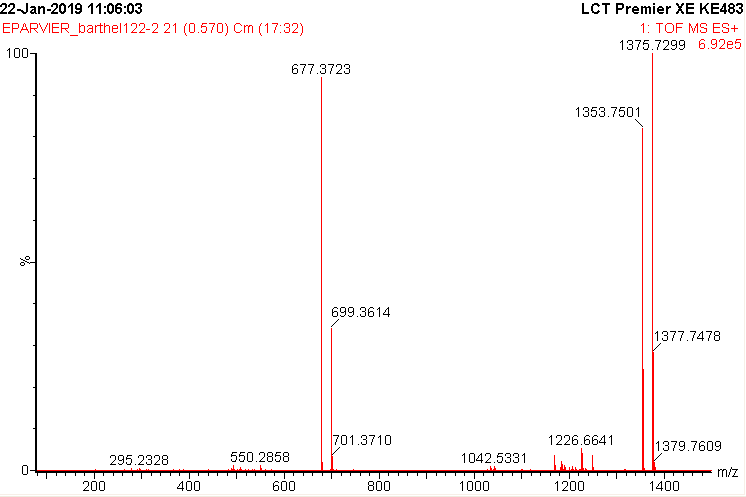


Structural characterization of compound **7**

Nine degrees of insaturations were determined from the molecular formula. The examination of the ^1^H, ^13^C and HSQC spectra allowed the identification of one methyl group at δ_H_ 1.63, five methylenes (δ_H_ [2.53, 2,83], 2.22, 2.00, 1.47 and 1.39), nine methines (δ_H_ 6.82, 5.93, 5.42, 4.34, 4.15, 4.00, 3.66 and 3.62) among which four were olefinic protons (δ_H_ 6.82, 5.93 and 5.42). The ^13^C NMR spectrum of **7** showed the presence of four quaternary carbons (δ_C_ 198.9, 175.0, 168.5 and 84.6). Their chemical shift suggested the presence of three carbonyl groups. The presence of an unsaturated chain was highlighted by the sequence of ^1^H-^1^H COSY cross-peaks for H-2/H-3/H-4/H-5/H-6/H-7/H-8/H-9/H-10 along with the HMBC correlation between H-2 and C-1 (Figure 8). Based on the coupling constant between H-2 and H-3 (*J* = 15.3 Hz), configuration of the double bond in position 2 was determined as *E*. The coupling constant could not be measured between H-8 and H-9. From the width and shape of the signal of protons H-8 and H-9 (δ_H_ 5.42) and the adjacent methylene protons H-7 (δ_H_ 2.00), the double bond in position 8 was also deduced to be *E* [Reyes, F., *et al.* Njaoamines A–F, new cytotoxic polycyclic alkaloids from the haplosclerid sponge *Reniera sp*. *Tetrahedron* **63(11)** 2432-2438 (2007).]. This chain was linked to C-2’ via an amide based on the H-2’/C-1 correlation and the chemical shift in C-2’ (δ_C_ 49.2). A portion of the carbon skeleton from C-1’ to C-4’ was deduced based on the ^1^H-^1^H COSY correlation H-2’/H-3’ and the ^1^H-^13^C HMBC correlations between H-2’ and C-1’, and between both H-3’ protons and C-1’ and C-4’. Four methines and a non-protonated carbon remained to be placed. Chemical shift of the last non-protonated carbon (δ_C_ 198.9) suggested the presence of a ketone. The sequence of ^1^H-^1^H COSY correlations H-6’/H-7’/H-8’/H-9’ provided the sequence of the four last methines. The ^1^H-^13^C HMBC correlation H-6’/C-5’, H-6’/C-4’, H-9’/C-5’ and H-9’/C-4’ suggested a six-membered ring with the ketone in C-5’ and another non-protonated carbon in C-4’. The molecular formula and the ^13^C chemical shifts indicated the presence of two epoxides in C-6’/C-7’ and in C-8’/C-9’, and a lactone ring in which C-1’ and C-4’ were connected thru an oxygen. Compound **7** was therefore a bicyclic spiro compound. The ketone was placed next to the spiro carbon C-4’ thanks to the ^1^H-^13^C correlation H-3’/C-5’.

NOE correlation H-3’a/H-9’ imposed the oxygen of the lactone and the C-8’/C-9’ epoxide to be on the same side (Figure 8). This also indicated that H-3’a and C-9’ were on the same side of the γ-lactone ring. Meanwhile, the small H-8’/H-9’ coupling suggested that the H-8’/C-8’/C-9’/H-9’ dihedral angle should be close to 90°, indicating that both epoxides were not on the same side of the cyclohexanone ring. The NOE correlation H-2’/H-3’a was much more intense than the H-2’/H-3’b correlation, suggesting that H-2’ and H-3’a were on the same side of the γ-lactone ring. Another evidence that pointed in the same direction was the strongest coupling of H-2’ with H-3’b. The γ-lactone ring should be standing in an envelope conformation in which the C-2’–nitrogen bond was pseudo-equatorial. In this conformation, both H-2’ and H-3’b protons are pseudo-axial, and their ^3^*J* coupling is stronger than the H-2’ and H-3’a coupling. In order to confirm the relative configuration of **7**, prediction of the chemical shifts was undertaken and were compared to the experimental ones using the DP4 approach.

**Table S2:** NMR data and key COSY, HMBC and NOESY correlations for the determination of Colletamide A. Correlation in brackets are weaker correlations on the spectrum.

| **Position** | **^1^H : *δ* (ppm)**  **(multiplicity, *J* (Hz))** | **^13^C : *δ* (ppm)** | **COSY (^1^H→^1^H)** | **HMBC (^1^H→^13^C)** | **NOESY (^1^H→^1^H)** |
| --- | --- | --- | --- | --- | --- |
| 1 | - | 168.5 | - | - | - |
| 2 | 5.93 (1H, d, *J* = 15.3) | 123.6 | H-3 H-4 | C-1 C-4 | H-3 H-4 |
| 3 | 6.82 (1H, dt, *J* = 15.2, 7.1) | 147.5 | H-2 H-4 | C-1 C-4 C-5 | H-2 H-4 H-5 |
| 4 | 2.22 (2H, m) | 32.9 | H-2 H-3 H-5 | C-2 C-3 C-5 C-6 | H-5 |
| 5 | 1.47 (2H, m) | 28.8 | H-4 H-6 | C-3 C-4 C-6 | H-4 |
| 6 | 1.39 (2H, m) | 30.2 | H-5 H-7 | C-5 C-7 | H-7 |
| 7 | 2.00 (2H, m) | 33.3 | H-6 H-8 | C-5 C-6 C-8 C-9 | H-10 H-5 H-6 |
| 8 | 5.42 (2H, m) | 132.2 | H-7 H-10 | C-7 C-10 | H-7 H-10 |
| 9 |  | 126.0 |  |  |  |
| 10 | 1.63 (3H, d, *J* = 4.3) | 18.1 | H-9 | C-8 C-9 |  |
| 1’ | - | 175.0 | - | - | - |
| 2’ | 4.34 (1H, t, *J* = 10.1) | 49.2 | H-3’ | C-1 C-1’ C-3’ | H-3’a (H-3’b) |
| 3’ a  3’ b | 2.83 (1H, dd, *J* = 13.7, 9.5)  2.53 (1H, dd, *J* = 13.7, 11.0) | 34.2 | H-2’ (H-6’)  H-9’ (H-2’) | C-1’ C-2’ C-4’ C-5’ C-9’ | H-2’ (H-6’)  H-9’ (H-2’) |
| 4’ | - | 84.6 | - | - | - |
| 5’ | - | 198.9 | - | - | - |
| 6’ | 3.62 (1H, d, *J* = 4.3) | 56.5 | H-7’ | C-4’ C-5’ C-7’ | H-7’ H-3’a |
| 7’ | 4.15 (1H, dd, *J* = 3.6, 1.8) | 59.0 | H-6’ H-8’ | C-8’ C-9’ | H-6’ |
| 8’ | 4.00 (1H, dd, *J* = 3.9, 2.0) | 53.8 | H-7’ H-9’ | C-6’ C-7’ | H-9’ (H-3’b) |
| 9’ | 3.66 (1H, d, *J* = 4.3) | 57.2 | H-8’ | C-4’ C-5’ C-8’ | H-8’ H-3’b (H-3’a) |

**Figure S46:** ^1^H-NMR (500 MHz, CD_3_OD) of Colletamide A (**7**)

**Figure S47:** ^13^C-NMR (500 MHz, CD_3_OD) spectrum of Colletamide A (**7**)


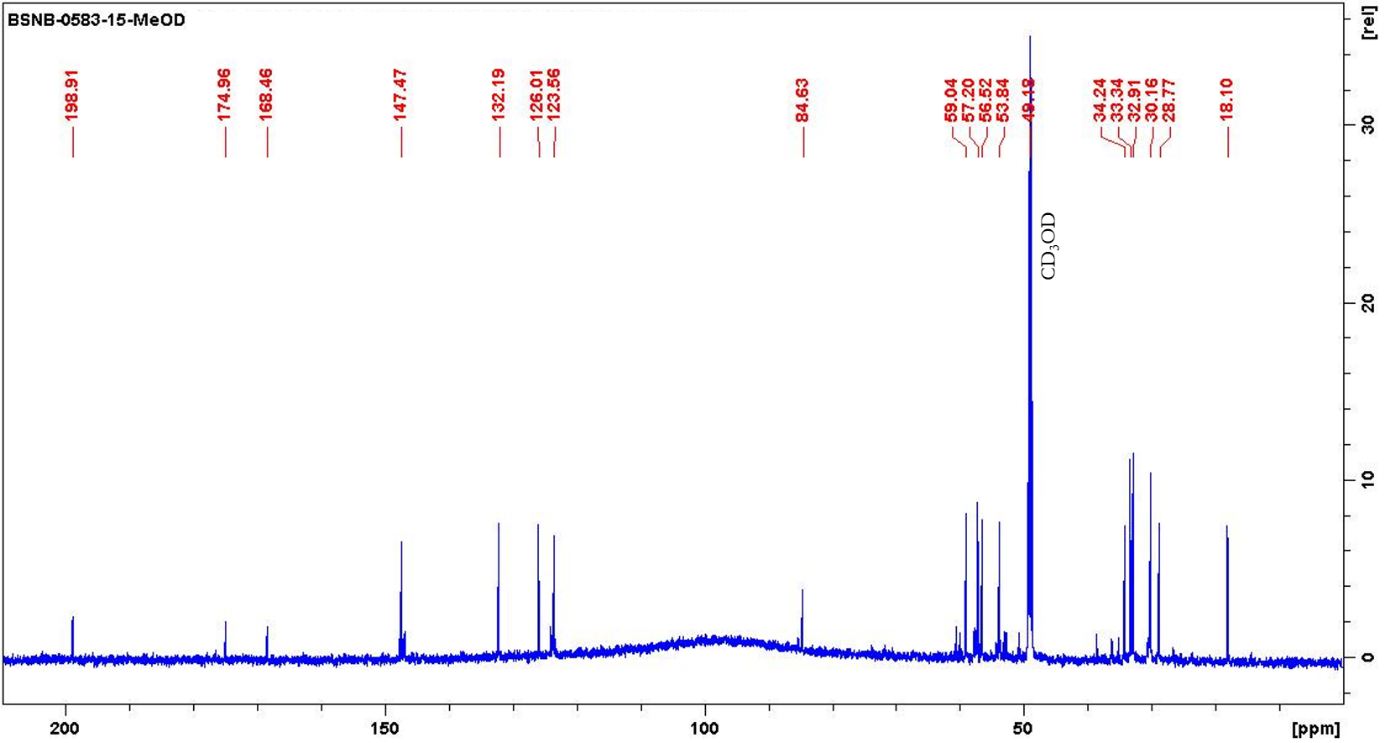


**Figure S48:** ^1^H-^1^H correlations COSY (500 MHz, CD_3_OD) of Colletamide A (**7**)


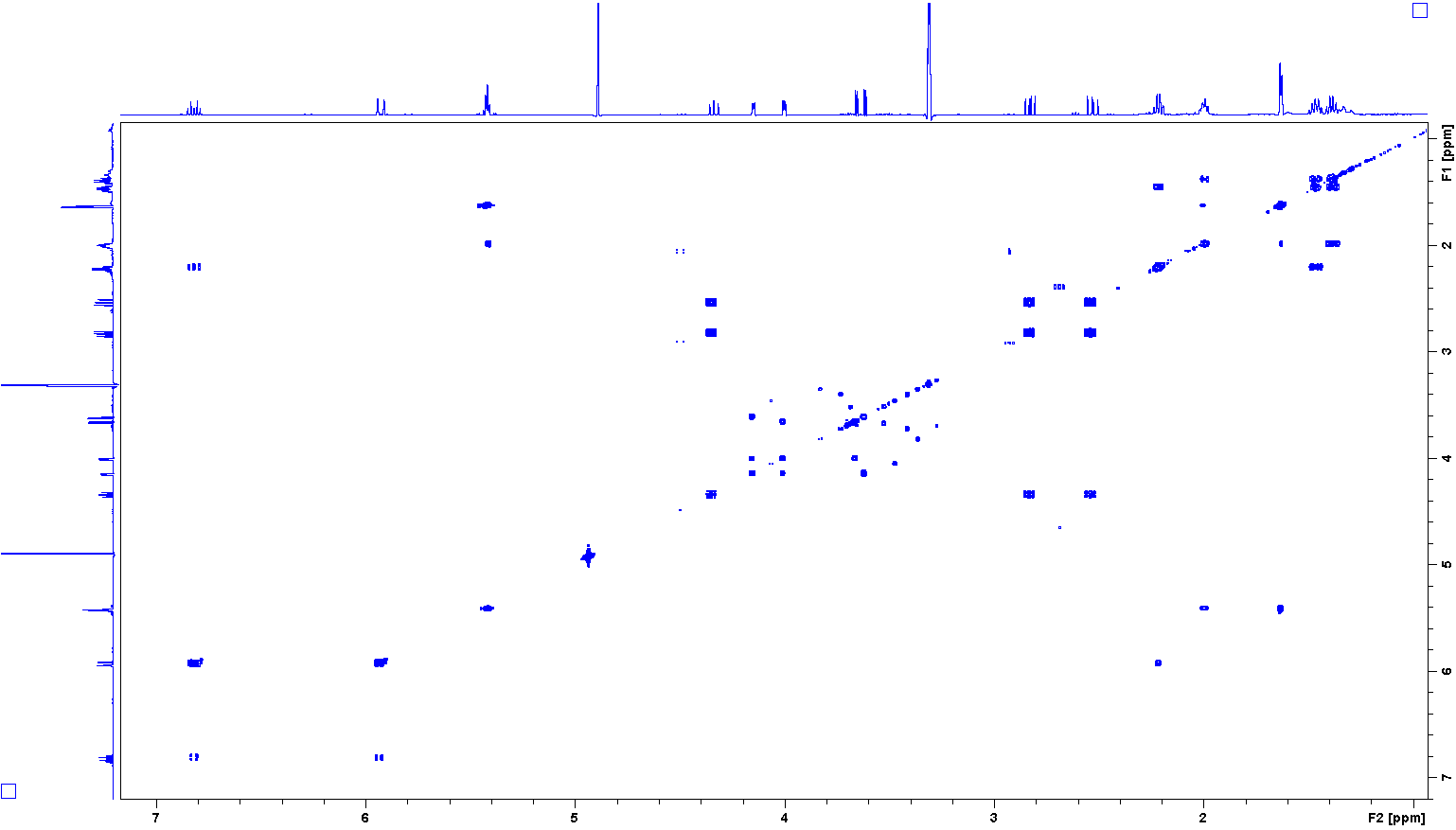


**Figure S49:** ^1^H-^13^C correlations HSQC (500 MHz, CD_3_OD) of Colletamide A (**7**)


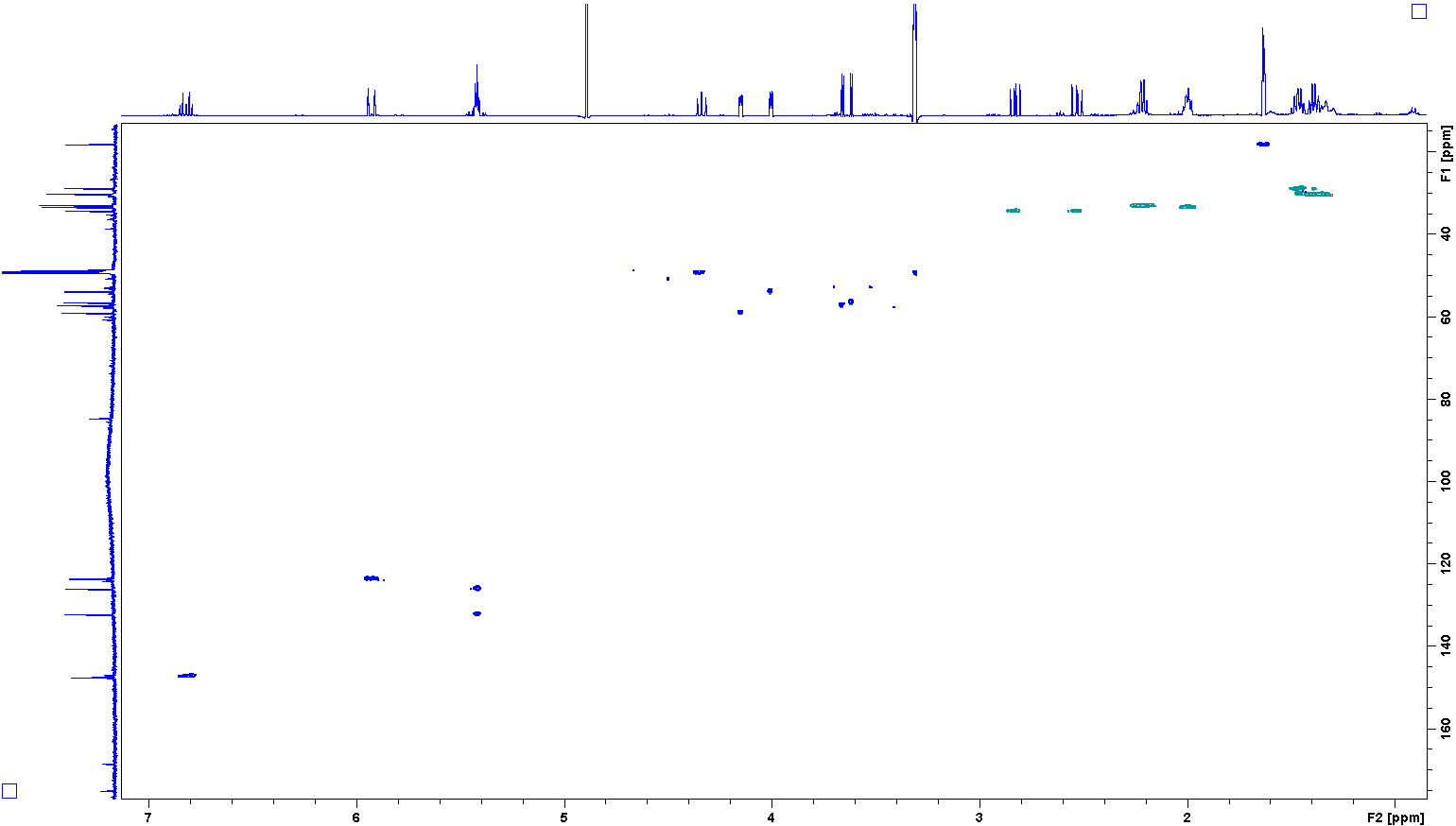


**Figure S50:** ^1^H-^13^C correlations HMBC (500 MHz, CD_3_OD) of Colletamide A (**7**)


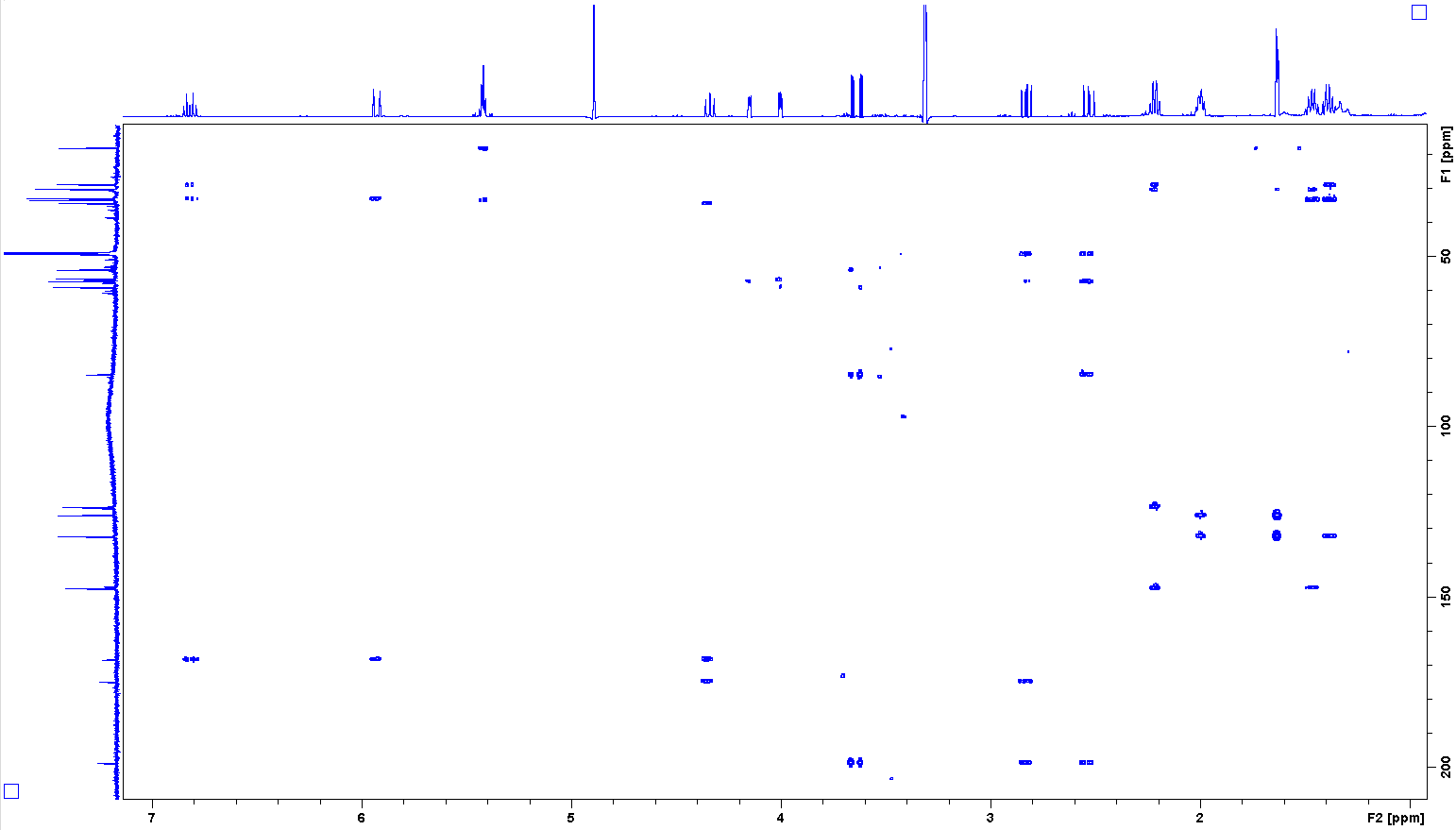


**Figure S51:** ^1^H-^1^H correlations NOESY (500 MHz, CD_3_OD) of Colletamide A (**7**)


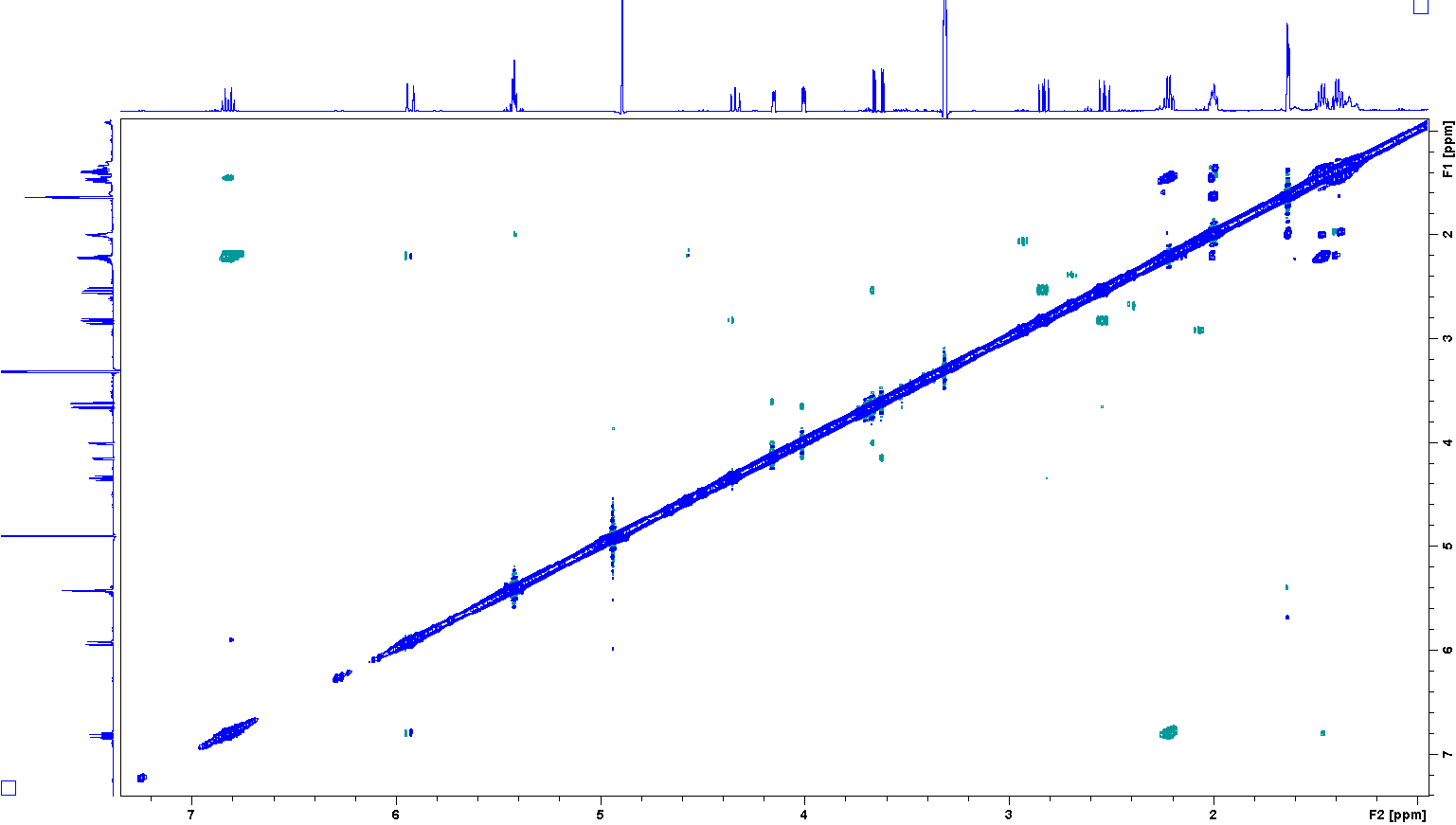


**Figure S52:** MS spectrum of Colletamide A (**7**)


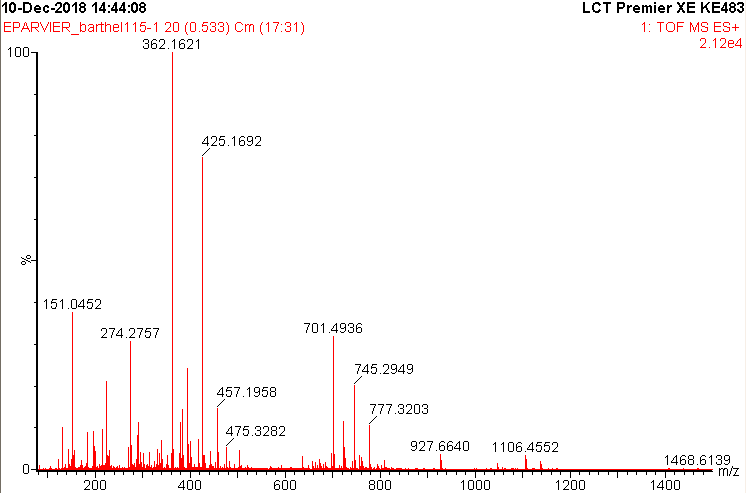


**Figure S53**. COSY (bold) and key HMBC (black arrows) correlations in colletamides A-D. NOE correlations are represented as coloured arrows: plain arrows correspond to strong correlations and dashed arrows to weak correlations.

**Figure S54**: Comparison of the predicted ECD spectrum with the experimental spectrum of Colletamide A (**7**)


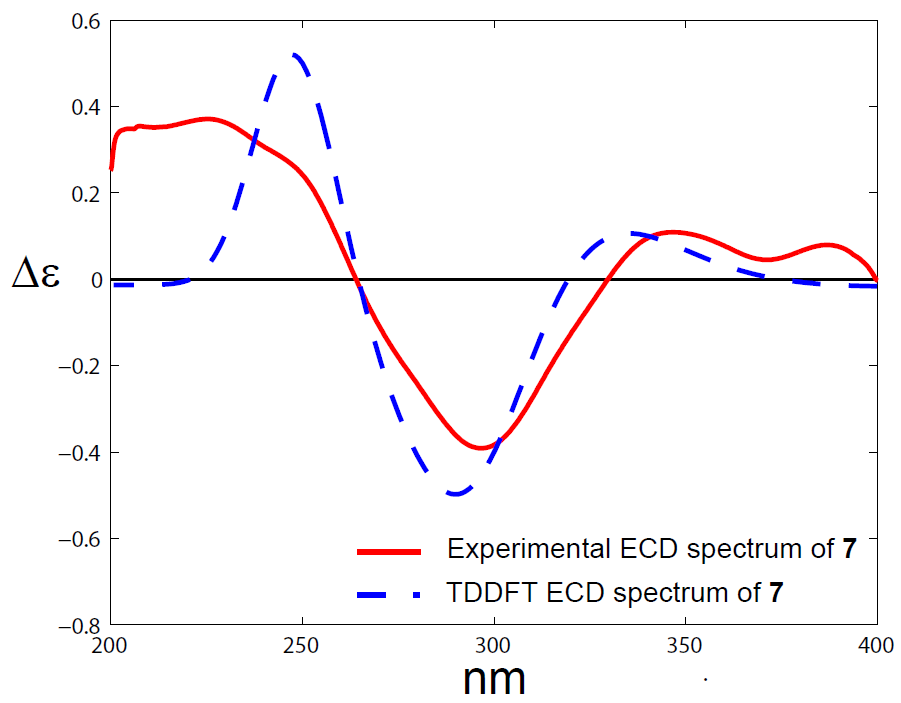


Structural characterization of compound **8**

Compound **8** formula was determined as C_22_H_29_NO_4_ based on the protonated molecule at *m/z* 372.2182 [M+H]^+^ in the ESI^+^-HRMS analysis (calcd for C_22_H_30_NO_4_^+^, 372.2169, mass error 3.49 ppm). Examination of ^1^H, ^13^C, HSQC spectra, ^1^H-^1^H COSY and ^1^H-^13^C HMBC correlations indicated the presence of the same (2*E*,8*E*)-deca-2,8-dienamide chain as in compound **7** (Figure S53). For the rest of the molecule, examination of ^1^H, ^13^C and HSQC spectra showed the presence of two methylenes (δ_H_ 2.99, 2.85, 2.41 and 2.17), three methines (δ_H_ 4.71, 4.40, 4.32), one non-protonated carbon (δ_C_ 178.4), and a phenyl ring (δ_H_ 7.26, 7.25, 7.18 and δ_C_ 139.0, 130.2, 129.5 and 127.6). The decadienamide chain was linked to C-5’ following the ^1^H-^13^C HMBC correlation H-5’/C-1. ^1^H-^1^H COSY correlations allowed the elucidation of the sequence between H6’/H-5’/H-4’/H’3’/H’2’. The presence of a phenyl group was confirmed with COSY and HMBC correlations. This phenyl was linked to a methylene in position 6’ thanks to correlations H-5’/C-7’ and H-6’/C-7’. A lactone moiety was deduced from the ^1^H-^13^C correlation H-4’/C-1’ and H-2’/C-1’ and from the chemical shift of C-4’ (δ_C_ 80.7). Molecular formula and chemical shift of C-2’ (δ_C_ 68.0) confirmed the presence of a hydroxyl on C-2’. The absolute configuration of **8** was determined by comparison of the predicted and experimental NMR chemical shift (Figure S62). The (2*E*,8*E*)-*N*-[(*R*)-1-((2*S*,4*S*)-4-hydroxy-5-oxotetrahydrofuran-2-yl)-2-phenylethyl]deca-2,8-dienamide **(8)** was named colletamide B.

**Table S3:** NMR data and key COSY, HMBC and NOESY correlations for the determination of Colletamide B. Correlation in brackets are weaker correlations on the spectrum.

| **Position** | **^1^H : *δ* (ppm)**  **(multiplicity, *J* (Hz))** | **^13^C : *δ* (ppm)** | **COSY (^1^H→^1^H)** | **HMBC (^1^H→^13^C)** | **NOESY (^1^H→^1^H)** |
| --- | --- | --- | --- | --- | --- |
| 1 | - | 169.0 | - | - | - |
| 2 | 5.88 (1H, d, *J* = 15.1) | 124.0 | H-3 H-4 | C-1 C-3 | H-3 H-4 (H-2’) |
| 3 | 6.72 (1H, dt, *J* = 15.1, 7.0) | 146.8 | H-2 H-4 | C-1 C-2 C-4 C-5 | H-2 H-4 H-5 |
| 4 | 2.16 (2H, m) | 32.9 | H-2 H-3 H-5 | C-2 C-3 C-5 C-6 | H-2 H-3 H-5 |
| 5 | 1.43 (2H, m) | 28.8 | H-4 H-6 | C-3 C-6 C-7 |  |
| 6 | 1.35 (2H, m) | 30.1 | H-7 | C-5 C-7 C-8 |  |
| 7 | 1.98 (2H, m) | 33.3 | H-6 H-8 H-9 H-10 | C-6 C-8 C-9 |  |
| 8 | 5.41 (2H, m) | 132.2 | H-7 H-10 | C-7 C-10 | H-7 H-10 |
| 9 |  | 126.0 |  |  |  |
| 10 | 1.63 (3H, d, *J* = 4.1) | 18.1 | H-8 H-9 | C-8 C-9 |  |
| 1’ | - | 178.4 | - | - | - |
| 2’ | 4.32 (1H, dd, *J* = 8.2, 7.1) | 68.0 | H-3’ | C-1’ C-3’ C-4’ | H-3’ (H-2) |
| 3’ a  3’ b | 2.41 (1H, ddd, *J* = 13.7, 8.6, 4.3)  2.17 (1H, m) | 34.6 | H-2’ H-4’ | C-1’ C-2’ C-4’ C-5’ | H-2’ H-4’ H-5’ |
| 4’ | 4.71 (1H, ddd, *J* = 8.3, 4.3, 2.9) | 80.7 | H-3’ H-5’ | C-1’ C-2’ C-3’ C-5’ C-6’ | H-3’ H-5’ H-6’ |
| 5’ | 4.40 (1H, ddd, *J* = 9.3, 7.3, 2.8) | 54.7 | H-4’ H-6’ | C-1 C-3’ C-6’ C-7’ | H-3’ H-4’ H-6’ |
| 6’ | 2.99 (1H, dd, *J* = 14.0, 6.1)  2.85 (1H, dd, *J* = 14.0, 9.3) | 38.6 | H-5’ | C-4’ C-5’ C-7’ C-8’ | H-4’ H-5’ H-6’ |
| 7’ | - | 139.0 | - | - | - |
| 8’ | 7.25 (2H, m) | 130.2 | H-9’ | C-8’ C-10’ |  |
| 9’ | 7.26 (2H, m) | 129.5 | H-8’ H-10’ | C-7’ C-9’ |  |
| 10’ | 7.18 (1H, m) | 127.6 | H-9’ | C-8’ |  |

**Figure S55:** ^1^H-NMR (500 MHz, CD_3_OD) of Colletamide B **(8)**

**Figure S56:** ^13^C-NMR (500 MHz, CD_3_OD) spectrum of Colletamide B **(8)**


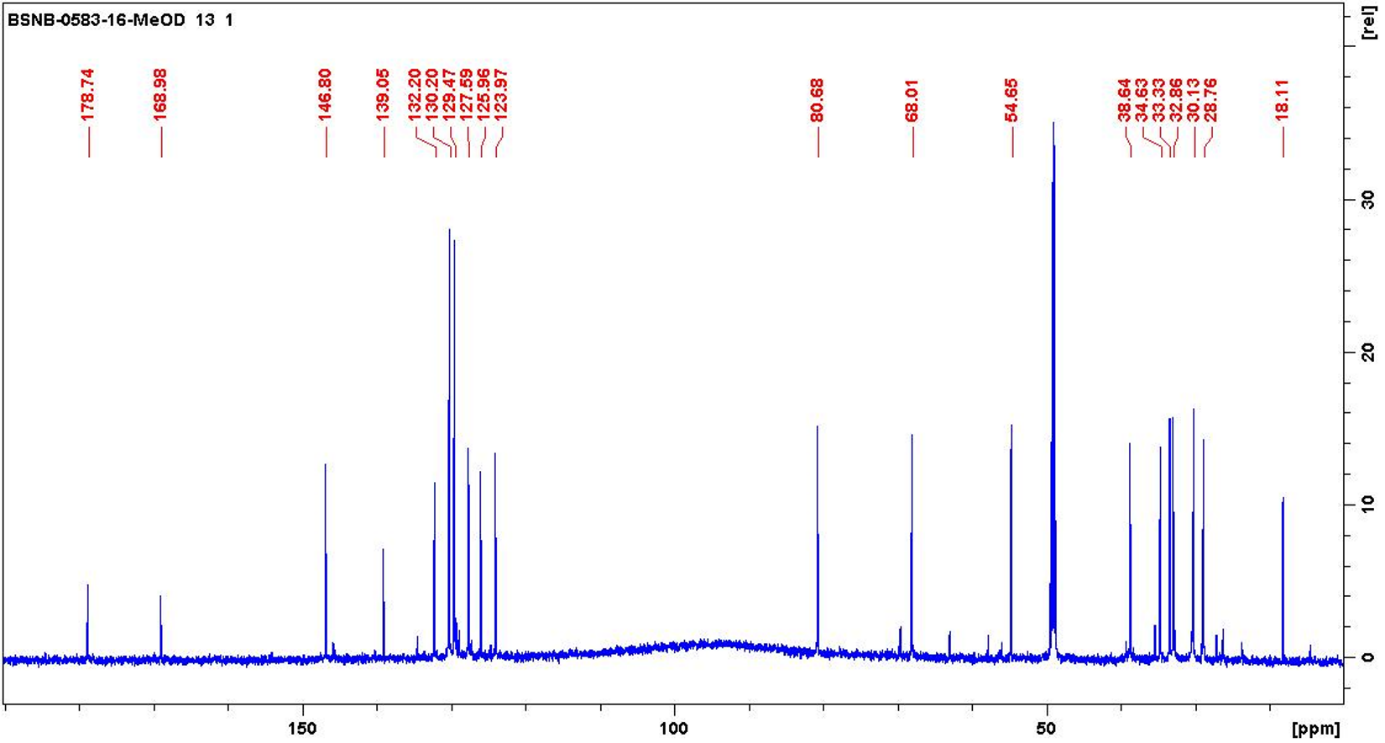


**Figure S57:** ^1^H-^1^H correlations COSY (500 MHz, CD_3_OD) of Colletamide B (**8**)


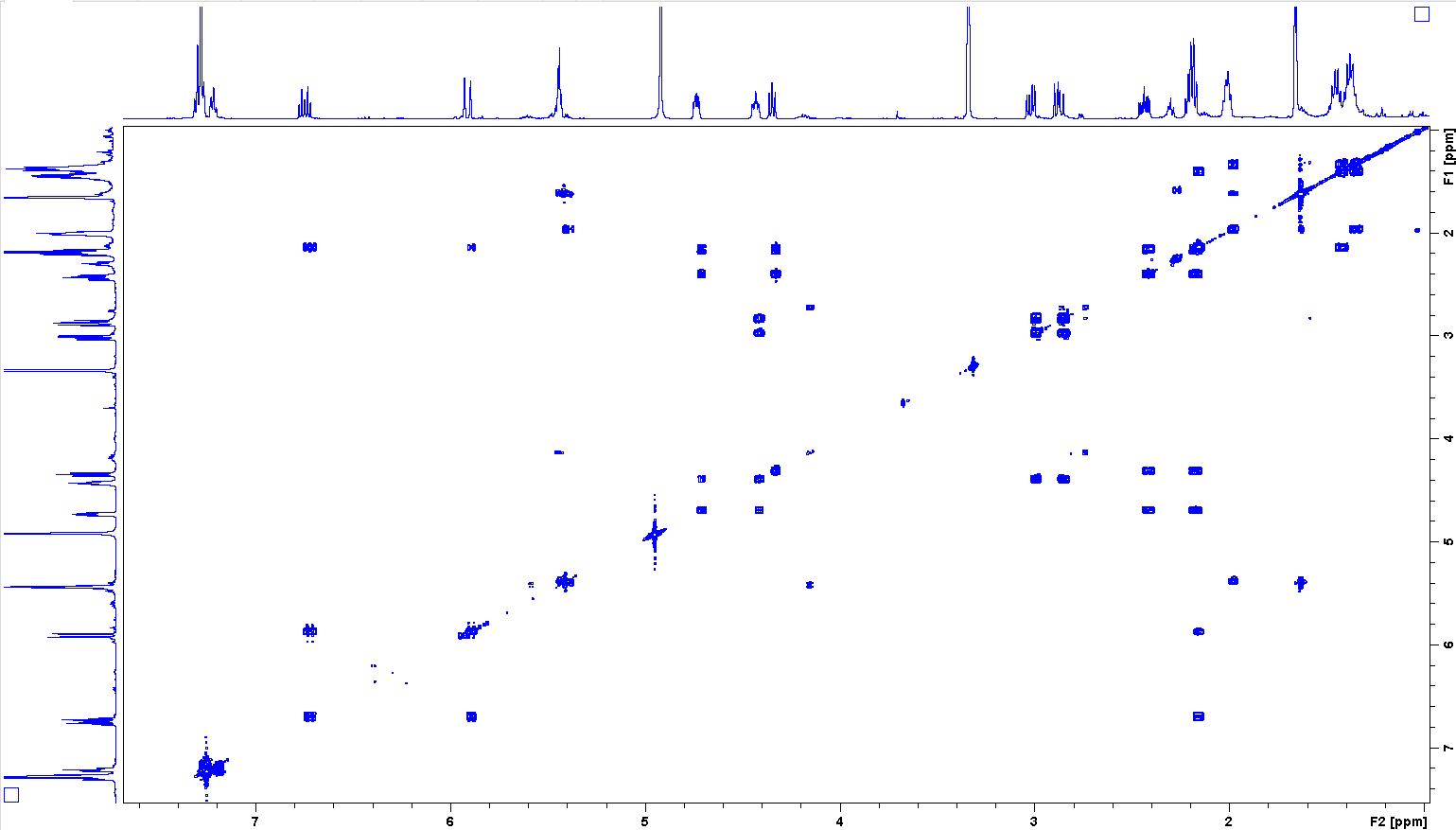


**Figure S58:** ^1^H-^13^C correlations HSQC (500 MHz, CD_3_OD) of Colletamide B (**8**)


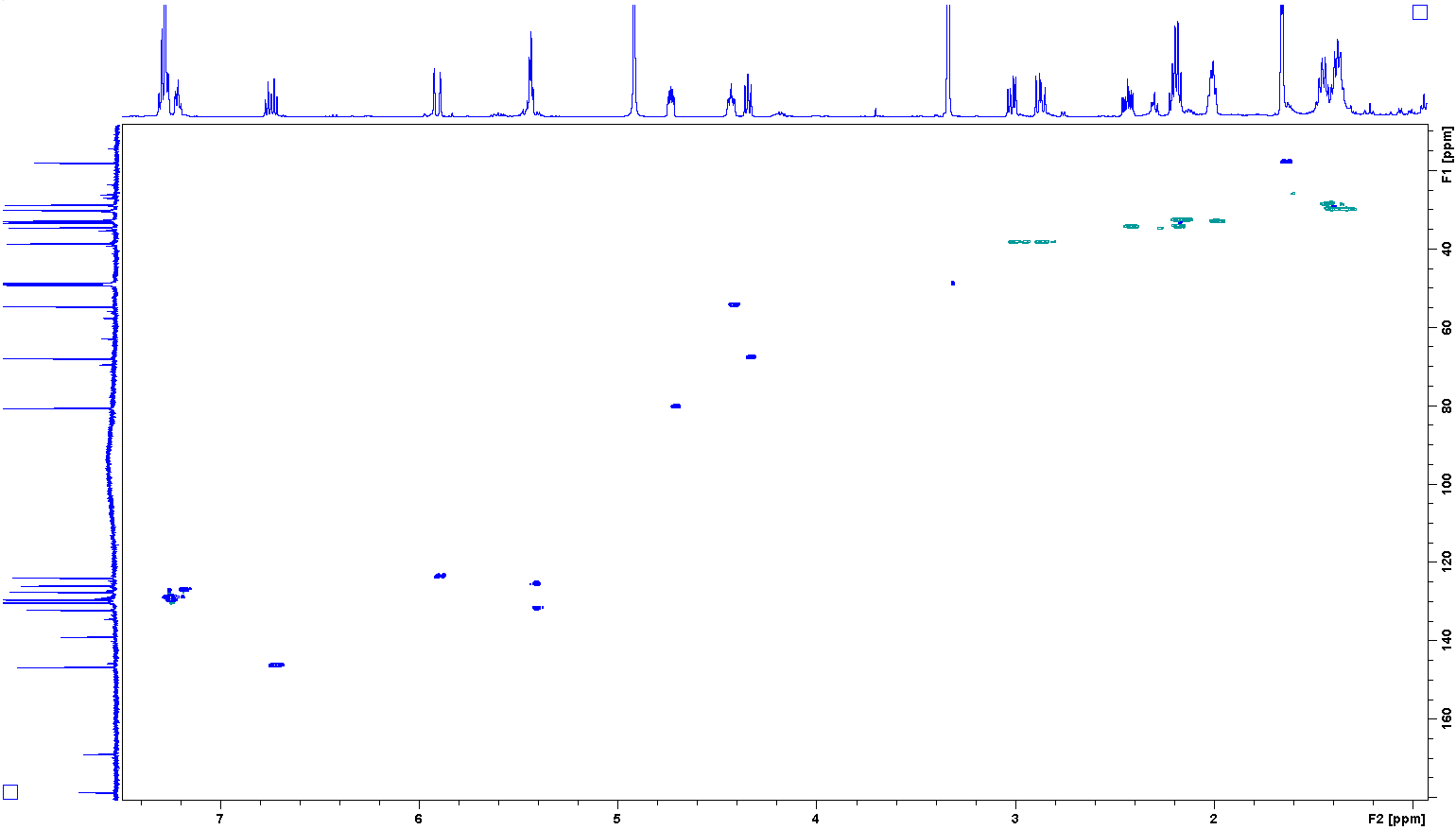


**Figure S59:** ^1^H-^13^C correlations HMBC (500 MHz, CD_3_OD) of Colletamide B (**8**)


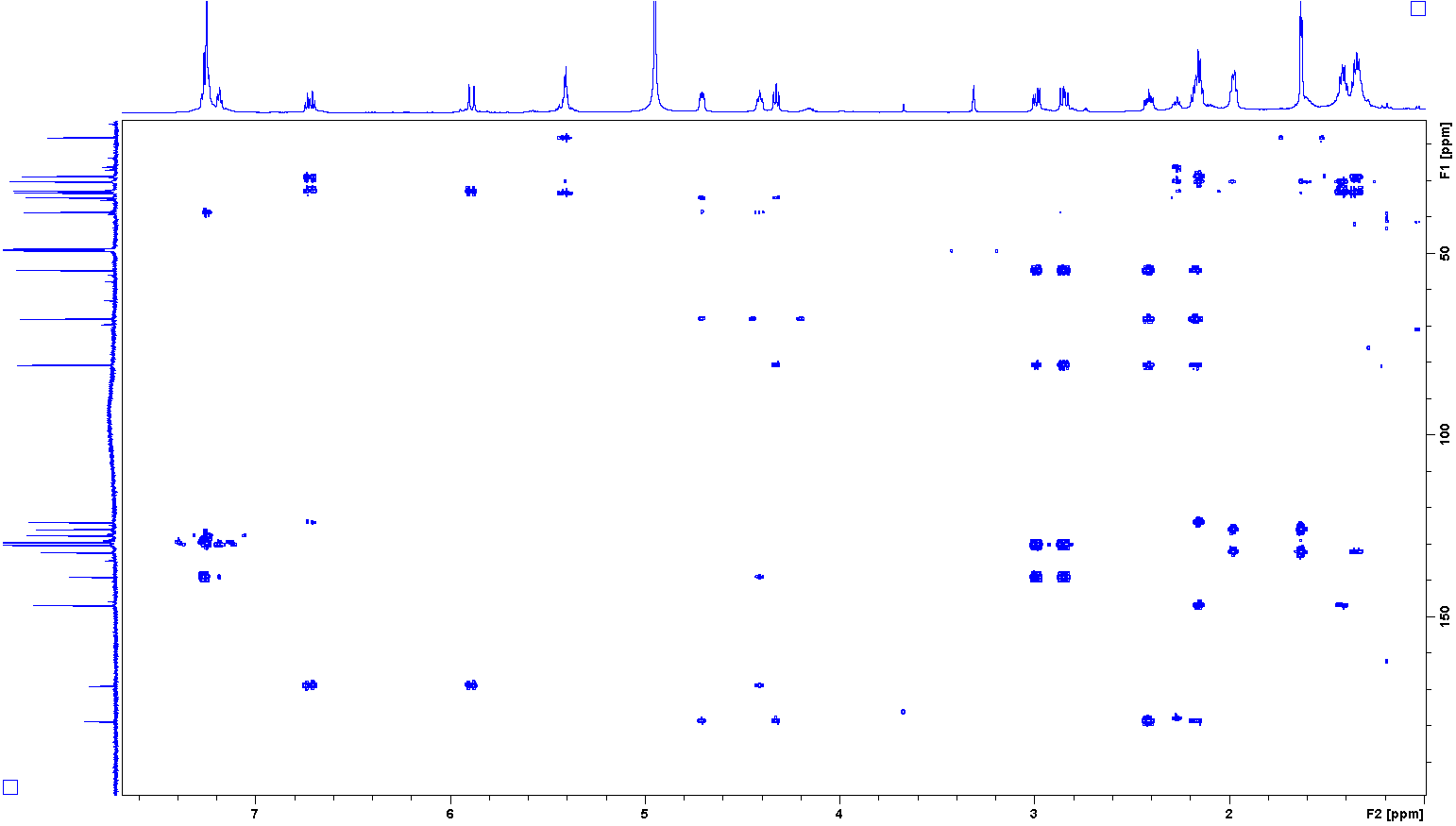


**Figure S60:** ^1^H-^1^H correlations NOESY (500 MHz, CD_3_OD) of Colletamide B (**8**)


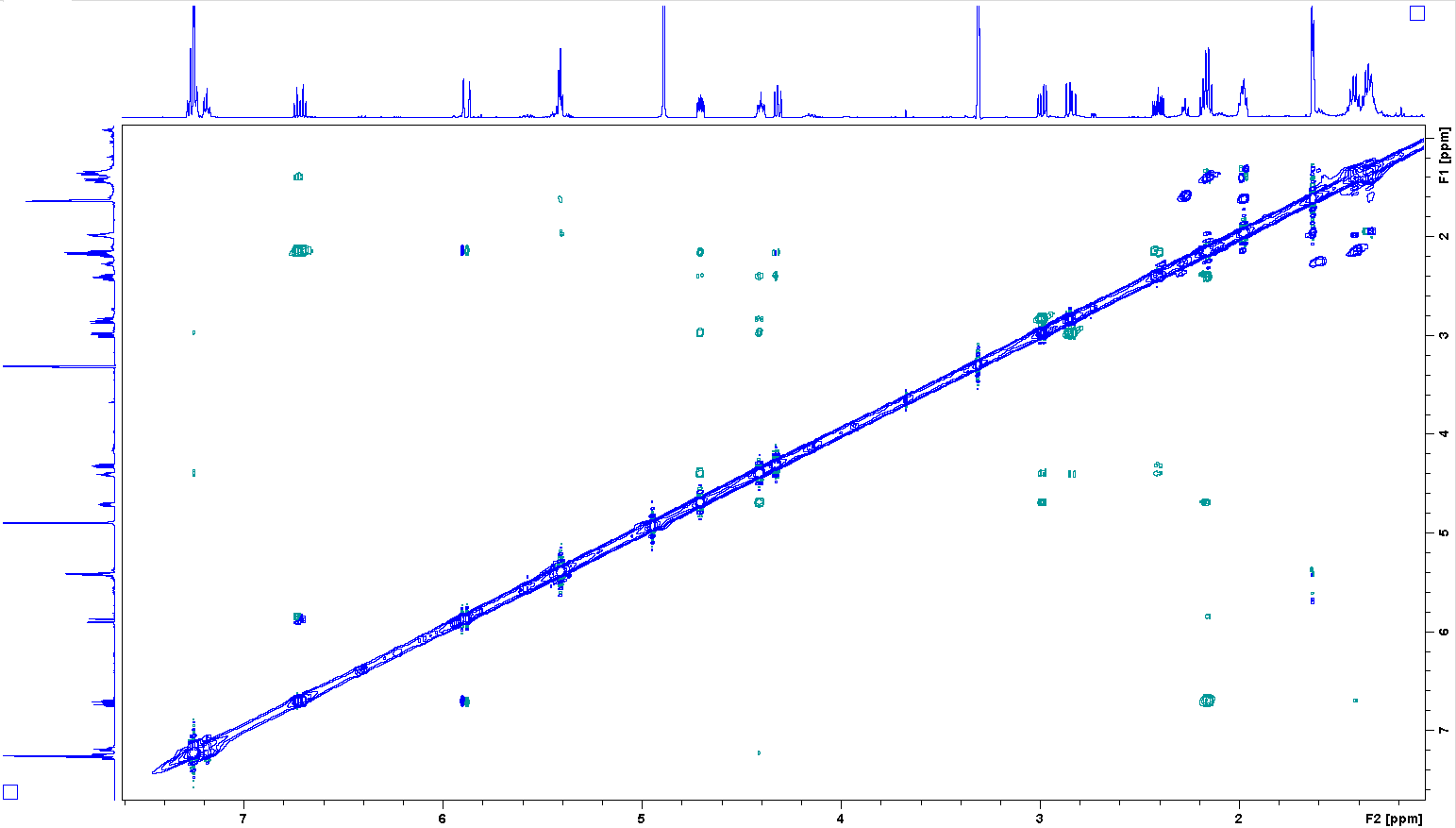


**Figure S61:** MS spectrum of Colletamide B (**8**)


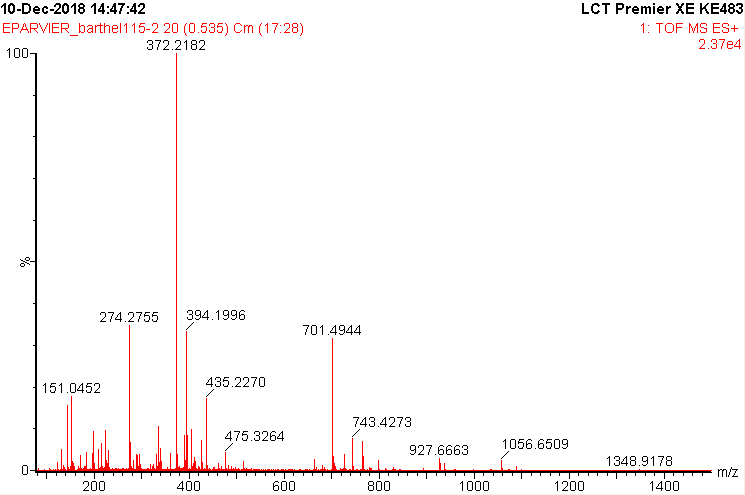


**Figure S62**: Comparison of the predicted and experimental NMR chemical shift of Colletamide B


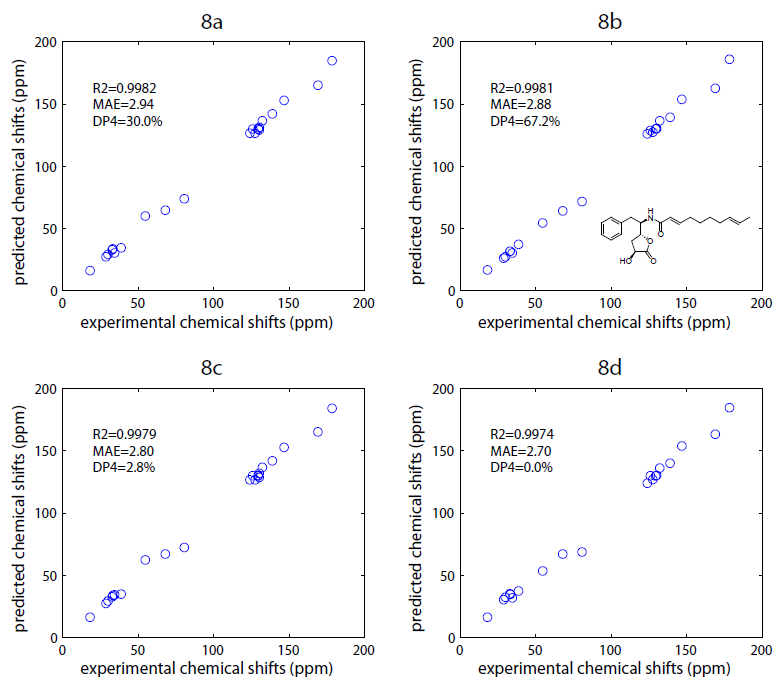


Structural characterization of compound **9**

The molecular formula of compound **(9)** was determined as C_19_H_25_NO_4_ based on the protonated molecule at *m/z* 332.1859 [M+H]^+^ in the ESI^+^-HRMS analysis (calcd for C_19_H_26_NO_4_^+^, 332.1856, mass error 0.90 ppm). Again, examination of ^1^H, ^13^C, HSQC spectra, ^1^H-^1^H COSY and ^1^H-^13^C HMBC correlations indicated the presence of the same (2*E*,8*E*)-deca-2,8-dienamide chain as in compounds **7** and **8**. ^1^H, ^13^C and HSQC spectra showed the additional presence of one methylene (δ_H_ 3.21 and 2.98), five methines (δ_H_ 7.08, 7.03, 6.75, 6.74 and 4.67) among which four were olefinic protons (δ_H_ 7.08, 7.03, 6.75, 6.74), and three non-protonated carbons (δ_C_ 176.9, 156.7 and 125.4). ^1^H-^1^H COSY correlation H-2’/H’3’ linked the methine (δ_C_ 55.6) and the methylene. The decadienamide chain is also linked to C-2’ thanks to H-2’/C-1 correlation. A hydroxyphenyl group is determined with the sequence of ^1^H-^1^H COSY cross-peaks H-6’/H-7’/H-8’/H-9’ and the HMBC correlations inside this cycle. The chemical shift of the non-protonated carbon (δ_C_ 156.7) indicated a substitution with an oxygen. This hydroxyphenyl group is linked to C-3’ thanks to the HMBC correlations H-2’/C-4’, H-3’/C-4’ and H-3’/C-9’ and the hydroxyl is placed on ortho position thanks to the correlation H-3’/C-5’. The chemical shift of the last non-protonated carbon (δ_C_ 176.9), the HMBC correlations H-2’/C-1’ and H-3’/C-1’ indicated a carboxylic acid position 1’, which suited the molecular formula of **9**. The configuration of **9**, was deduced by comparison of the predicted ECD spectrum with the experimental one (Figure S70). 2-[(2*E*,8*E*)-deca-2,8-dienamido]-3-(2-hydroxyphenyl)propionic acid was named Colletamide C **(9)**.

**Table S4:** NMR data and key COSY, HMBC and NOESY correlations for the determination of Colletamide C. Correlation in brackets are weaker correlations on the spectrum.

| **Position** | **^1^H : *δ* (ppm)**  **(multiplicity, *J* (Hz))** | **^13^C : *δ* (ppm)** | **COSY (^1^H→^1^H)** | **HMBC**  **(^1^H→^13^C)** | **NOESY (^1^H→^1^H)** |
| --- | --- | --- | --- | --- | --- |
| 1 | - | 168.4 | - | - | - |
| 2 | 5.89 (1H, d, *J* = 15.3) | 124.7 | H-3 H-4 | C-1 C-4 | H-3 H-4 |
| 3 | 6.69 (1H, dt, *J* = 15.2, 7.0) | 145.7 | H-2 H-4 | C-1 C-4 C-5 | H-2 H-4 |
| 4 | 2.17 (2H, ddd, *J* = 14.0, 7.3, 1.2) | 32.9 | H-2 H-3 H-5 | C-2 C-3 C-5 C-6 | H-5 |
| 5 | 1.44 (2H, m) | 28.9 | H-4 H-6 | C-3 C-4 C-6 | H-4 |
| 6 | 1.37 (2H, m) | 30.2 | H-5 H-7 | C-5 C-7 | H-7 |
| 7 | 1.99 (2H, m) | 33.4 | H-6 H-8 | C-5 C-6 C-8 C-9 | H-5 H-6 |
| 8 | 5.41 (2H, m) | 132.3 | H-7 H-10 | C-7 C-10 | H-5 H-10 |
| 9 |  | 125.9 |  |  |  |
| 10 | 1.63 (3H, d, *J* = 3.7) | 18.1 |  | C-8 C-9 |  |
| 1’ | - | 176.9 | - | - | - |
| 2’ | 4.67 (1H, dd, *J* = 8.7, 5.2) | 55.6 | H-3’ | C-1 C-1’ C-3’ C-4’ | H-3’ (H-9’) |
| 3’ | 3.21 (1H, dd, *J* = 13.9, 5.0)  2.98 (1H, dd, *J* = 13.9, 8.9) | 33.6 | H-2’ H-3’ | C-1’ C-2’ C-4’ C-5’ C-9’ | H-2’ H-9’ |
| 4’ | - | 125.4 | - | - | - |
| 5’ | - | 156.7 | - | - | - |
| 6’ | 6.75 (1H, d, *J* = 7.6) | 116.2 | H-7’ | C-4’ C-8’ |  |
| 7’ | 7.03 (1H, td, *J* = 7.6, 1.2) | 128.9 | H-6’ H-8’ | C-5’ C-9’ |  |
| 8’ | 6.74 (1H, t, *J* = 7.4, 1.1) | 120.5 | H-7’ H-9’ | C-6’ C-4’ |  |
| 9’ | 7.08 (1H, dd, *J* = 7.5, 1.2) | 132.2 | H-8’ | C-3’ C-5’ C-7’ |  |

**Figure S63:** ^1^H-NMR (500 MHz, CD_3_OD) of Colletamide C **(9)**

**Figure S64:** ^13^C-NMR (500 MHz, CD_3_OD) spectrum of Colletamide C **(9)**


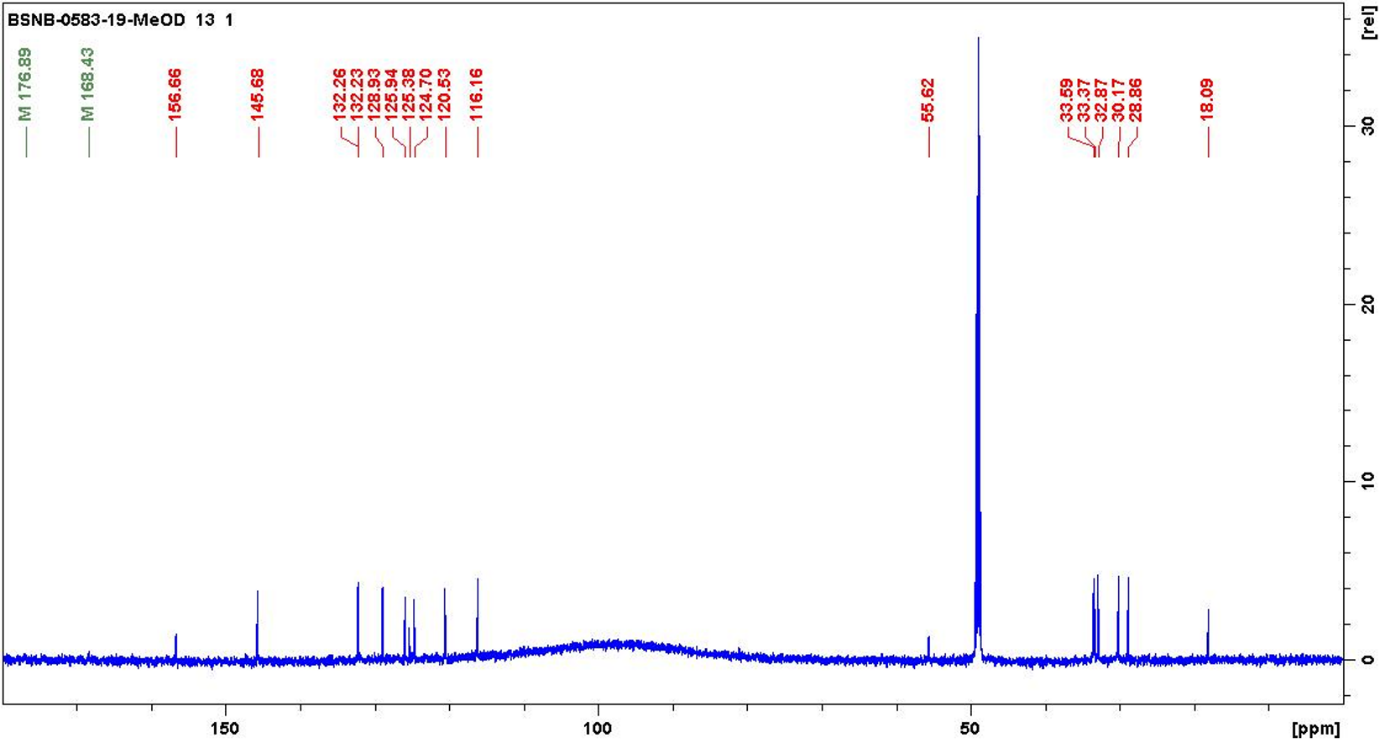


**Figure S65:** ^1^H-^1^H correlations COSY (500 MHz, CD_3_OD) of Colletamide C (**9**)


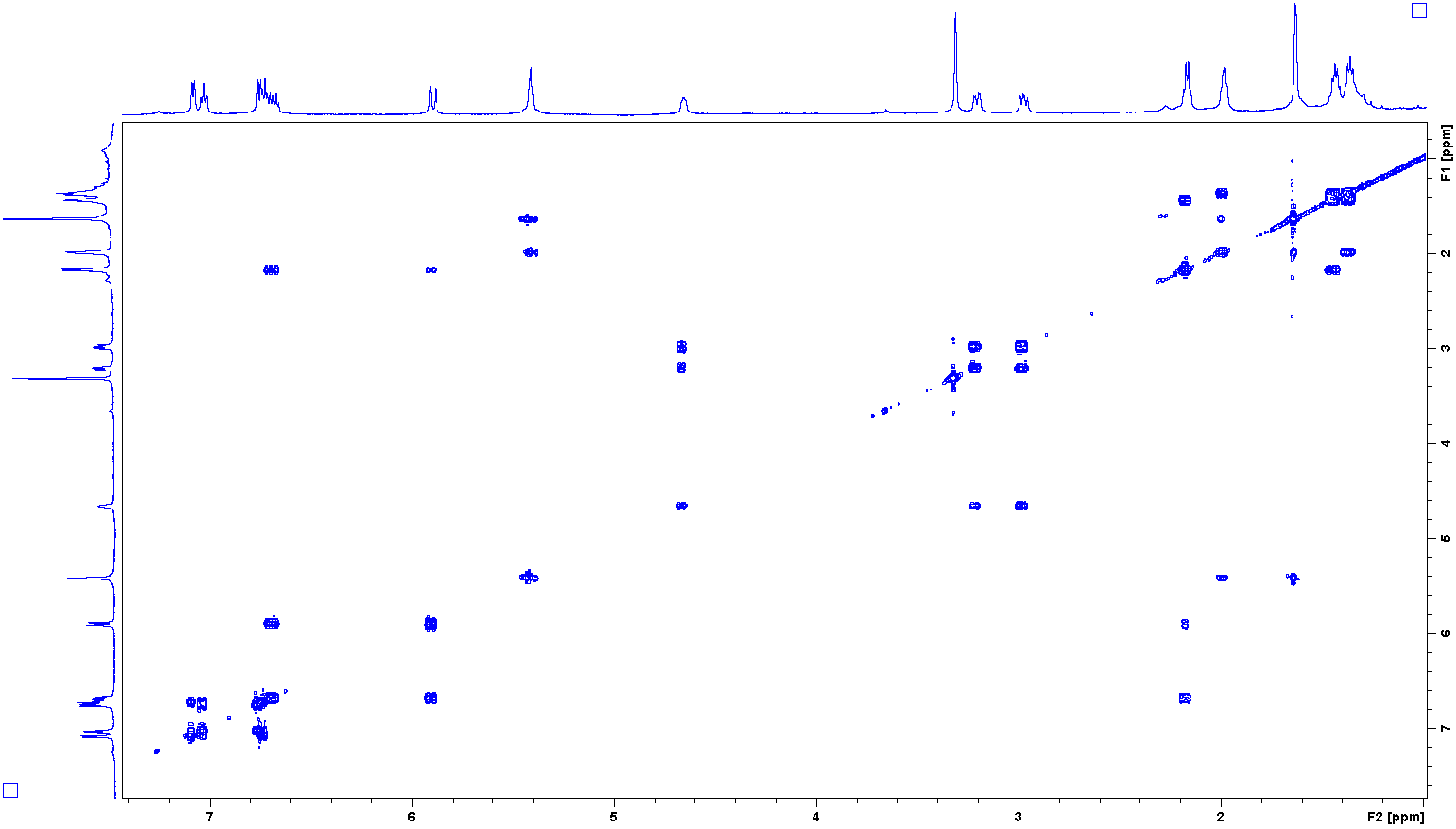


**Figure S66:** ^1^H-^13^C correlations HSQC (500 MHz, CD_3_OD) of Colletamide C (**9**)


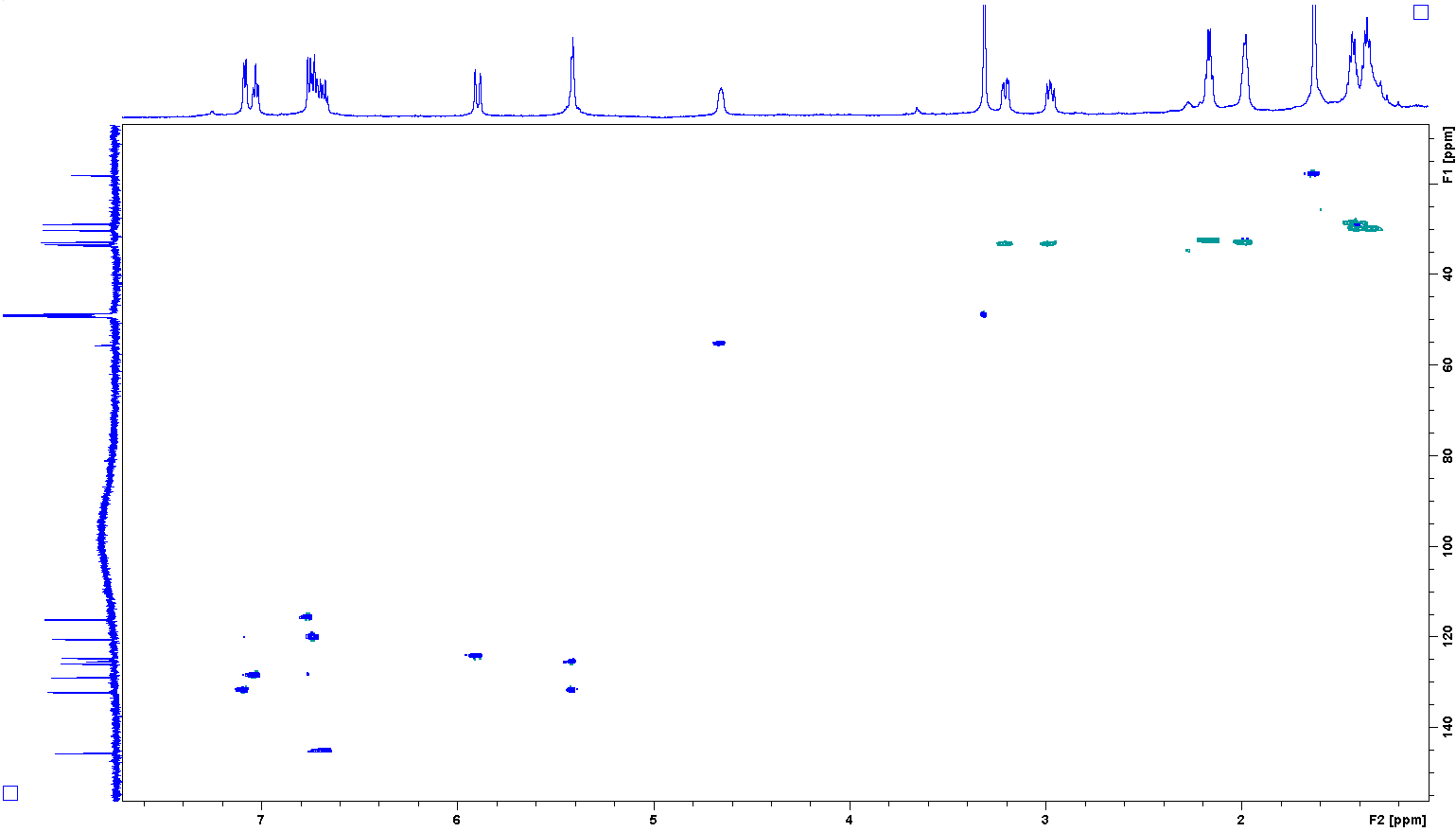


**Figure S67:** ^1^H-^13^C correlations HMBC (500 MHz, CD_3_OD) of Colletamide C (**9**)


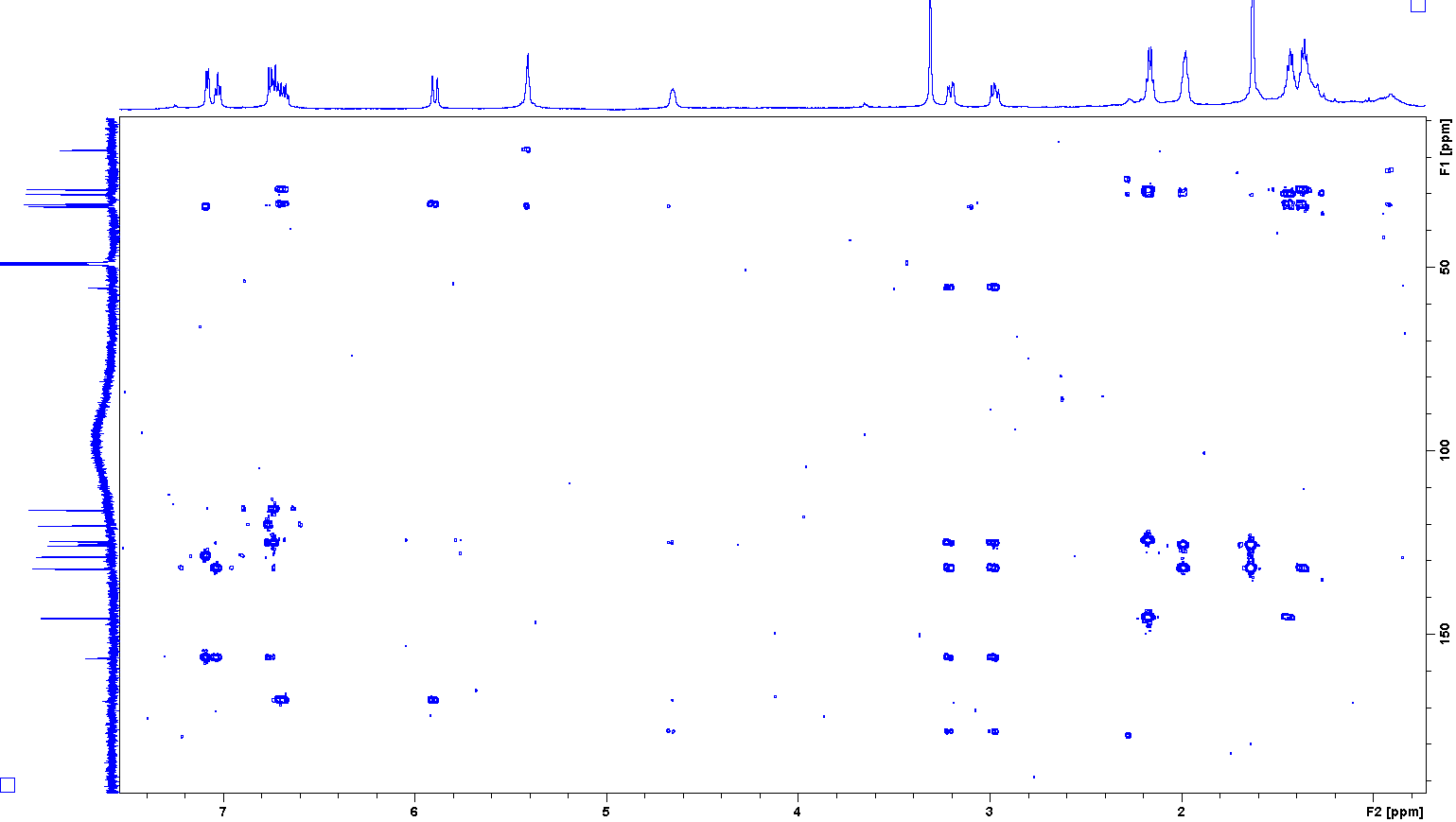


**Figure S68:** ^1^H-^1^H correlations NOESY (500 MHz, CD_3_OD) of Colletamide C (**9**)


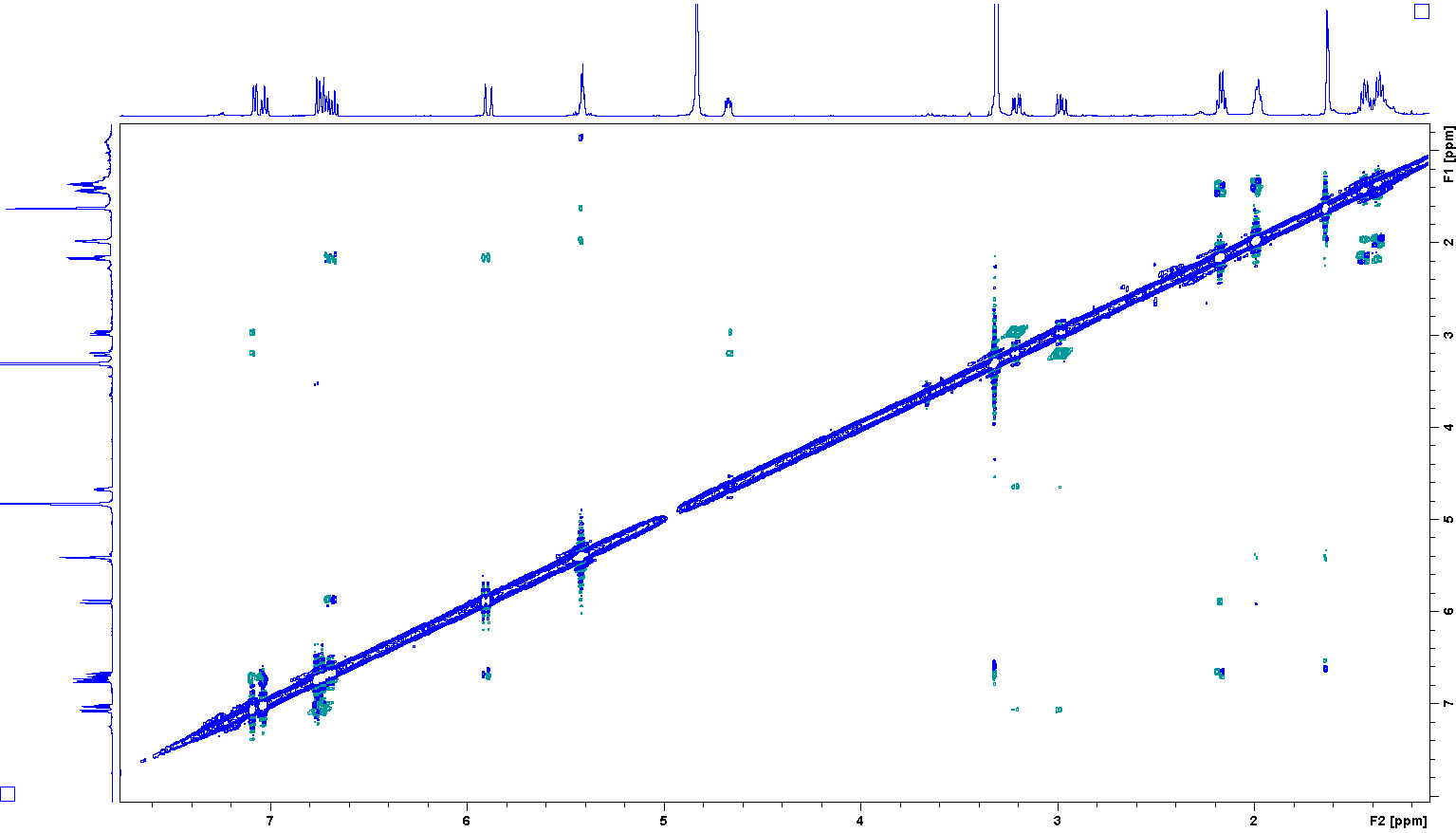


**Figure S69:** MS spectrum of Colletamide C (**9**)


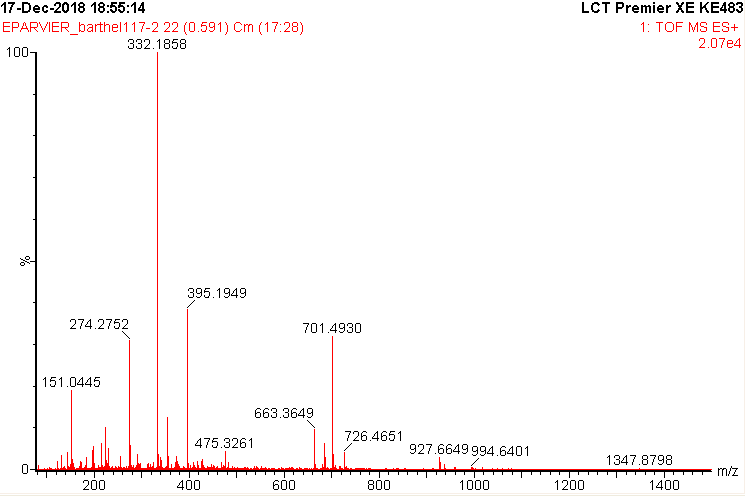


**Figure S70**: Comparison of the predicted ECD spectrum with the experimental spectrum of Colletamide C


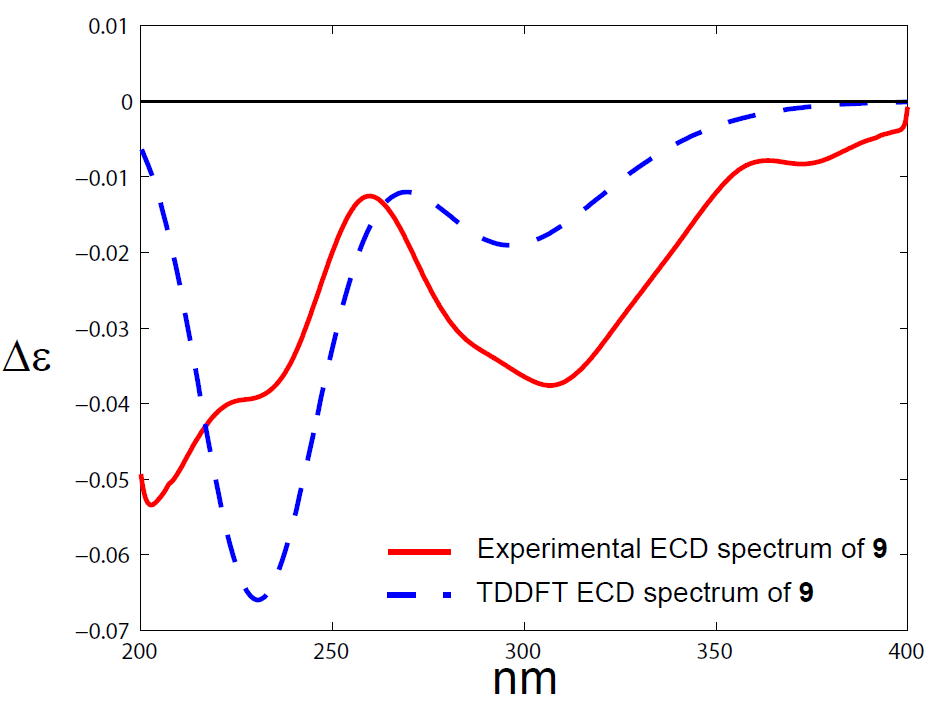


Structural characterization of compound **10**

The molecular formula of compound **(10)** was determined as C_21_H_29_NO_4_ based on the protonated molecule at *m/z* 360.2170 [M+H]^+^ on ESI-HRMS analysis (calcd for C_21_H_30_NO_4_^+^, 360.2169, mass error 0.28 ppm). The same (2*E*,8*E*)-deca-2,8-dienamide chain is recovered thanks to examination of ^1^H, ^13^C, HSQC spectra, ^1^H-^1^H COSY and ^1^H-^13^C HMBC correlations. ^1^H, ^13^C HSQC and HMBC spectra showed the additional presence of two methylenes (δ_H_ 2.97, 2.81 2.30), two methines (δ_H_ 4.14, 4.02), one non-protonated carbon (δ_C_ 179.7) and a phenyl group (δ_H_ 7.25, 7.23, 7.14 and δ_C_ 140.2). The chain H-2’/H-3’/H-4’/H-5’ can be formed thanks to ^1^H-^1^H COSY correlations. Considering the HMBC correlations with H-5’, the phenyl group was placed at position 5’. The chemical shift of H-3’ (δ_C_ 70.1) indicated the presence of an oxygen on this position. HMBC correlation between H-2’ and the non-protonated carbon C-1’ (δ_C_ 179.7) ascertained the presence of a carbonyl group at position 1’. To respect the molecular formula and the chemical shift, a hydroxyl is placed at position 3’ and a carboxylic acid at position 1’. Despite the absence of correlation between H-4’ and C-1 due to the limited amount of compound available, the decadienamide chain was linked on the position 4’ by comparison with chemical shift of previous colletamide A-C. The relative configuration of **10** was determined by comparison of the predicted and experimental NMR chemical shifts (Figure S79). Compound **10** was the 4-[(2*E*,8*E*)-deca-2,8-dienamido)]-3-hydroxy-5-phenylpentanoic acid and was named colletamide D.

**Table S5:** NMR data and key COSY, HMBC and NOESY correlations for the determination of Colletamide D

| **Position** | **^1^H : *δ* (ppm)**  **(multiplicity, *J* (Hz))** | **^13^C : *δ***  **(ppm)** | **COSY**  **(^1^H→^1^H)** | **HMBC**  **(^1^H→^13^C)** | **NOESY**  **(^1^H→^1^H)** |
| --- | --- | --- | --- | --- | --- |
| 1 | - | 168.6 | - | - | - |
| 2 | 5.94 (1H, d, *J* = 15.4) | 124.7 | H-3 H-4 | C-1 C-3 | H-3’ |
| 3 | 6.68 (1H, dt, *J* = 15.3, 7.0) | 145.7 | H-2 H-4 | C-1 C-3 C-4 | H-2’ H-4’ |
| 4 | 2.16 (2H, m) | 32.9 | H-2 H-3 H-5 | C-2 C-3 C-5 |  |
| 5 | 1.44 (2H, m) | 28.9 | H-4 H-6 | C-3 C-4 C-6 |  |
| 6 | 1.34 (2H, m) | 30.2 | H-5 H-7 | C-5 C-7 |  |
| 7 | 1.99 (2H, m) | 33.4 | H-6 H-8 | C-8 C-9 |  |
| 8 | 5.42 (2H, m) | 132.2 | H-7 H-10 | C-7 C-10 |  |
| 9 |  | 126.0 |  |  |  |
| 10 | 1.63 (3H, d, *J* = 4.0) | 18.1 | H-9 | C-8 C-9 |  |
| 1’ | - | 179.7 | - | - | - |
| 2’ | 2.30 (2H, m) | 41.6 | H-3’ | C-1’ |  |
| 3’ | 4.02 (1H, m) | 70.1 | H-2’ H-4’ |  |  |
| 4’ | 4.14 (1H, m) | 56.3 | H-5’ H-3’ |  | H-5’ |
| 5’ | 2.97 (1H, dd, *J* = 13.4, 6.4)  2.81 (1H, dd, *J* = 13.4, 8.4) | 38.3 | H-4’ H-5’ | C-3’ C-4’ C-6’ C-7’ | H-4’ H-5’ |
| 6’ | - | 140.2 | - | - | - |
| 7’ | 7.25 (2H, m) | 130.3 |  | C-7’ C-9’ |  |
| 8’ | 7.23 (2H, m) | 129.3 | H-9’ | C-6’ C-8’ |  |
| 9’ | 7.14 (1H, m) | 127.2 | H-8’ | C-7’ |  |

**Figure S71:** ^1^H-NMR (500 MHz, CD_3_OD) of Colletamide D **(10)**

**Figure S72:** ^13^C-NMR (500 MHz, CD_3_OD) of Colletamide D **(10)**


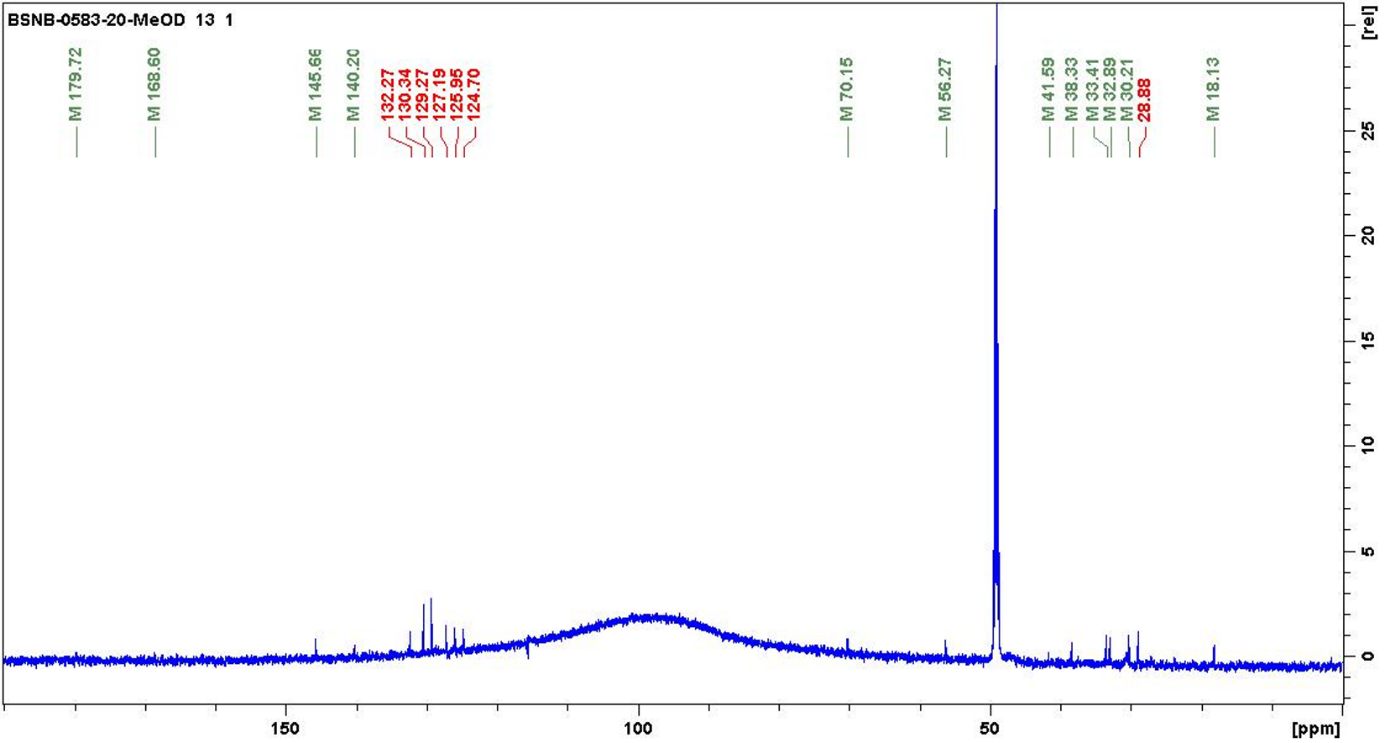


**Figure S73:** ^1^H-^1^H correlations COSY (500 MHz, CD_3_OD) of Colletamide D (**10**)


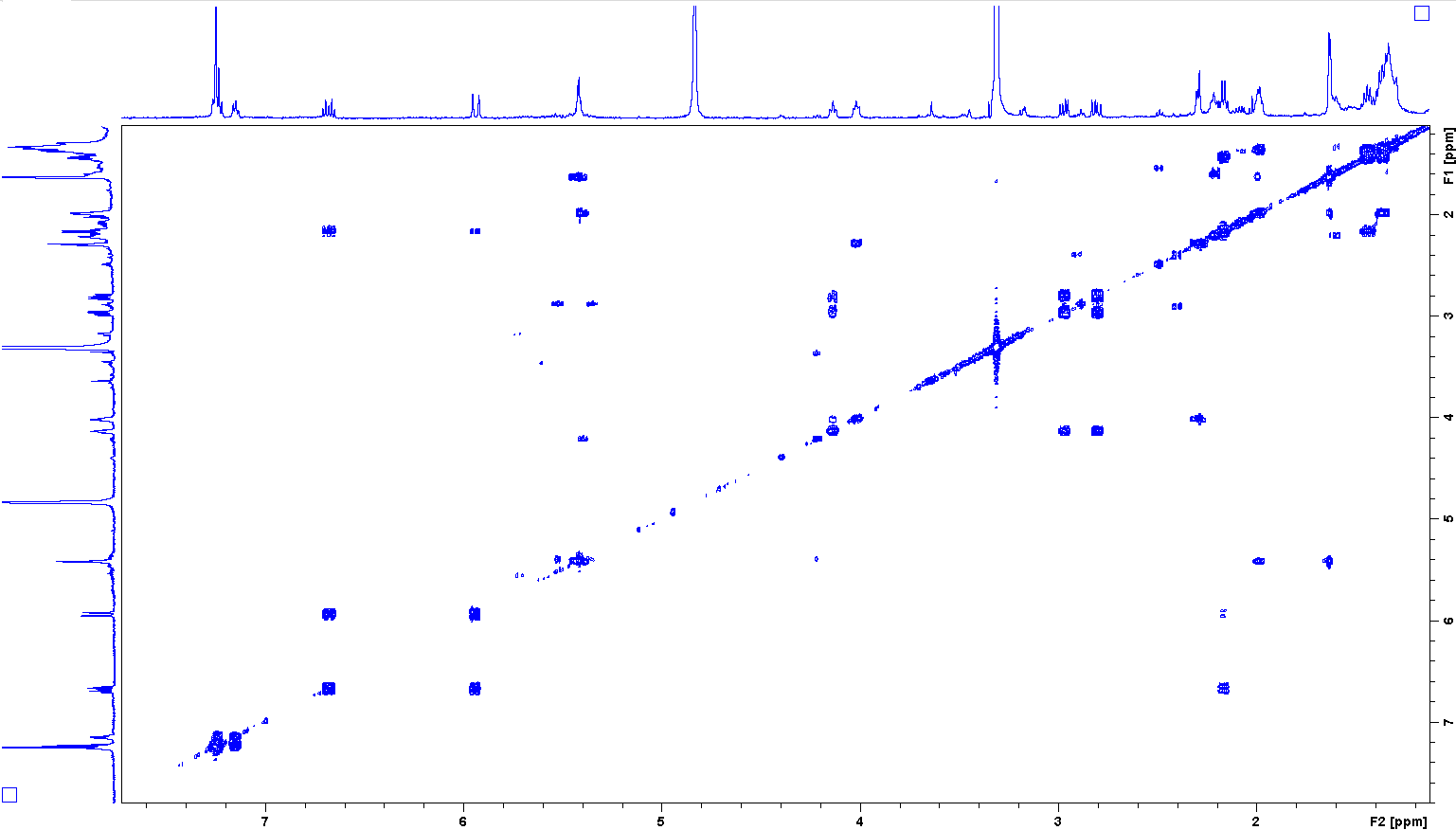


**Figure S74:** ^1^H-^13^C correlations HSQC (500 MHz, CD_3_OD) of Colletamide D (**10**)


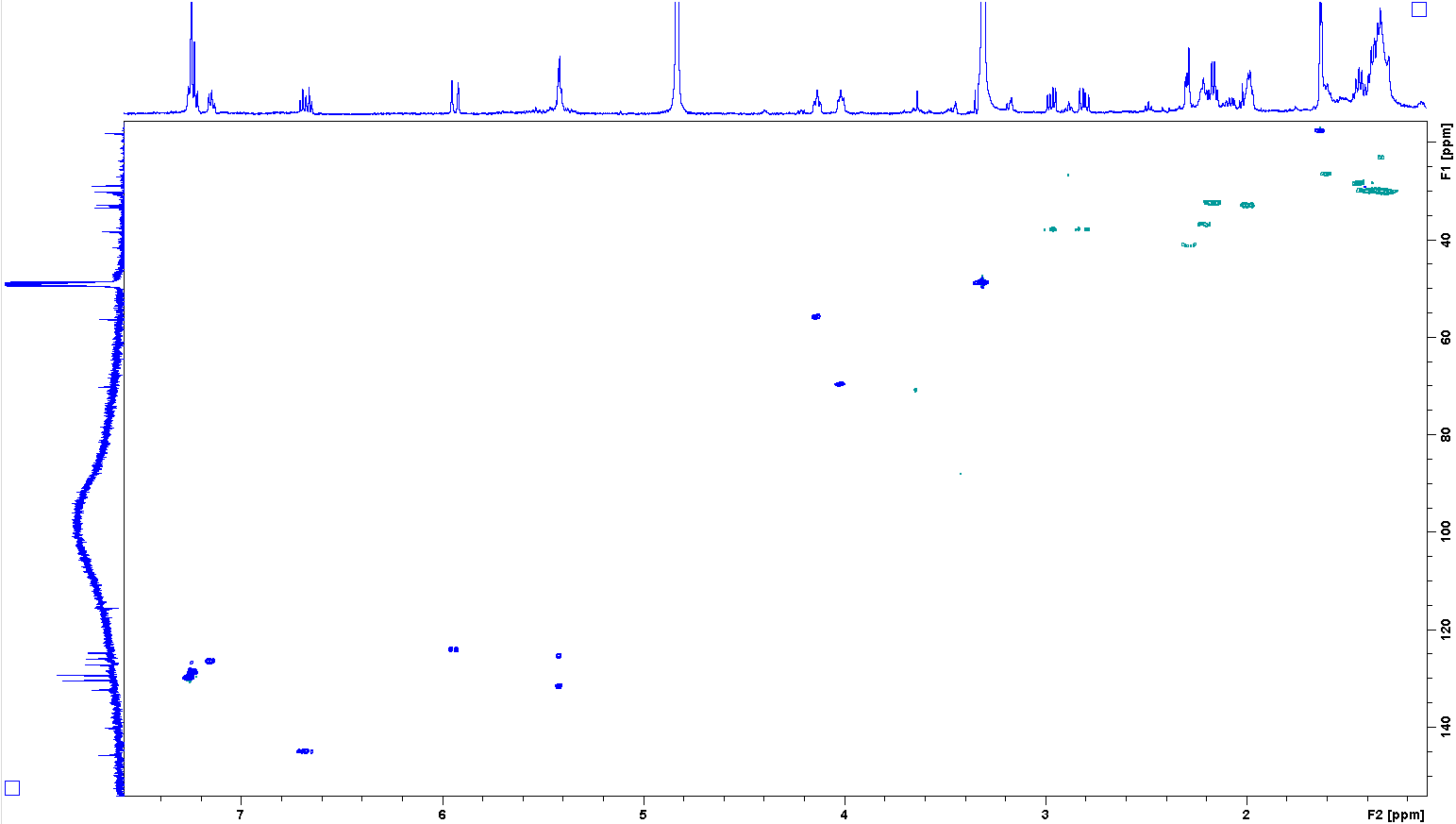


**Figure S75:** ^1^H-^13^C correlations HMBC (500 MHz, CD_3_OD) of Colletamide D (**10**)


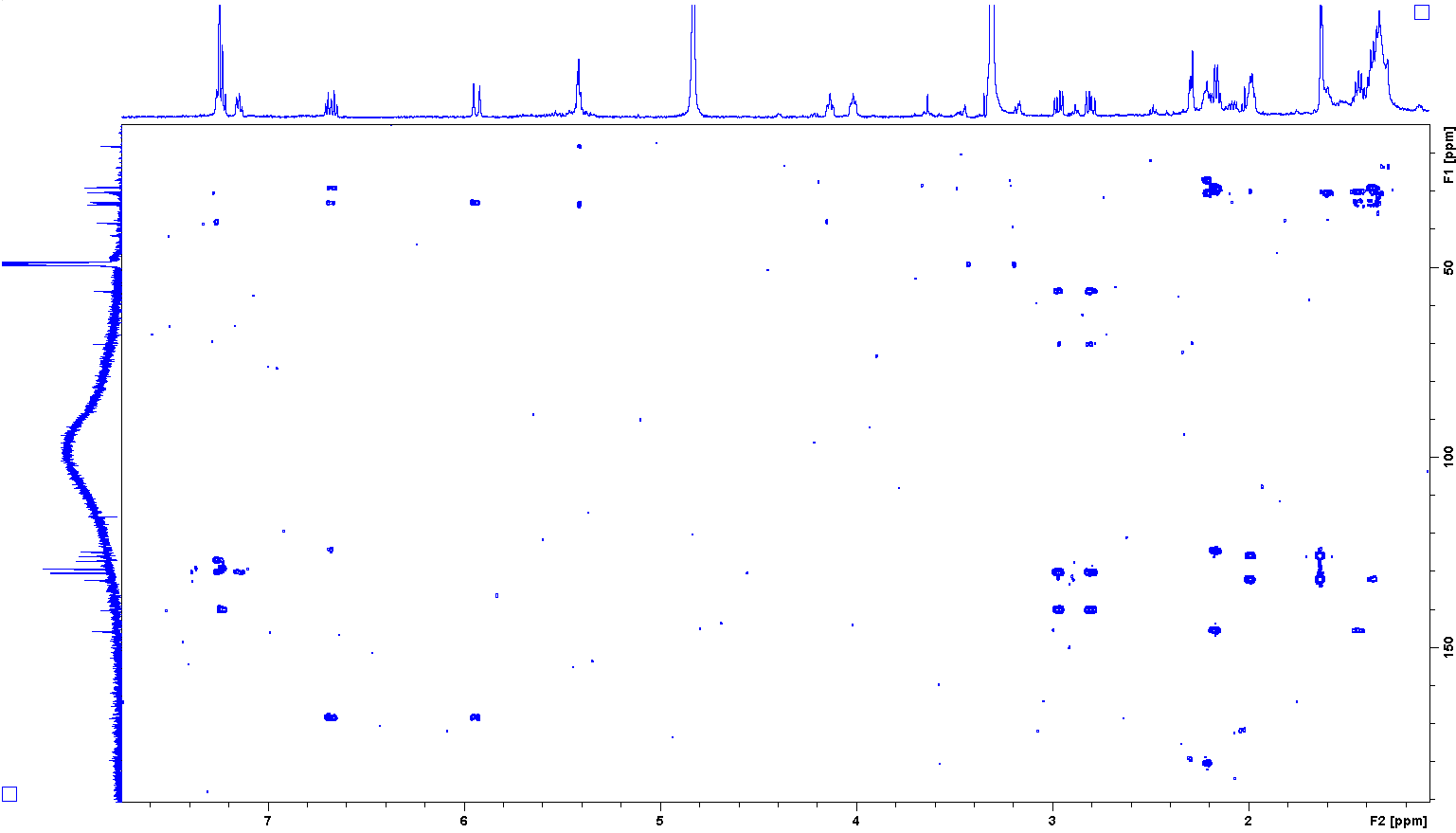


**Figure S76:** ^1^H-^1^H correlations NOESY (500 MHz, CD_3_OD) of Colletamide D (**10**)


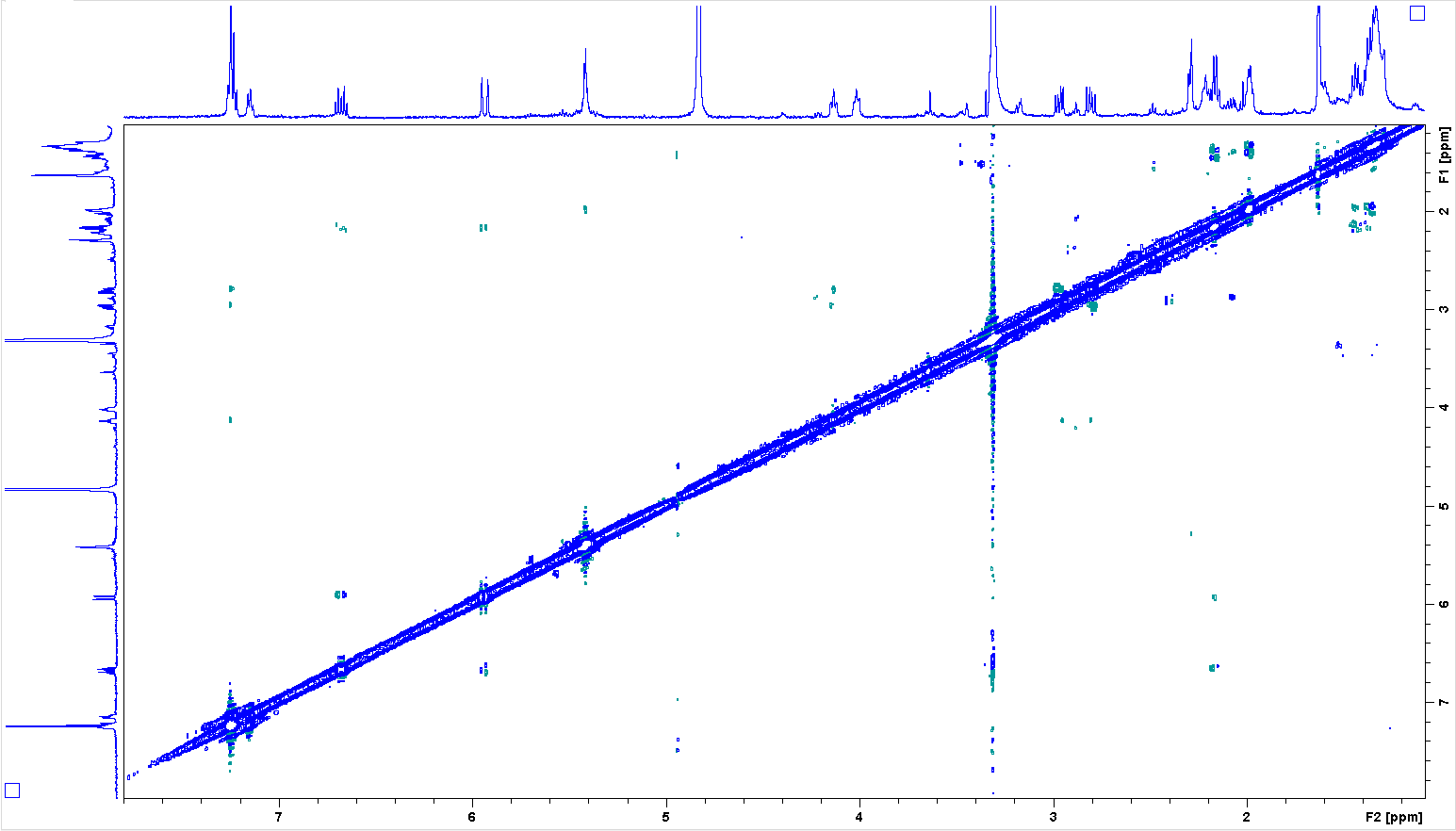


**Figure S77:** Comparison of the predicted and experimental NMR chemical shift of colletamide D isomers **(10)**


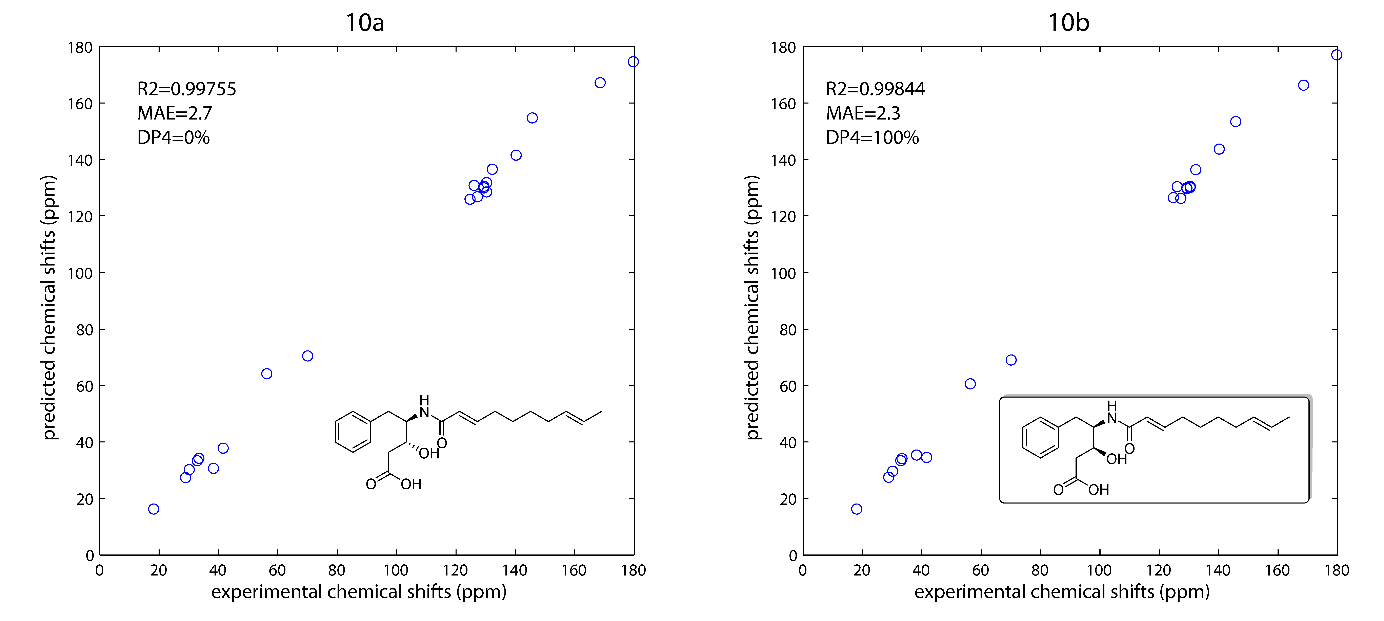


**Figure S78:** MS spectrum of Colletamide D (**10**)


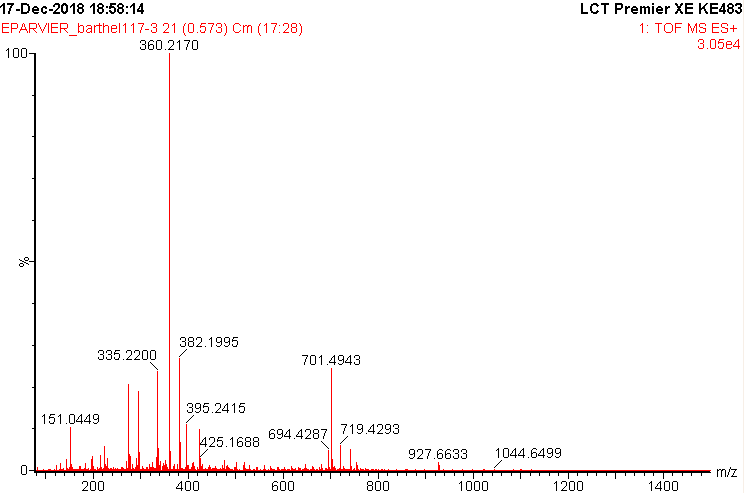


**Figure S79**: Comparison of the predicted and experimental NMR chemical shift of Colletamide D


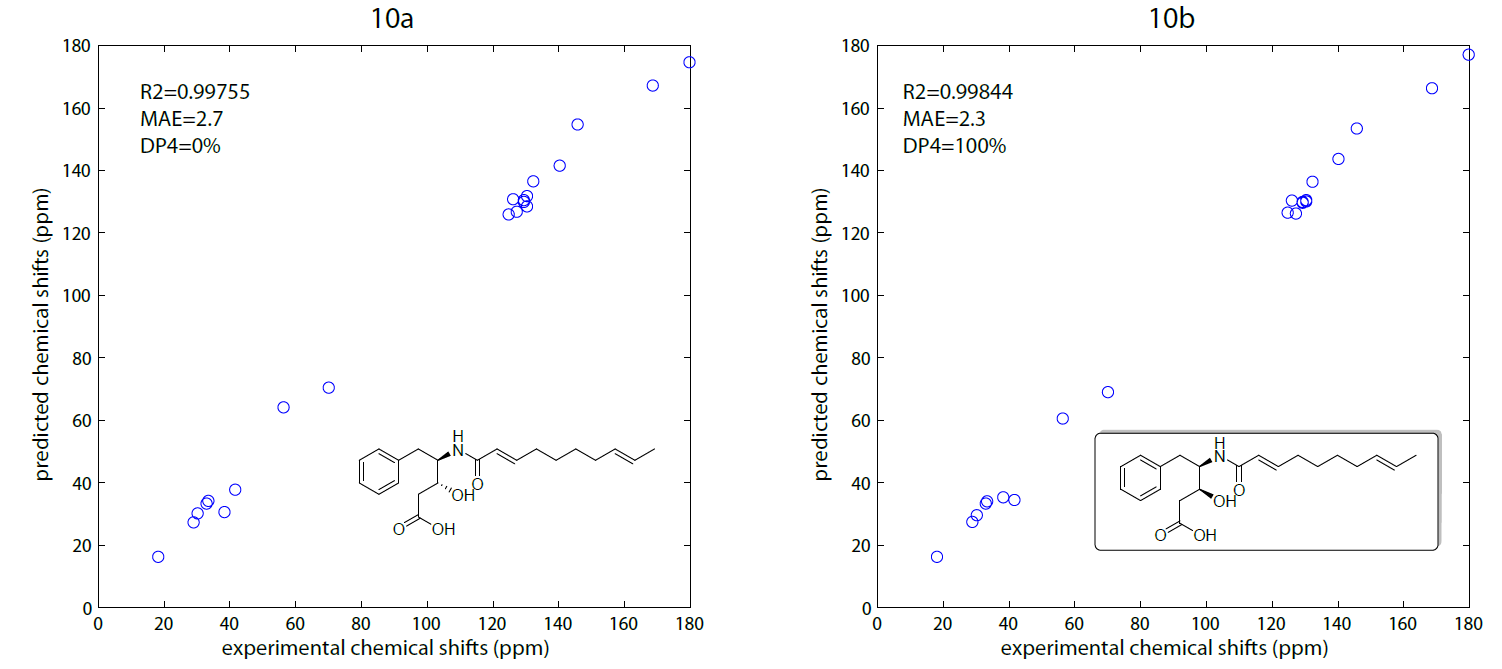


**Figure S80:** MS/MS data of the precursor ion at *m/z* 346

**Table S6:** IC_50_ values of isolated metabolites

| Compound | | IC_50_ (µM) |
| --- | --- | --- |
| 3 | Cyclo-(Phe-Leu-Leu-Leu-Ile) | 17.7 ± 0.12 |
| 1 | Cyclo-(Phe-Leu-Leu-Leu-Val) | 3.24 ± 0.09 |
| 2 | Cyclo-(Phe-Leu-Leu-Leu-Leu) | 1.40 ± 0.12 |
| 4 | Cytochalasine D | 1.97 ± 0.32 |
| 5 | Cytochalasine C | 3.01 ± 0.05 |

**Figure S81**: An ORTEP drawing of the cyclo-(Phe-Leu-Leu-Leu-Ile). Thermal ellipsoids are shown at the 30%


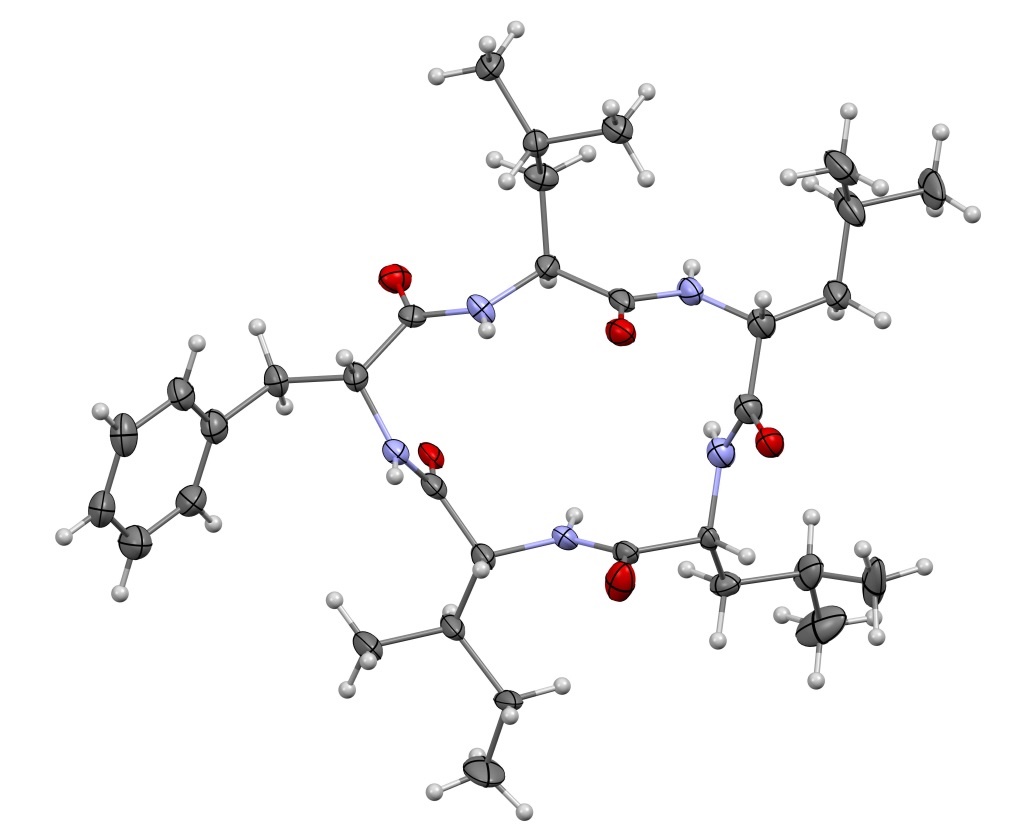


**Table S7**. Crystallographic data and structure refinement details.

| **Compound** | **Cyclo-(Phe-Leu-Leu-Leu-Ile)** |
| --- | --- |
| CCDC | 1944171 |
| Empirical Formula | C_33_ H_53_ N_5_ O_5_ |
| *M_r_* | 599.80 |
| Crystal size, mm^3^ | 0.19 x 0.03 x 0.02 |
| Crystal system | monoclinic |
| Space group | *P* 2_1_ |
| a, Å | 15.29(4) |
| b, Å | 5.015(14) |
| c, Å | 23.41(7) |
| α, ° | 90 |
| β, ° | 105.68(7) |
| γ, ° | 90 |
| Cell volume, Å^3^ | 1728(9) |
| Z ; Z’ | 2 ; 1 |
| T, K | 100 (1) |
| Radiation type ; wavelength Å | CuKα; 1.54178 |
| F_000_ | 652 |
| µ, mm^–1^ | 0.623 |
| *θ* range, ° | 3.002 - 58.669 |
| Reflection collected | 16 929 |
| Reflections unique | 4 331 |
| R_int_ | 0.1723 |
| GOF | 1.028 |
| Refl. obs. (*I*>2σ(*I*)) | 2 082 |
| Parameters | 387 |
| wR_2_ (all data) | 0.1695 |
| R value (*I*>2σ(*I*)) | 0.0828 |
| Largest diff. peak and hole (e-.Å^-3^) | 0.238 ; -0.254 |
